# Global, regional, and national burden of iodine deficiency for women of reproductive age, 1990–2021: a systematic analysis based on the Global Burden of Disease Study 2021

**DOI:** 10.3389/fnut.2025.1577169

**Published:** 2025-09-18

**Authors:** Taotao Wang, Jingmin Tong, Yuzhou Liu, Yanqiu Liu, Hui Xu, Yang Liu

**Affiliations:** ^1^Department of Clinical Nutrition, Affiliated Hospital of Jiangsu University, Zhenjiang, China; ^2^National Key Disciplines of Nutrition and Food Hygiene, Department of Nutrition and Food Hygiene, School of Public Health, Harbin Medical University, Harbin, China; ^3^Department of Obstetrics, Affiliated Hospital of Jiangsu University, Zhenjiang, China; ^4^Department of Epidemiology, School of Public Health, Harbin Medical University, Harbin, China

**Keywords:** iodine deficiency, epidemiology, women of reproductive age, Global Burden of Disease, incidence

## Abstract

**Objective:**

This study aims to assess the global, regional, and national burden of iodine deficiency among women of reproductive age (WRA) from 1990 to 2021.

**Methods:**

Utilizing data from the Global Burden of Disease Study 2021, this study reports age-standardized rates per 100,000 population and average annual percentage changes (AAPCs) in incidence, prevalence, and years lived with disability (YLD) of iodine deficiency among WRA, facilitating a comparative analysis of the burden across regions and nations. Moreover, Joinpoint analysis was utilized to assess temporal trends, the slope index and concentration index of inequality were used to assess the health inequality across countries. The Bayesian age-period-cohort model was employed to forecast the burden up to 2044.

**Results:**

A notable upward trend in the age-standardized incidence rate (ASIR) of iodine deficiency among WRA was observed globally from 1990 to 2021, and this trend is projected to remain relatively stable between 2021 and 2030. The global ASIR increase from 171.3 per 100,000 population (95% CI 122.8, 228.2) in 1990 to 193.6 (95% CI 141.7, 253.2) in 2021 with an AAPC at 0.36 (95% CI 0.26, 0.47). Moreover, the global age-standardized prevalence rate (ASPR) and age-standardized YLD rate (ASYR) demonstrated decreased trend with the AAPC of −0.31 (−0.35, −0.27) and −1.05 (−1.19, −0.92), respectively. In 2021, countries with low sociodemographic index (SDI) exhibited the highest iodine deficiency burden. The highest burden of iodine deficiency was observed in Central Sub-Saharan Africa, while East Asia exhibited the highest increase in ASIR, ASPR and ASYR. Women of 20–24 years showed the highest increase in the age specific incidence rate globally. The significant absolute and relative health inequality was also observed and the gap has been narrowed.

**Conclusions:**

Over the past three decades, there has been an overall upward trend in the ASIR of iodine deficiency among WRA globally. Health inequalities related to iodine deficiency was still prominent across countries. Iodine deficiency among WRA remains a significant public health issue. It is crucial to prioritize the development of effective, targeted strategies and implement monitoring mechanisms to enhance the iodine levels among WRA.

## Introduction

Iodine deficiency adversely affects humans, with endemic goiter being the most visible effect (1). Attention has also been drawn to related disorders such as cretinism and increased infant mortality rates (2). Since 1990, the United Nations World Summit for Children proposed the target of eradicating global iodine deficiency, prompting many countries to implement universal salt iodization (USI) policies (1). However, the sustainable progress of salt iodization policies has coincided with an increase in thyroid disorders. Studies have reported an association between the increased rate of iodine-induced hyperthyroidism and excessive iodized salt consumption (3, 4). Moreover, excessive iodine levels resulting from salt iodization may also contribute to hypothyroidism and autoimmune thyroiditis (5). Concerns regarding iodine-induced thyroid disease could potentially hinder iodine prophylaxis in iodine-deficient populations (6), thereby posing a potential risk for the reemergence of iodine deficiency globally.

According to World Health Organization (WHO), the median urinary iodine concentrations (UIC) in school-age children serves as the most practical indicator for assessing and monitoring iodine levels in the general population (7, 8). However, recent studies indicate that the UIC levels in school-age children do not accurately represent those of vulnerable groups, including women of reproductive age (WRA) (9–12). Studies conducted in various countries have revealed adequate iodine status in school-aged children but persistent iodine deficiency in pregnant women, despite recommendations for supplementation (11–13). Researches have indicated a global and Asian decline in iodine deficiency among the general population and children from 1990 to 2019 (14, 15), whilst iodine status among WRA remains unclear. It is of concern that iodine deficiency in reproductive age or pregnancy is frequently observed in clinical practice, moreover, sub-national studies have demonstrated iodine deficiency among WRA and pregnant women in both developing and developed countries (16–19). However, a paucity of nationally representative UIC surveys among WRA persists (1). As iodine status in WRA and pregnant women are not routinely monitored in antenatal care, iodine deficiency and its consequences may remain insidious. Iodine deficiency during reproductive age might lead to insufficient iodine levels at the onset of pregnancy and throughout gestation, which has been linked to impaired cognitive in offspring, and even result in spontaneous abortion, stillbirth, and cretinism (19), reflecting the urgency of investigating the representative global or national data of iodine deficiency in this vulnerable population to avoid a partial comprehension of the achievement of eliminating iodine deficiency disorders and disregarding the multiple adverse effects. Iodine deficiency disproportionately affects low-income countries (1, 6), and socioeconomic indices have been associated with iodine status in pregnant women (20). However, global cross-national inequalities in iodine deficiency among WRA remain unaddressed.

This study firstly evaluates the global, regional, and national burden of iodine deficiency in WRA by analyzing data from the Global Burden of Diseases Study 2021 (GBD2021). This study examines the global epidemiological patterns of iodine deficiency in WRA, highlighting temporal trends, regional age-related variations, and health inequality to emerge a comprehensive assessment of the iodine deficiency burden globally.

## Materials and methods

### Data source and case definitions

The GBD 2021 provided data on iodine deficiency among WRA of 15–49 years, estimating the incidence, prevalence, mortality, and disability adjusted life years (DALYs) associated with 371 injuries and diseases, covering 95 maternal, neonatal, nutritional and communicable diseases, 234 non-communicable diseases, and 40 injuries, across 204 countries and territories from 1990 to 2021. The categorization of countries and territories encompasses 21 regions and seven super regions on the basis of epidemiological similarities and geographic closeness. GBD 2021 study calculated the socio-demographic indexes (SDI) for all countries. SDI is calculated by considering lag-distributed income per capita, average years of educational attainment, and fertility rates in females under 25 years of age (21). SDI scores were range from 0 to 1, 0 represents lowest income and education experiencing the highest fertility rates, while 1 represents highest income and education experiencing the lowest fertility rates. This study categorizes countries and regions into five SDI levels (low, low-middle, middle, high-middle, and high SDI) to analyze the correlation between iodine deficiency rate and socioeconomic development. Data is freely available via the Global Health Data Exchange at https://ghdx.healthdata.org/gbd-2021/sources. The data processing and statistical modeling methods for burden estimation in GBD 2021 have been previously detailed (22). In terms of case definitions, the non-fatal burden of iodine deficiency as estimated by GBD 2021 encompasses visible goiter (grade 2) induced by iodine deficiency as well as the other associated outcomes, including thyroid dysfunction, heart failure, and cretinism. However, non-visible goiter (grade 1) and sub-clinical iodine deficiency caused by iodine deficiency were not estimated (23). We extracted annual data on the number and rate of incidence, prevalence and years lived with disability (YLD) of iodine deficiency from 1990 to 2021.

### Cross-country health inequality analysis

The health inequality concerning the burden of iodine deficiency was conducted using the absolute slope index and the concentration index (23). The slope index represented the absolute disparity in health inequity for the burden of iodine deficiency between individuals with the highest and lowest level of education or wealth (23), while the concentration index reflected relative health inequity. The calculation of the slope index involved performing a regression analysis of age-standardized incidence, prevalence, and YLDs associated with iodine deficiency against the SDI–linked relative social position scale. The concentration index was ascertained by the cumulative relative distribution of the persons, ranked according to the SDI, with a Lorenz concentration curve representing the iodine deficiency burden, followed by numerically integration to determine the area under the curve (24).

### Projections of iodine deficiency burden to 2044

The Bayesian Age-Period-Cohort (BAPC) model was employed to analyze and forecast trends in the burden of iodine deficiency up to 2044. BAPC uses integrated nested Laplace approximations (INLA) for full Bayesian inference (25, 26). All BAPC analyses were conducted using the BAPC and INLA packages in the R statistical software (version 4.4.1).

### Statistical analysis

The age-standardized rates and corresponding 95% confidence intervals (95% CI) were calculated applying direct standardization method by calling the ageadjust.direct function from epitools based on the world standard population from the GBD Study 2021 (21, 27). Rates are presented per 100,000 population. The Joinpoint regression model was utilized to identify trends in age-standardized incidence, prevalence and YLD for iodine deficiency among WRA (28). We employed a log-linear model for piecewise regression and utilized the grid search method to identify all potential joinpoints, the grid point with the smallest model mean squared error was selected as the joinpoint. Additionally, we determined the optimal number of joinpoints via Monte Carlo permutation tests. Finally, using the optimal model, we quantified the annual change trends from 1990 to 2019 by calculating the annual percent change (APC) and their 95% CI for each segment and the average annual percent change (AAPC) for the entire period. The AAPC summarizes the overall trend within a predetermined, fixed period by calculating a weighted average of the APCs. Joinpoint regression analysis identifies the year of significant changes, aiding in the evaluation of underlying causes for dramatic shifts in iodine deficiency burden. We assessed the iodine deficiency burden in seven age groups (15–19, 20–24, 25–29, 30–34, 35–39, 40–44, and 45–49 years) and five SDI categories. Locally weighted linear regression model was applied to explore the relations between age-standardized rates and SDI. Data were analyzed using R Studio software (version 4.4.1). The *P*-value was determined at a significance level of 0.05.

## Results

### Global burden of iodine deficiency for WRA in 2021

**Figure 1:**
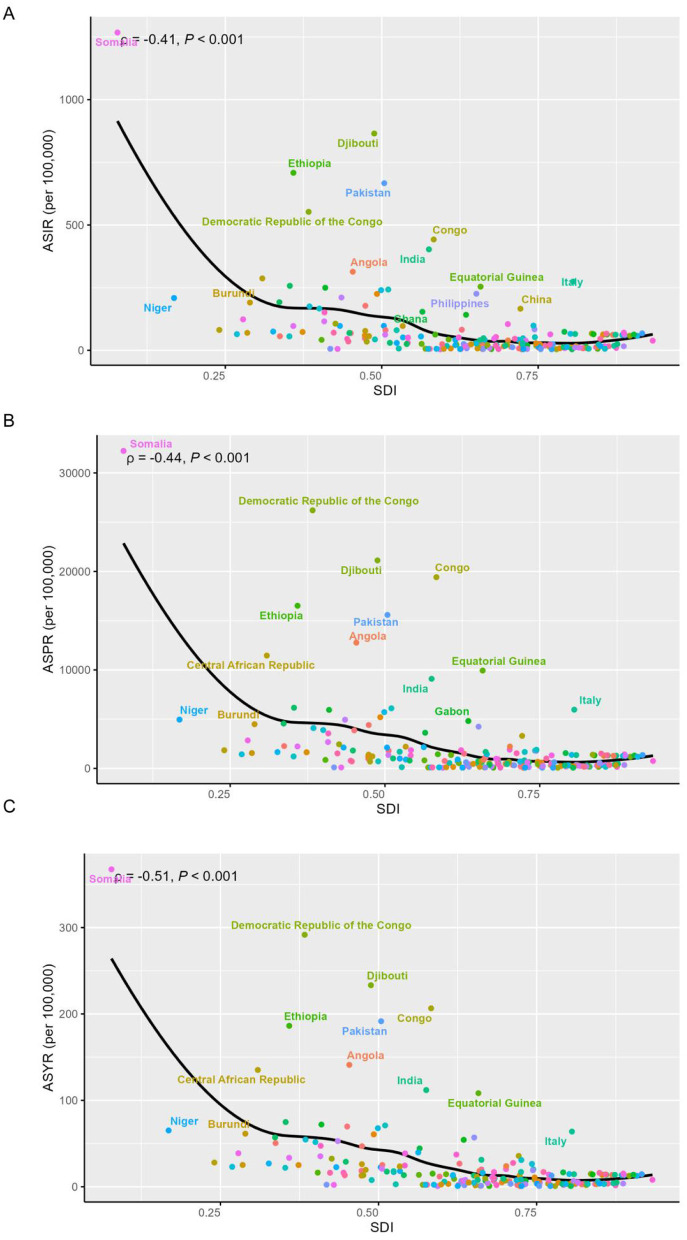
Association between SDI and age-standardized rates of iodine deficiency among WRA in 2021. **(A)** Age-standardized incidence rate; **(B)** age-standardized prevalence rate **(C)** age-standardized YLD rate. Dotted lines refer to the global level of rates.

GBD 2021 estimated that, 2,503,319.6 new cases [95% uncertainty interval (UI) 1,800,060.7, 3,324,741.5], 61,931,118.2 (95% UI 50,490,007.5, 74,375,422.6) prevalent cases and 1,016,939.8 (95% UI 522,768, 1,811,645.9) YLD of iodine deficiency were recorded to have occurred in WRA globally (Table 1). In 2021, the global age-standardized incidence rate (ASIR), age-standardized prevalence rate (ASPR) and age-standardized YLD rate (ASYR) per 100,000 population were 193.6 (95% CI 141.7, 253.2), 4,265.5 (95% CI 3,373.9, 5,221.2), and 52 (95% CI 26.8, 92.5; Table 1). As shown in Figure 1, the ASIR, ASPR, and ASYR showed negative correlations with SDI. Low SDI countries demonstrated the highest ASIR (338.6, 95% CI 246.4, 443.1), ASPR (9,261.8, 95% CI 7,483.7, 11,248.7), and ASYR (114.7, 95% CI 60.7, 200.8; Table 1, Figure 1). Further, over 90% of the overall incident cases were concentrated in individuals aged 15–34 years globally (Supplementary Figure S1A). The globally age specific incidence rate of iodine deficiency decreased with age, with the highest rate observed in the 15–19 and 20–24 age groups (Supplementary Table S2, Supplementary Figure S3A). A similar trend was also noted across the five SDI regions. Whilst individuals aged 15–19 exhibited the lowest age specific prevalence rate (Supplementary Table S2, Supplementary Figure S3B) and age specific YLD rate (Supplementary Table S2, Supplementary Figure S3C), these rates did not differ significantly among other age groups (Supplementary Table S2, Supplementary Figure S3C).

**Table 1:** Iodine deficiency burden among WRA from 1990 to 2021 at the global and regional level.

**Location**	**Incidence number 1990 (95% UI)**	**ASIR 1990 (95% CI)**	**Incidence number 2021 (95% UI)**	**ASIR 2021 (95% CI)**	**AAPC (95% CI)**	***P-*value**
Global	2,503,319.6 (1,800,060.7–3,324,741.5)	171.3 (122.8–228.2)	3,646,587 (2,665,698.9–4,773,411.1)	193.6 (141.7–253.2)	0.36 (0.26–0.47)	< 0.001
High SDI	73,125.5 (45,073.1–107,909.9)	33.1 (20.5–48.6)	71,346.5 (44,600.3–105,094.9)	31.6 (20–46.1)	−0.14 (−0.16 to −0.12)	< 0.001
High-middle SDI	296,542.5 (212,591.2–400,102.6)	102.1 (73.1–138.2)	265,491.2 (191,891.5–352,185.2)	102.7 (74.8–135)	0.01 (−0.06 to 0.07)	0.836
Middle SDI	675,136.4 (473,347.8–920,403.6)	133.5 (93.2–182.9)	851,136.4 (624,074.1–1,119,771.3)	147.8 (108.6–194.1)	0.31 (0.23–0.4)	< 0.001
Low-middle SDI	940,167.1 (678,042.2–1,239,088.2)	299.9 (215.2–397.1)	1,379,776.8 (1,011,230.5–1,798,974.8)	259.6 (190.1–339)	−0.47 (−0.61 to −0.32)	< 0.001
Low SDI	517,547.2 (391,138.6–660,796.3)	393.5 (296.2–504.4)	1,077,952.1 (786,964.7–1,406,462.4)	338.6 (246.4–443.1)	−0.49 (−0.63 to −0.34)	< 0.001
High-income Asia Pacific	9,171.5 (5,178.8–14,261.2)	20.3 (11.5–31.5)	6,264.6 (3,489.9–9,738.6)	17.5 (9.8–26.9)	−0.48 (−0.52 to −0.45)	< 0.001
High-income North America	11,920.5 (6,675.2–18,481)	16.3 (9.2–25.2)	13,218.8 (7,388.3–20,560.2)	16.1 (9–24.9)	−0.03 (−0.07 to 0.01)	0.099
Western Europe	106,110.6 (73,426.1–144,967.4)	115.2 (80.1–156.9)	73,802.3 (48,848.8–104,648.3)	89.5 (59.9–125.4)	−0.81 (−0.86 to −0.75)	< 0.001
Australasia	816 (449.2–1,281.8)	15.3 (8.5–24)	1,011.9 (556.9–1,579.6)	14.5 (8–22.5)	−0.18 (−0.2 to −0.16)	< 0.001
Andean Latin America	608.4 (337–964.9)	6.2 (3.4–10)	912.6 (488.3–1,470.7)	5.2 (2.8–8.4)	−0.55 (−0.58 to −0.53)	< 0.001
Tropical Latin America	3,314.3 (1,826.9–5,243.1)	8.1 (4.4–12.8)	4,421.1 (2,370.9–7,056.2)	7.5 (4.1–11.9)	−0.25 (−0.27 to −0.22)	< 0.001
Central Latin America	16,034.6 (9,575.4–24,176.6)	34.8 (20.5–53.2)	22,422.9 (13,171.9–34,302)	33.3 (19.7–50.8)	−0.14 (−0.23 to −0.06)	0.001
Southern Latin America	1,114.2 (627.9–1,769.6)	8.9 (5–14.2)	1,290.3 (698.9–2,074)	7.6 (4.1–12.1)	−0.52 (−0.53 to −0.51)	< 0.001
Caribbean	4,397.8 (2,670–6,418.2)	43.4 (26.2–63.8)	4,309 (2,347.2–6,518.6)	36.4 (19.9–55)	−0.56 (−0.69 to −0.44)	< 0.001
Central Europe	5,643.3 (3,185.6–8,704.9)	18.8 (10.6–28.8)	3,309.5 (1,811.4–5,218.1)	13.9 (7.7–21.5)	−1 (−1.1 to −0.9)	< 0.001
Eastern Europe	6,506.9 (3,660.1–10,158.6)	12.2 (7–19)	5,352.6 (2,998.9–8,415.6)	12.5 (7.2–19.3)	0.05 (−0.07 to 0.16)	0.435
Central Asia	5,678.2 (3,469–8,635.2)	31.2 (18.9–47.9)	5,673.5 (3,250.9–8,894.6)	24.4 (14.1–38.1)	−0.84 (−1.04 to −0.64)	< 0.001
North Africa and Middle East	45,485.6 (26,840.8–72,184.6)	55.3 (31.9–88.6)	63,657.6 (36,586.9–100,104.1)	40 (23.1–62.9)	−1.05 (−1.13 to −0.97)	< 0.001
South Asia	1,257,497.8 (896,132.9–1,666,720)	435 (309–578.3)	2,126,235.6 (1,569,229.9–2,761,919.9)	414.3 (305.6–538.7)	−0.16 (−0.3 to −0.02)	0.023
Southeast Asia	202,405.9 (149,912.2–261,955.3)	149.4 (110–194.7)	152,638.2 (108,267.5–202,738.5)	86.4 (61.4–114.5)	−1.76 (−1.83 to −1.7)	< 0.001
East Asia	431,587.7 (296,813.9–593,982.6)	116.7 (80.1–161.4)	429,422.6 (319,237.5–555,016.5)	159.5 (119–204.8)	1.01 (0.87–1.14)	< 0.001
Oceania	176.9 (97.6–269.9)	10.6 (5.8–16.4)	240.6 (135.7–372.6)	6.8 (3.8–10.5)	−1.44 (−1.52 to −1.36)	< 0.001
Western Sub-Saharan Africa	79,987.9 (54,190.5–110,284.9)	157.3 (105.4–219.5)	142,731.7 (90,731.9–204,374.1)	106.7 (67.3–154.3)	−1.26 (−1.35 to −1.17)	< 0.001
Eastern Sub-Saharan Africa	199,578.3 (148,577.6–258,505.6)	381.9 (283.9–495.7)	390,665.4 (290,091.9–508,010)	309.8 (229.5–403.9)	−0.72 (−0.86 to −0.58)	< 0.001
Central Sub-Saharan Africa	102,172.5 (83,806.9–124,239.4)	681.1 (555.8–831.4)	181,895.3 (114,130–267,007.7)	471.5 (291.5–696.8)	−1.18 (−1.27 to −1.09)	< 0.001
Southern Sub-Saharan Africa	13,110.5 (9,551.8–17,194)	88.6 (63.3–118.2)	17,110.9 (10,482.4–25,112.7)	78.4 (47.9–115.2)	−0.42 (−0.51 to −0.33)	< 0.001
Global	61,931,118.2 (50,490,007.5–74,375,422.6)	4,676.4 (3,811–5,625.3)	83,610,103.9 (66,102,675–102,389,372.3)	4,265.5 (3,373.9–5,221.2)	−0.31 (−0.35 to −0.27)	< 0.001
High SDI	1,602,455.1 (1,185,329–2,082,338.8)	696.5 (514.5–905.3)	1,644,190.6 (1,233,135.4–2,116,696)	655.3 (489.5–843.3)	−0.2 (−0.21 to −0.19)	< 0.001
High-middle SDI	6,580,178.7 (5,187,581.7–8,113,882.8)	2,376.3 (1,873.8–2,932.5)	7,280,276.7 (5,654,935.1–9,045,881.3)	2,253.2 (1,754.5–2,791.4)	−0.18 (−0.25 to −0.11)	< 0.001
Middle SDI	14,978,086.7 (11,758,804.6–18,488,026.8)	3,446.4 (2,705.2–4,264.6)	20,181,311.6 (15,766,901.8–24,936,674.2)	3,192 (2,495.9–3,940.7)	−0.25 (−0.3 to −0.19)	< 0.001
Low-middle SDI	25,467,567.7 (20,861,031.1–30,316,586.5)	9,601 (7,858.2–11,460.9)	30,226,222.6 (23,774,355–37,090,445.3)	6,035.8 (4,745.4–7,411.1)	−1.5 (−1.54 to −1.45)	< 0.001
Low SDI	13,285,643.4 (11,248,174–15,512,028.5)	12,397.5 (10,476.6–14,510.1)	24,259,076.5 (19,641,988.5–29,418,262.5)	9,261.8 (7,483.7–11,248.7)	−0.95 (−1.03 to −0.87)	< 0.001
High-income Asia Pacific	200,657.4 (147,550.6–265,612.3)	437.7 (321.7–579.1)	142,244.5 (103,647.4–188,726.6)	365.9 (265–484.3)	−0.58 (−0.58 to −0.57)	< 0.001
High-income North America	256,929.5 (185,512.5–340,812.1)	341.7 (245.6–453.9)	285,701.9 (206,791.8–379,018.5)	336.7 (242.9–446.6)	−0.04 (−0.05 to −0.03)	< 0.001
Western Europe	2,419,055.2 (1,860,706.8–3,028,524)	2,488.9 (1,914.5–3,113.9)	1,872,466.1 (1,412,844.3–2,377,801)	1,921.2 (1,445.2–2,438.2)	−0.83 (−0.89 to −0.78)	< 0.001
Australasia	17,272 (12,329.5–23,108.8)	320.4 (228.3–428.8)	21,973.7 (15,646.7–29,207.7)	300.1 (212.6–399.1)	−0.21 (−0.23 to −0.18)	< 0.001
Andean Latin America	11,713.6 (8,068.3–16,028.7)	126.1 (87.5–172.3)	17,916.6 (12,045.7–25,110.9)	102.5 (68.8–143.7)	−0.66 (−0.71 to −0.62)	< 0.001
Tropical Latin America	65,765.7 (45,466.8–89,221.8)	166.6 (115.8–225.8)	95,238.3 (66,462.1–129,031.7)	155.6 (108–211.1)	−0.23 (−0.26 to −0.2)	< 0.001
Central Latin America	462,293.2 (344,132.6–585,061.5)	1,106.1 (828.2–1,400.4)	761,605.3 (572,918–966,875.1)	1,116.1 (839–1,417)	0.02 (−0.04 to 0.07)	0.577
Southern Latin America	30,420.7 (21,794.4–40,262.4)	244.7 (175.6–323.9)	33,107.8 (23,040.3–44,437.8)	190.3 (132–255.7)	−0.82 (−0.84 to −0.8)	< 0.001
Caribbean	102,564.9 (77,280.7–129,774.6)	1,114 (840.5–1,410.7)	106,704.9 (79,921.5–137,335.2)	883.7 (661.4–1,137.3)	−0.76 (−0.89 to −0.63)	< 0.001
Central Europe	148,410.3 (113,748.9–188,939.3)	482.8 (369.7–614.6)	83,232.7 (60,829.4–110,009.7)	320.2 (232.1–423.3)	−1.35 (−1.45 to −1.25)	0.013
Eastern Europe	247,088.9 (181,392.9–319,921.9)	448.5 (327.9–581.4)	222,621.5 (164,835.4–287,459.1)	471.2 (345.9–609.1)	0.15 (0.03–0.27)	< 0.001
Central Asia	259,003.3 (194,666.5–324,304.8)	1,511.1 (1,139.9–1,893.1)	228,314.1 (169,398.6–292,000.5)	943.5 (697.9–1,207.6)	−1.55 (−1.72 to −1.38)	< 0.001
North Africa and Middle East	1,897,873.7 (1,442,683.8–2,384,828.3)	2,439.7 (1,869–3,055.5)	2,221,074.7 (1,636,702.7–2,837,811.2)	1,396.5 (1,027.7–1,784.8)	−1.79 (−1.95 to −1.63)	< 0.001
South Asia	34,227,653.7 (27,822,092.3–40,978,626.2)	13,726.7 (11,148.9–16,476.8)	46,066,064.2 (36,220,355.4–56,674,436.4)	9,386.2 (7,376.9–11,552.9)	−1.23 (−1.28 to −1.18)	< 0.001
Southeast Asia	3,527,330.6 (2,799,332.8–4,307,683.9)	3,030 (2,405.1–3,704.8)	2,767,588.4 (2,098,020.1–3,495,312.9)	1,493.4 (1,132–1,885.4)		< 0.001
East Asia	8,168,708 (6,122,493–10,444,018.7)	2,510.7 (1,882.2–3,215.5)	11,291,359.3 (8,672,808.8–14,050,564.5)	3,185 (2,446.5–3,957)	0.77 (0.63–0.9)	< 0.001
Oceania	3,047.4 (2,126.8–4,145.3)	202.1 (141.8–274.6)	4,201.8 (2,887.1–5,755.5)	122.4 (84.3–167.6)	−1.64 (−1.71 to −1.56)	< 0.001
Western Sub-Saharan Africa	1,645,610.7 (1,287,293–2,026,343.2)	4,001.1 (3,133.4–4,940.2)	2,746,283.3 (2,069,050.5–3,476,176.6)	2,416.4 (1,825.1–3,061.8)	−1.64 (−1.79 to −1.49)	< 0.001
Eastern Sub-Saharan Africa	4,134,911.4 (3,326,795.8–5,027,814.4)	10,304.9 (8,268–12,543.9)	7,346,595 (5,819,319.5–9,025,564.5)	7,267.2 (5,745.4–8,938.3)	−1.16 (−1.36 to −0.96)	< 0.001
Central Sub-Saharan Africa	3,758,142.6 (3,462,976.1–4,078,658.2)	31,120.6 (28,691.8–33,770.2)	6,927,457.6 (5,612,387.2–8,292,466.2)	21,601.3 (17,527.8–25,844.8)	−1.17 (−1.29 to −1.05)	< 0.001
Southern Sub-Saharan Africa	346,665.4 (307,129.4–393,348.7)	2,679.8 (2,375.9–3,043.7)	368,352 (277,531.5–471,100.9)	1,694.9 (1,278.2–2,167.3)	−1.49 (−1.61 to −1.36)	< 0.001
Global	960,796.3 (580,971.3–1,571,698.1)	71.9 (43.4–117.9)	1,016,939.8 (522,768–1,811,645.9)	52 (26.8–92.5)	−1.05 (−1.19 to −0.92)	< 0.001
High SDI	17,460.3 (7,628.3–34,078.4)	7.6 (3.3–14.8)	17,917.3 (7,957.7–34,832.8)	7.2 (3.2–13.9)	−0.2 (−0.21 to −0.18)	< 0.001
High-middle SDI	79,317.6 (39,868.2–144,359)	28.6 (14.4–52.2)	83,827.1 (40,851–156,345.7)	26.1 (12.8–48.5)	−0.29 (−0.36 to −0.22)	< 0.001
Middle SDI	246,880.1 (146,065.9–404,708)	56.1 (33.1–91.9)	230,148.9 (112,227.6–427,735.2)	36.5 (17.8–67.7)	−1.37 (−1.45 to −1.3)	< 0.001
Low-middle SDI	431,311.6 (270,026.6–683,651.9)	160 (99.8–254.3)	380,995.8 (201,000.6–672,132.6)	75.8 (39.9–133.9)	−2.42 (−2.59 to −2.25)	< 0.001
Low SDI	185,572.3 (108,449.7–312,580)	171 (99.3–289.3)	303,786.3 (161,359–529,538.8)	114.7 (60.7–200.8)	−1.29 (−1.36 to −1.23)	< 0.001
High-income Asia Pacific	2,164.4 (905.2–4,253.5)	4.7 (2–9.3)	1,535.6 (635.4–3,027.2)	4 (1.6–7.8)	−0.57 (−0.59 to −0.56)	< 0.001
High-income North America	2,759.5 (1,142–5,400.5)	3.7 (1.5–7.2)	3,049.9 (1,263.9–6,033.7)	3.6 (1.5–7.1)	−0.06 (−0.08 to −0.04)	< 0.001
Western Europe	25,905.4 (11,780.3–49,711.9)	26.7 (12.1–51.1)	20,031.2 (9,082.5–38,717.3)	20.6 (9.3–39.7)	−0.83 (−0.89 to −0.77)	< 0.001
Australasia	184.2 (77.3–374.3)	3.4 (1.4–6.9)	235.2 (93.7–479.6)	3.2 (1.3–6.6)	−0.2 (−0.26 to −0.14)	< 0.001
Andean Latin America	261.6 (133.4–446.1)	2.8 (1.4–4.8)	235.3 (104.7–450.8)	1.3 (0.6–2.6)	−2.4 (−2.59 to −2.21)	< 0.001
Tropical Latin America	773.5 (337.3–1,484.5)	2 (0.9–3.8)	1,014.5 (402.9–2,027.7)	1.7 (0.7–3.3)	−0.53 (−0.59 to −0.48)	< 0.001
Central Latin America	6,284.8 (3,258.9–11,412.9)	15 (7.8–27.3)	9,810.8 (5,029.2–17,944.9)	14.4 (7.4–26.3)	−0.21 (−0.48 to 0.07)	0.138
Southern Latin America	326 (131.8–639.7)	2.6 (1.1–5.1)	356.2 (135.7–724.8)	2 (0.8–4.2)	−0.81 (−0.85 to −0.76)	< 0.001
Caribbean	1,643.4 (797–2,901)	17.7 (8.7–31.3)	1,784.1 (874.6–3,163.6)	14.8 (7.2–26.3)	−0.6 (−0.75 to −0.44)	< 0.001
Central Europe	1,880.5 (935.8–3,413.8)	6.1 (3–11.1)	954.7 (423.8–1,846.6)	3.7 (1.6–7.1)	−1.66 (−1.82 to −1.51)	< 0.001
Eastern Europe	4,536.6 (2,100.9–7,938.2)	8.2 (3.8–14.3)	4,149.9 (1,902.4–7,127.9)	8.6 (3.9–14.9)	0.19 (0.03–0.35)	0.022
Central Asia	3,929.8 (2,041.6–6,832.8)	22.9 (11.9–39.8)	3,464.9 (1,754.9–6,167.4)	14.3 (7.3–25.5)	−1.53 (−1.71 to −1.36)	< 0.001
North Africa and Middle East	35,216 (20,172–56,948.2)	45 (25.8–72.8)	39,471.4 (21,915.3–64,609.7)	24.8 (13.8–40.6)	−1.92 (−2.04 to −1.8)	< 0.001
South Asia	596,284.3 (375,454.4–937,574.4)	235.5 (148–371.2)	566,358.6 (293,964.7–1,000,709.6)	115.1 (59.7–203.7)	−2.32 (−2.46 to −2.18)	< 0.001
Southeast Asia	60,669.1 (35,473.1–98,328.3)	51.4 (30–83.5)	39,994.8 (21,684–68,996.9)	21.6 (11.7–37.3)	−2.79 (−3.08 to −2.51)	0.001
East Asia	103,218.8 (52,584.6–189,401.4)	31.6 (16.1–58.1)	122,114.1 (56,194–236,357.1)	34.5 (15.8–66.7)	0.3 (0.2–0.4)	< 0.001
Oceania	65.6 (35.5–111.3)	4.3 (2.4–7.3)	88.4 (46.4–153.3)	2.6 (1.4–4.5)	−1.68 (−1.72 to −1.64)	< 0.001
Western Sub-Saharan Africa	20,175.9 (10,405.5–36,243.4)	48.5 (24.9–87.5)	34,310.4 (17,645.3–62,854.2)	29.8 (15.3–54.9)	−1.58 (−1.7 to −1.46)	< 0.001
Eastern Sub-Saharan Africa	49,592.4 (25,686.1–89,221.1)	122.2 (63–221)	86,600.5 (43,991.3–155,886.8)	84.9 (43–153.3)	−1.21 (−1.38 to −1.03)	< 0.001
Central Sub-Saharan Africa	40,920.1 (20,039.1–73,594.1)	337.6 (165.1–607.8)	7,7281.8 (36,256–14,2814.6)	240.1 (113–445.1)	−1.1 (−1.22 to −0.97)	< 0.001
Southern Sub-Saharan Africa	4,004.3 (1,986.9–7,150.3)	30.8 (15.3–55.1)	4,097.5 (1,941.7–7,764.6)	18.8 (8.9–35.7)	−1.6 (−1.7 to −1.5)	< 0.001

Rates are expressed per 100,000 persons.

ASIR, age-standardized incidence rate; ASPR, age-standardized prevalence rate; YLD, years lived with disability; ASYR, age-standardized YLD rate; SDI, socio-demographic index; AAPC, average annual percent change.

### Regional and national burden of iodine deficiency for WRA

**Figure 2:**
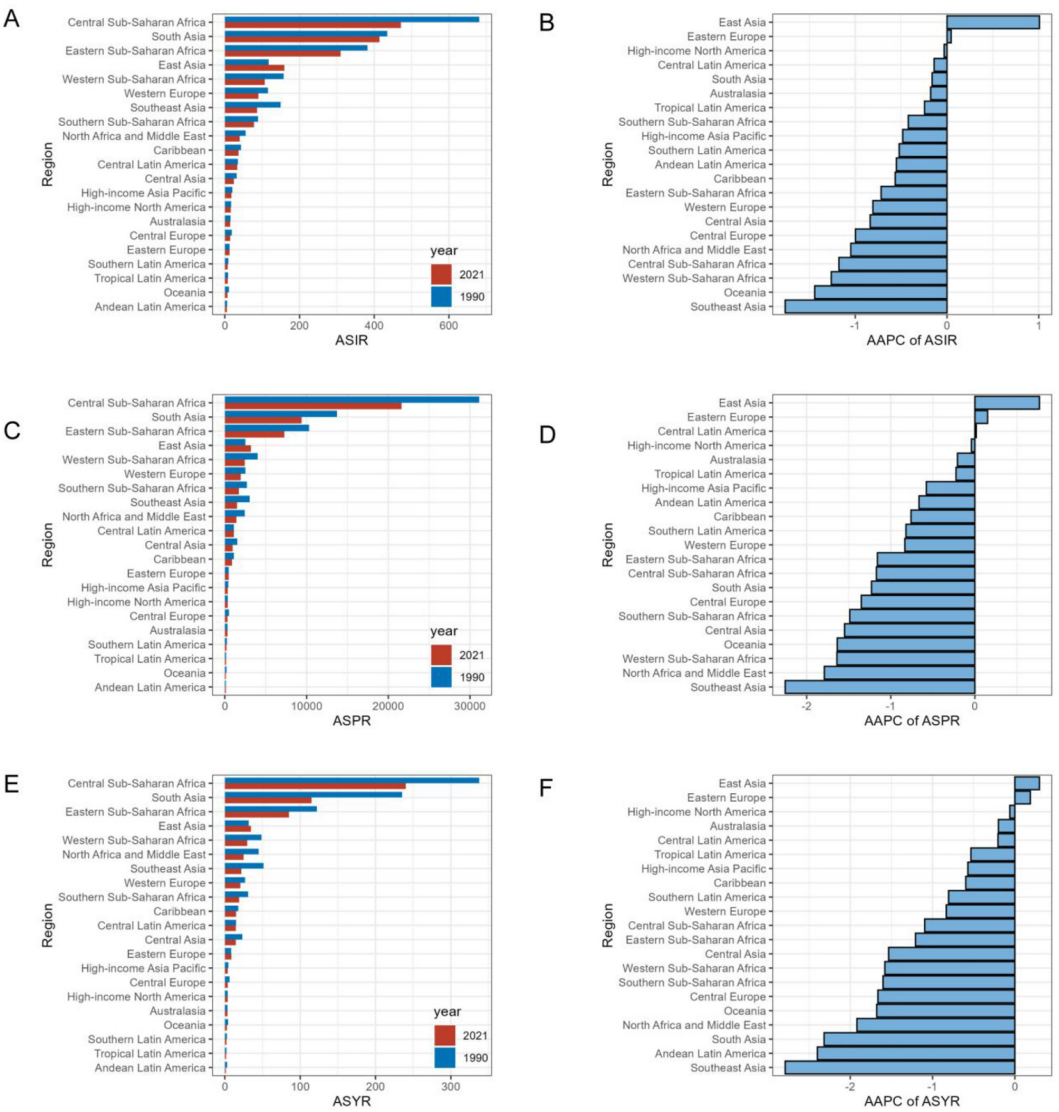
Regional age-standardized rates of iodine deficiency among WRA in 2021 and corresponding AAPC in rates from 1990 to 2021. Rates are expressed per 100,000 populations. **(A)** ASIR in 1990 and 2021; **(B)** AAPC of ASIR; **(C)** ASPR in 1990 and 2021; **(D)** AAPC of ASPR; **(E)** ASYR in 1990 and 2021; **(F)** AAPC of ASYR. AAPC: average annual percent change.

Central Sub-Saharan Africa exhibited the highest iodine deficiency burden across the 21 regions, followed by South Asia (Table 1, Figure 2). In 2021, the highest ASIR, ASPR, and ASYR of iodine deficiency were all noted in Central Sub-Saharan Africa, at 471.5 (95% CI 291.5, 696.8), 21,601.3 (95% CI 17,527.8, 25,844.8), and 240.1 (95% CI 113.0, 445.1), respectively. By contrast, Andean Latin America exhibited the lowest ASIR (5.2, 95% CI 2.8, 8.4) and ASYR (1.3, 95% CI 0.6, 2.6) in 2021, followed by Oceania and Tropical Latin America (Table 1, Figures 2A, C, E).

At the national level, Somalia reported the highest ASIR of iodine deficiency in 2021, at 1,267.6 (983.2, 1,562.7), followed by Djibouti, Ethiopia, and Pakistan (Figure 1A, Supplementary Table S1). Somalia also showed the highest ASPR at 32,237.8 (25,911.1, 38,381) and ASYR at 367.4 (181.2, 659.1), followed by Democratic Republic of the Congo, all of which belong to low or low-middle SDI regions (Figures 1B,C, Supplementary Table S1). Notably, the highest burden of iodine deficiency among WRA in middle, high-middle, and high SDI countries was observed in Italy (Figure 1, Supplementary Table S1).

### Temporal trend of iodine deficiency for WRA from 1990 to 2021

**Figure 3:**
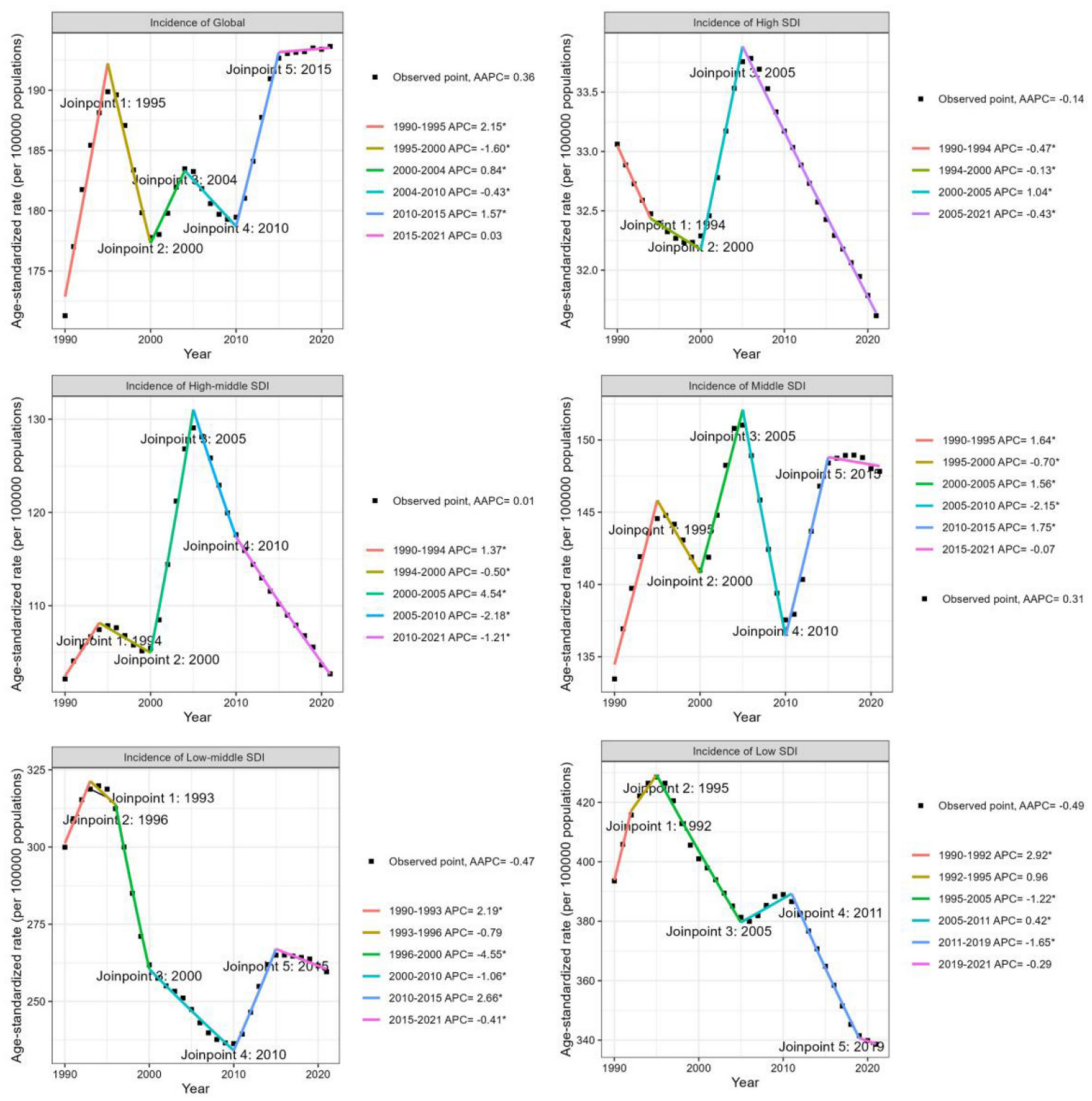
Joinpoint regression analysis of trends in the age-standardized incidence rate of iodine deficiency among WRA globally and by SDI quintiles from 1990 to 2021.

Between 1990 and 2021, the global incidence number of iodine deficiency exhibited an overall increase (Supplementary Figure S2A), rising by approximately 1.1 million (Table 1). Since 1990, the global ASIR of iodine deficiency also increase significantly, however, the upward trend was mainly driven by 8.33% of the 204 countries. The global ASIR increased from 171.3 per 100,000 population (95% CI 122.8, 228.2) in 1990 to 193.6 (95% CI 141.7, 253.2) in 2021, concerning the AAPC of 0.36 (95% CI 0.26, 0.47; Table 1, Figure 3). More specifically, the most remarkable increase of global ASIR was observed between 1990 and 1995 (APC = 2.15, *P* < 0.05), followed by a decline between 1995 and 2000 (APC = −1.6, *P* < 0.05), a moderate increase between 2000 and 2004 (APC = 0.84, *P* < 0.05), and a gradual decrease between 2004 and 2010 (APC = −0.43, *P* < 0.05).

Subsequently, a notable rise occurred between 2010 and 2015 (APC = 1.57, *P* < 0.05), followed by a consistent slight increase over the subsequent decade (APC = 0.03; Figure 3). Overall, the global ASPR of iodine deficiency demonstrated a declining trend [AAPC = −0.31 (95% CI −0.35, −0.27)] (Table 1, Supplementary Figure S4). Notably, a slight but consistent upward trend in ASPR has been observed since 2010 (Supplementary Figure S4). Similar to the global ASIR experiencing a downward trajectory, the global ASYR for iodine deficiency has decreased at an AAPC of −1.05 (95% CI −1.19, −0.92) over the past three decades, with the most pronounced reduction occurred between 2006 and 2009 (APC = −2.56, *P* < 0.05; Table 1, Supplementary Figure S5).

Analyzed by SDI category, the ASIR of iodine deficiency increased in middle SDI countries [AAPC: 0.31 (95% CI 0.23, 0.4)], closely mirroring the global trend. As shown in Figure 3, declining trends in ASIR for iodine deficiency were observed in high, low-middle, and low SDI countries, with the average annual percentage changes (AAPCs) of −0.14 (95% CI −0.16, −0.12), −0.47 (95% CI −0.61, −0.32), and −0.49 (95% CI −0.63, −0.34), respectively (Table 1, Figure 3). In high-middle SDI countries, the ASIR has remained stable during the past three decades (Table 1, Figure 3). Furthermore, although the ASIR decreased in low and low-middle SDI countries, the overall incidence number increased significantly, especially in low SDI countries, where new cases approximately doubled (Table 1, Supplementary Figure S2A). Countries in all SDI quintiles exhibited declining trends in ASPR and ASYR since 1990. Low-middle SDI countries exhibited the most significant decline in ASPR and ASYR, with AAPCs of −1.5 (95% CI −1.54, −1.45) and −2.42 (95% CI −2.59, −2.25), respectively (Supplementary Figures S4, S5). Additionally, middle-SDI countries displayed a significant upward trend in ASPR during 2010–2015 [annual percentage change (APC) = 0.89, *P* < 0.05; Supplementary Figure S4), consistent with global patterns.

At the regional level, the greatest rise in ASIR and ASPR of iodine deficiency has been consistently observed in East Asia over the past 32 years (Figures 2B, D). The ASIR in East Asia increased from 116.7 per 100,000 population (95% CI 80.1, 161.4) to 159.5 (95% CI 119, 204.8), respectively, at the AAPC of 1.01 (95% CI 0.87, 1.14; Table 1). The ASPR increased from 2,510.7 (95% CI 1,882.2, 3,215.5) to 3,185.0 (95% CI 2,446.5, 3,957), at an AAPC of 0.77 (95% CI 0.63, 0.9). Similarly, East Asia also showed the highest increase in ASYR with a modest yet significant trend [AAPC = 0.3, (95% CI 0.2, 0.4)] (Table 1, Figure 2F). The iodine deficiency burden has been decreased in most regions, in which Southeast Asia consistently exhibited the most significant reduction. Specifically, Southeast Asia had AAPCs of −1.76 (95% CI −1.83, −1.7) for ASIR, −2.26 (95% CI −2.39, −2.13) for ASPR and −2.79 (95% CI −3.08, −2.51) for ASYR (Table 1, Figures 2B, D, F).

**Figure 4:**
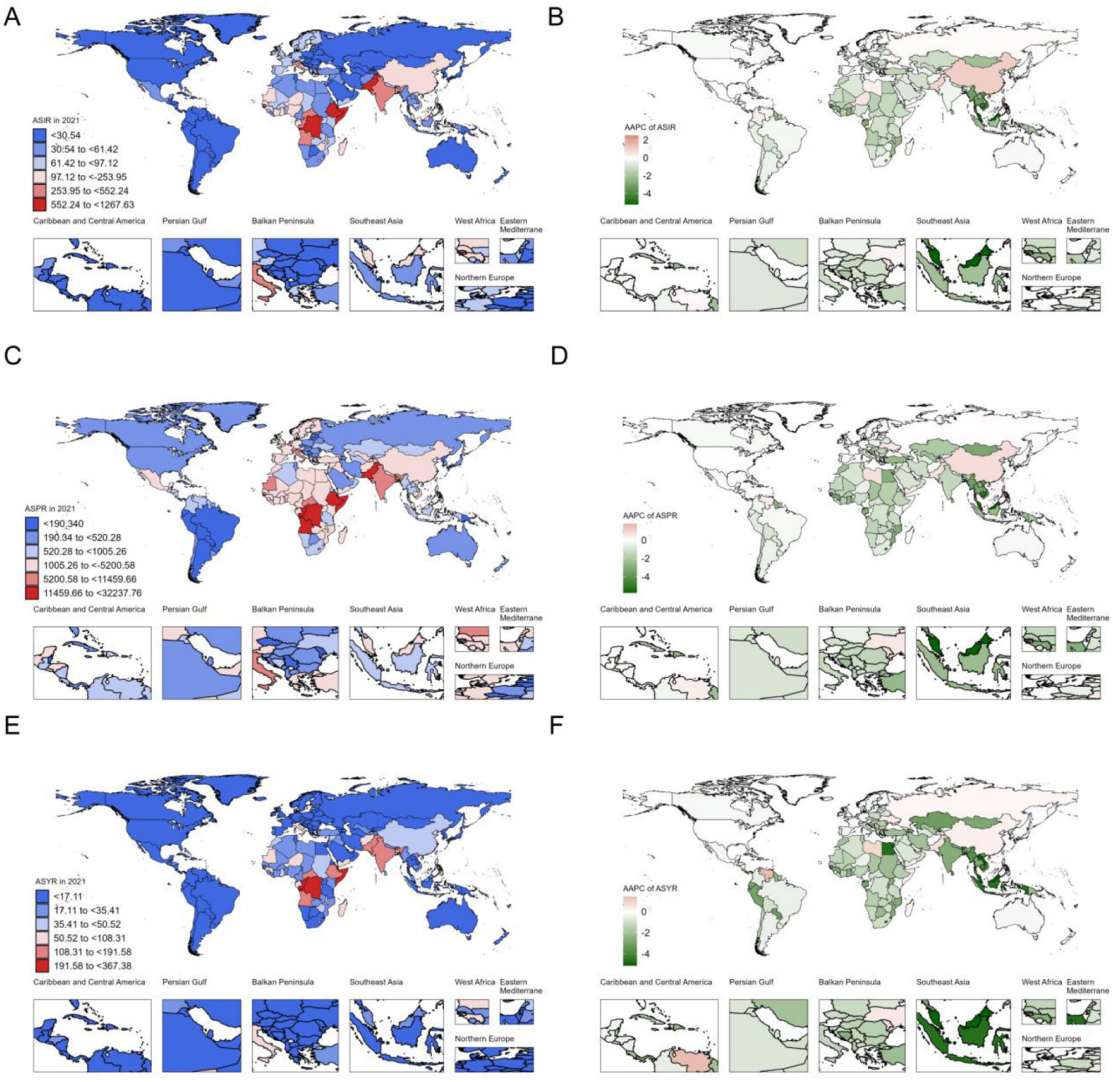
Age-standardized iodine deficiency burden in 2021 and their AAPCs among WRA across 204 countries and territories from 1990 to 2021. **(A)** ASIR in 2021; **(B)** AAPC of ASIR; **(C)** ASPR in 2021; **(D)** AAPC of ASPR; **(E)** ASYR in 2021; **(F)** AAPC of ASYR. AAPC, average annual percent change.

At the country level, 174 countries (85.29%) exhibited a decline in the ASIR of iodine deficiency, 17 countries (8.33%) showed an increase trend, and 13 countries (6.37%) displayed no significant change. Philippines showed the fastest increase in ASIR [from 104.8 (95% CI 65.7, 151.4) in 1990 to 225.5 (95% CI 166.9, 292.4) in 2021, AAPC = 2.51 (95% CI 2.35, 2.67)] and ASPR [from 2,441.4 (95% CI 1,848.9, 3,079.6) in 1990 to 4,237.3 (95% UI: 3,251.3, 5,317.8)] in 2021 [AAPC: 1.79 (95% CI 1.61, 1.97)] (Supplementary Table S1, Figure 4, Supplementary Figure S8). Inferior to Philippines, China also showed significant increases in ASIR [from 120.2 (95% CI 82.5, 166.2) in 1990 to 165.9 (95% CI 123.8, 213.0) in 2021, AAPC = 1.04 (95% CI 0.9, 1.18)], ASPR [from 2,594.1 (95% CI 1,944.6, 3,322.4) in 1990 to 3,303.6 (95% CI 2,537.3, 4,104.1) in 2021, AAPC: 0.78 (95% CI 0.64, 0.92)] and ASYR (AAPC = 0.31; Supplementary Table S1, Figure 4, Supplementary Figure S9). Pakistan also demonstrated notable increases in ASIR (AAPC = 0.9), ASPR (AAPC = 0.7), and ASYR (AAPC = 0.63; Supplementary Table S1, Figure 4, Supplementary Figure S11). Other countries with rising ASIR included Republic of Moldova (AAPC = 0.59), Niger (AAPC = 0.58), Libya (AAPC = 0.34), followed by South Sudan (AAPC = 0.29), Somalia (AAPC = 0.29), and Ukraine (AAPC = 0.21). The highest decrease in ASIR and ASPR of iodine deficiency were both observed in Malaysia, with the AAPCs of −5.24 (95% CI −5.48, −5.01) in ASIR, AAPC of −5.77 (95% CI −6.02, −5.51) in ASPR (Supplementary Table S1, Figure 4). Whereas Lao People's Democratic Republic showed the highest reduction in ASYR (AAPC = −5.13, 95% CI: −5.53, −4.73; Supplementary Table S1, Figure 4). Malaysia also showed the relatively remarkable decline of ASYR (AAPC = −5.10, 95% CI: −5.48, −4.73; Supplementary Table S1, Figure 4).

**Figure 5:**
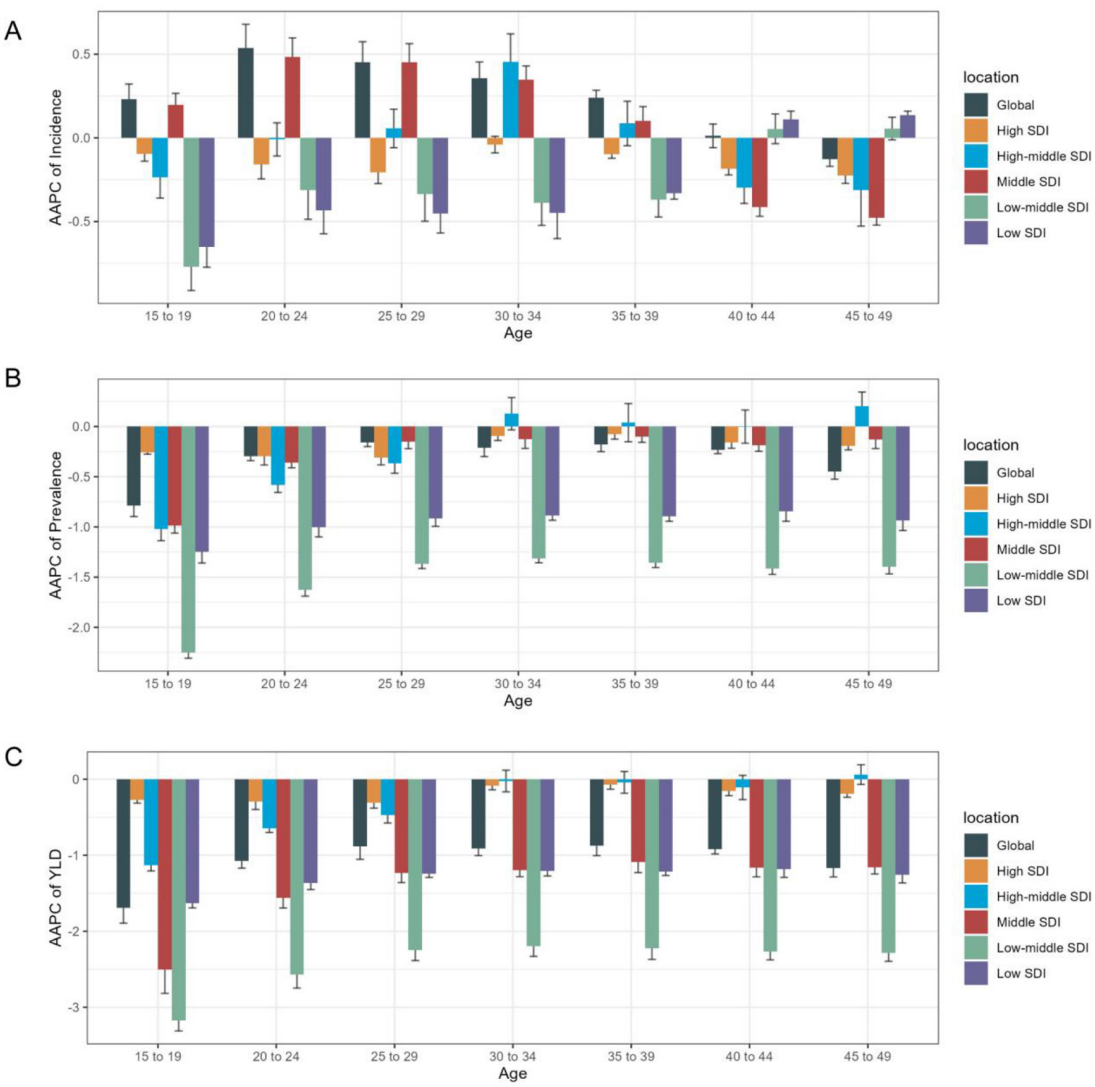
Age distribution of iodine deficiency burden and AAPC of age- specific rate by SDI quintiles from 1990 to 2021. **(A)** AAPC of age specific incidence rate; **(B)** AAPC of age specific prevalence rate; **(C)** AAPC of age specific YLD rate.

When analyzed by age category, individuals aged 20–24 years showed the most significantly upward trend in ASIR [AAPC = 0.54 (95% CI 0.39, 0.68)] (Supplementary Table S2, Figure 5, Supplementary Figure S6). Those aged 15–19 years demonstrated the largest decline in ASPR and ASYR, with AAPC of −0.79 (−0.9, −0.68) and −1.69 (−1.89, −1.49), respectively (Supplementary Table S2, Figure 5).

### The health inequality in iodine deficiency burden among WRA

**Figure 6:**
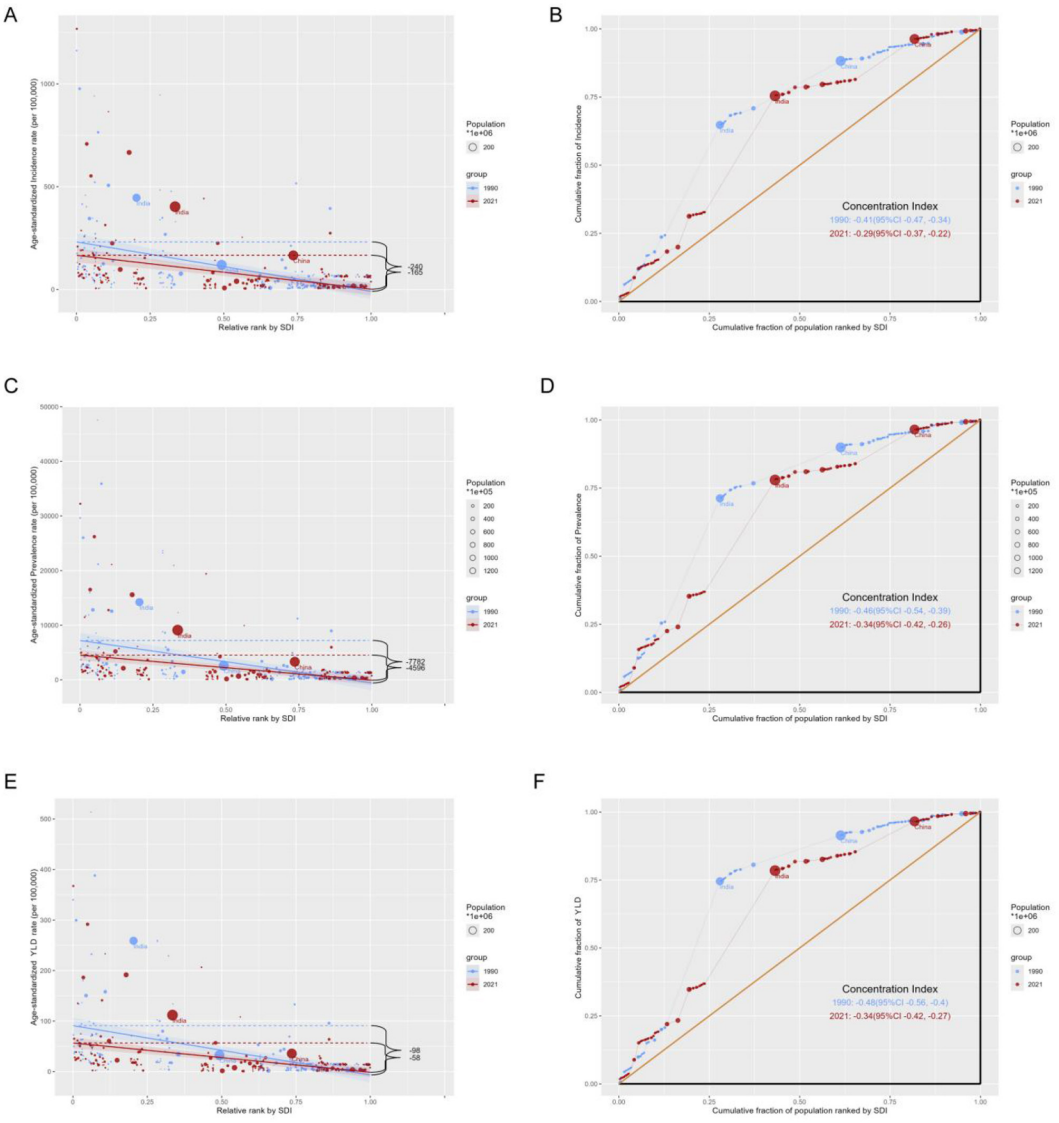
Health inequality regression curves and concentration curves for the age-standardized rates of iodine deficiency among WRA from 1990 to 2021. Age-standardized incidence rate **(A, B)**, prevalence **(C, D)** and disability-adjusted life years **(E, F)** of iodine deficiency. **(A, C, E)** present the slope index of inequality; **(B, D, F)** illustrate the concentration index of inequality. Blue represents data in 1990, and red represents data in 2021. Points representing countries and territories sized by population.

This study identified notable absolute and relative health inequality in the age–standardized incidence, prevalence and YLDs burden of iodine deficiency. The slope index of inequality showed a narrowing gap in the ASIR of iodine deficiency between countries and territories with the highest and lowest SDI, declining from −240.17 (95% CI −305.77, −174.57) in 1990 to −164.73 (95% CI −218.59, −110.88) in 2021 (Figure 6A). Concurrently, the concentration index of ASIR declined from −0.41 (95% CI −0.47, −0.34) in 1990 to −0.29 (95% CI −0.37, −0.22) in 2021 (Figure 6B). The slope index of ASPR declined from −7,782.48 (95% CI −10,084.36, −5,480.60) in 1990 to −4,595.98 (95% CI −6135.01, −3,056.952) in 2021 (Figure 6C), and the concentration index of ASPR decreased from −0.41 (95% CI −0.47, −0.34) in 1990 to −0.29 (95% CI −0.37, −0.22) in 2021 (Figure 6D). The slope index of ASYR decreased from −97.58 (95% CI −122.79, −72.37) in 1990 to −58.03 (95% CI −75.08, −40.99) in 2021 (Figure 6E), with the concentration index of ASYR decreased from −0.48 (95% CI −0.56 to −0.40) in 1990 to −0.34 (95% CI −0.42, −0.27) in 2021 (Figure 6F). These trends collectively reflecting a reduction in the health inequality burden of iodine deficiency over the past three decades (Table 2, Figure 6).

**Table 2:** Health inequalities index of Iodine deficiency burden cross countries from 1990 to 2021.

**Metric**	**Years**	**Slope index (95% CI)**	**Concentration index (95% CI)**
ASIR	1990	−240.17 (−305.77, −174.57)	−0.41 (−0.47 to −0.34)
	2021	−164.73 (−218.59, −110.88)	−0.29 (−0.37 to −0.22)
ASPR	1990	−7,782.48 (−10,084.36 to −5,480.60)	−0.41 (−0.47 to −0.34)
	2021	−4,595.98 (−6,135.01 to 3,056.952)	−0.29 (−0.37 to −0.22)
ASYR	1990	−97.58 (−122.79, −72.37)	−0.48 (−0.56 to −0.40)
	2021	−58.03 (−75.08, −40.99)	−0.34 (−0.42 to −0.27)

ASIR, age-standardized incidence rate; ASPR, age-standardized prevalence rate; ASYR, age-standardized disability adjusted life years rate.

### Projections of iodine deficiency burden to 2044

The global incidence cases of iodine deficiency among reproductive-age women will show an upward trend from 2021 to 2044. The age-standardized incidence rate is projected to remain relatively stable between 2021 and 2030, followed by a slight decline from 2030 to 2044 (Supplementary Figure S12A). Both the age-standardized prevalence rate and age-standardized years lived with disability rate have exhibited a distinct downward trend from 2021 to 2044 (Supplementary Figures S12B,C).

## Discussion

Based on data from the GBD Study 2021, our research first identified a modest but significant elevation in ASIR of iodine deficiency among WRA globally from 1990 to 2021. This elevation was predominantly observed in 8.33% of the countries analyzed. Moreover, the global age-standardized incidence rate is projected to remain relatively stable between 2021 and 2030. The iodine deficiency burden inversely correlated with the SDI, with the highest ASIR observed among low–SDI countries. Specifically, WRA aged 20–24 years demonstrated the highest increase in age specific incidence rate globally. Notably, the health inequality in iodine deficiency burden has diminished over the past 32 years. These findings provide insights into the epidemiological characteristics of iodine deficiency among WRA, highlighting the need for effective interventions and ongoing monitoring to address the issue from a global perspective.

Previous studies have indicated a decline in the global ASIR of iodine deficiency among the general adults and children from 1990 to 2019 (15, 16). Our study corroborates these findings in WRA, with the exception of 8.33% of the countries analyzed. An initial decreasing trend was observed globally and across all SDI categories from 1995 to 2000, consistent with the improvement in the general population globally (15), reflecting the positive effects of USI policies. Between 2000 and 2004, a slight upward trend in ASIR emerged globally, affecting not only middle-SDI countries, but also high–middle and high-SDI countries. The Chinese Ministry of Health reduced salt iodine content from 50 to 35 mg/kg in 2000 in response to excessive iodine levels in the general population. Shifts in industry practices inadvertently reduced the iodine status in certain industrialized countries, such as Australia and several European countries (29–31). Moreover, accumulating evidence has revealed that prolonged exposure to excessive iodine from sustained USI programs may contribute to hypothyroidism, autoimmune thyroiditis (3, 5), and hyperthyroidism (32, 33). Excessive iodine intake could potentially hinder the implementation of iodine prophylaxis strategies. Collectively, these factors may explain the observed upward trends in ASIR and ASPR of iodine deficiency in middle, high–middle and high SDI countries between 2000 and 2005. The upward trend of iodine deficiency during this period highlights the close association between iodine status and iodine-related policies. The reemergence of iodine deficiency underscores the challenge of balancing the elimination of iodine deficiency disorders and maintaining optimal iodine status for thyroid function.

The global ASIR of iodine deficiency has significantly increased since 2010, particularly in low-middle SDI countries. Notably, this upward trend was more pronounced in India (APC_2010 − 2015_ = 4.64) and Pakistan. In this study, the ASIR in India exhibited an upward trend between 2010 and 2015, followed by a decline thereafter. The Indian government has implemented salt iodization legislation since 1992. According to previous reports, the national coverage of iodized salt was 71% in 2009 (34), which was consistent with the observed trends in iodine deficiency documented in this study. The overall ASIR of iodine deficiency in India showed declining (AAPC = −0.33) trend between 1990 and 2021. However, cross-sectional studies have indicated iodine deficiency among pregnant women. Despite the lack of nationally representative UIC data for WRA, this study's findings provide detailed indication of iodine deficiency trends in India. A Pakistani study revealed that the median iodine concentration in salt samples was merely 4.2 mg/kg, substantially below WHO recommended levels. The UIC among WRA was 67 μg/L (35), clearly indicating iodine deficiency. Further efforts should prioritize systematic monitoring of iodine status and iodized salt intake among vulnerable populations, with a particular focus on WRA and pregnant women.

While iodine deficiency among WRA exhibited a declining trend in most countries, 8.33% of the countries demonstrated an upward trend. Notably, the Philippines, China, and Pakistan recorded the highest increases in AAPC.

Philippines was the only one among 41 middle SDI countries to show an upward trend in the ASIR of iodine deficiency from 1990 to 2021. The ASIR in 2021 was approximately 1.15-fold higher than that in 1990 (AAPC = 2.51). Similar increasing trends were observed in the ASPR and ASYR, with AAPCs of 1.79 (a 73.56% increase from 1990) and 0.79 (a 28.09% increase from 1990), respectively. Furthermore, a slight upward trend in ASIR was observed between 2018 and 2021. A cross-sectional study in the Philippines (36) corroborated these findings, revealing that UIC among WRA were below 100 μg/L, indicating iodine deficiency. However, nationally representative data on iodine deficiency among reproductive–age or pregnant women in Philippines remain scarce. As a consequence, the increasing trend in iodine deficiency identified in this study underscores the need to sustain efforts to eliminate iodine deficiency disorders and strengthen systematic monitoring programs, particularly among WRA.

Our study revealed that East Asia exhibited the greatest increases in the ASIR, ASPR, and ASYR among 21 GBD regions. China was the sole country in East Asia to show an upward trend in ASIR from 1990 to 2021, recording the most significant increase in ASIR among 46 high–middle SDI countries, with an AAPC of 1.04 and representing 38.02% increase in ASIR, which is inferior to the Philippines. In China, salt iodization was first implemented in iodine-deficient areas in 1979. Subsequently, a systematic USI policy was implemented nationwide in 1994 in compliance with WHO recommendations, yielding remarkable achievements. However, China's iodine status shift from adequate to excessive between 1997 and 1999 (5), prompting the Chinese Ministry of Health to reduce the salt iodine content in 2000. This study identified a remarkable upward trend in ASIR (APC = 8.39, *P* < 0.05) and ASPR (APC = 7.9, *P* < 0.05) of iodine deficiency among WRA in China between 2000 and 2004, alongside a substantial rise in ASYR (APC = 6.72, *P* < 0.05) between 2000 and 2005. The increase in ASIR during this period may be attributed to the reduced iodized level. Since 2012, China has adjusted its iodized salt standard to 25–30 mg/kg. Our findings show a substantial decline in ASIR, ASPR and ASYR between 2007 and 2010 (ASIR: AAPC = −2.7, ASPR: AAPC = −2.59, and ASYR: AAPC = −2.04), followed by a consistently wild decreasing trend up to now, with no significant impact from the salt iodine reduction in 2012. Despite these declines since 2007, the rates remain higher than those in 1990. In 2015, The China National Nutrition and Health Survey first documented iodine status in pregnancy, revealing mild iodine deficiency in 24.4% of participants (37), consistent with this study. Li et al. (38) estimated approximately 66.18 million cases of iodine deficiency among WRA in China, nearly sixfold higher than our study's estimates. Moreover, subnational studies have identified iodine deficiency in pregnancy across various regions in China (39, 40). Collectively, these trends highlight the urgent need for targeted policies and enhanced surveillance among WRA.

Although only 8.33% of countries exhibited an upward trend in the ASIR of iodine deficiency among WRA, the burden remained significant in specific countries. This study identified the highest burden of iodine deficiency among WRA in Central Sub-Saharan Africa across 21 GBD regions. In 2021, The ASIR in Central Sub-Saharan Africa was twice the global average, while the ASPR and ASYR were fivefold higher than global level. Moreover, a study in the Democratic Republic of Congo revealed that approximately 36.3% of pregnant women were exposed to household salt samples with insufficient iodine content (< 15 ppm) (41). The primary challenges in addressing iodine deficiency in these regions include low socioeconomic status, inadequate educational attainment, insufficient political commitment, and poor supervision of the salt–trade chain. Despite the challenges in managing iodine deficiency in Sub–Saharan Africa and South Asia, strengthening the implementation of USI policies remains the primary strategic approach.

Among 46 high-middle SDI countries, the highest burden of iodine deficiency among WRA was observed in Italy. The ASIR, ASPR, and ASYR of iodine deficiency in Italy were more than fourfold those in Spain, a Western European country with comparable SDI values. This finding was consistent with results reported in published studies in Italy. One study reported a UIC of 74 μg/L during pregnancy in 2006 (42), while another observed a UIC of 110 μg/L in 2019 (43). Notably, iodine status in school-age children were classified as adequate (44), reflecting the effectiveness of Italy's national iodine prophylaxis policy implemented since 2005, to a certain extent. However, our study estimates that the iodine deficiency burden among Italian WRA is more severe than that in most middle, low–middle, and even low SDI countries. These findings underscore the urgent need for policymakers to prioritize iodine deficiency in WRA and pregnant women in Italy. Strategies to address this issue should include: increasing the market share of iodized salt, enhancing prevention awareness of iodine deficiency among fertile women, and strengthening health education initiatives delivered by medical professionals.

Health inequality provides a more comprehensive evaluation of population health shifts compared to merely the average of health indicators (25). This study examined both absolute and relative inequalities in iodine deficiency among WRA from 1990 to 2021. Findings were consistent with the relation between SDI and iodine deficiency burden, highlighting a markedly disproportionate burden in low-SDI countries. Nonetheless, the study confirmed a significant narrowing of health inequality in iodine deficiency between high and low-SDI countries, a trend consistent with the reduction observed among children (22). Health disparities related to iodine deficiency in certain underdeveloped countries can be attributed to the absence, weakness or deferred iodized salt legislation, compounded by factors such as conflict, political instability, economic weakness, inadequate medical resources, and insufficient healthcare and educational infrastructure. Overall, USI has been identified as the most cost-effective strategy for controlling iodine deficiency, owing to its safety, efficacy, affordability and monitoring feasibility (7). Consequently, the sustained and long–term implementation of USI, in collaboration with international agencies to secure expertise, medical assistance and funding, has the potential to reduce health disparities and improve iodine status among WRA.

Pregnant women require more iodine than the general population due to heightened fetal thyroid hormone synthesis and increased maternal renal clearance. Iodine deficiency in pregnancy has been associated with impaired fetal growth and neurodevelopmental deficits in offspring. While the adverse effects of severe iodine deficiency during gestation are well-established (45), the consequences of mild-to-moderate deficiency remain a subject of debate.

An observational study from the ALSPAC cohort revealed that mild-to-moderate iodine deficiency in early pregnancy adversely affected child cognitive development, even in a population with mild iodine deficiency (46). Similarly, the Norwegian Mother and Child Cohort Study found that maternal iodine intake below 160 μg/day was linked to poorer neurodevelopmental outcomes in children at age three (47). A longitudinal follow-up study further corroborated these findings, demonstrating that even mild gestational iodine deficiency (UIC < 150 μg/L) could lead to persistent impairments in fetal neurocognition—effects that were not reversed by achieving iodine sufficiency later in childhood (48). In contrast, a cross-sectional study of 7,190 pregnant women reported that mild iodine deficiency (UIC 100–149 μg/L) in early pregnancy did not elevate the risk of gestational thyroid dysfunction (49). Additionally, iodine supplementation in mildly iodine-deficient pregnant women had no measurable impact on offspring neurodevelopment at 5–6 years of age (50). Prior research had implied the intriguing hypotheses: initiating iodine intervention earlier and ensuring adequate iodine status before conception in WRA, may benefit fetal neurological development, which is consistent with the results emphasized in this study.

The limitations of using Grade 2 goiter as an indicator of iodine deficiency in this study should be carefully considered when interpreting the results. Thyroid size reflects long-term iodine nutrition status (accumulated over months to years), and changes in goiter prevalence do not immediately correspond with the current iodine deficiency status (1). Furthermore, factors such as genetics and environmental influences, including endocrine disruptors, may also predispose to goiter. Consequently, relying on visible Grade 2 goiter as an indicator may result in either underestimation or overestimation of the actual iodine deficiency burden among WRA. UIC is currently the most practical biochemical marker for iodine nutrition (7, 8). Future research should prioritize more extensive and reliable UIC-based data among WRA. Sustained monitoring of UIC, particularly to assess varying severity levels of iodine deficiency, is crucial for ensuring adequate iodine nutrition in both WRA and pregnant women. Such surveillance should be emphasized in public health policies.

## Conclusion

The incidence of iodine deficiency among women of reproductive age increased significantly globally from 1990 to 2021, with the highest burden observed in low-middle SDI and low SDI quintile countries. Health inequalities in iodine deficiency remained pronounced across countries. The data emphasize the necessity of routinely iodine status monitoring in WRA. Such monitoring, combined with assessments of thyroid function in WRA, should be conducted routinely, and iodine supplementation can be administered when indicated.

## Data Availability

Publicly available datasets were analyzed in this study. This data can be found here: https://ghdx.healthdata.org/gbd-2021/sources.
