# Supplementary material for: Global, regional, and national burden of iodine deficiency for women of reproductive age, 1990–2021: a systematic analysis based on the Global Burden of Disease Study 2021
Source: Front Nutr. 2025 Sep 18;12:1577169. doi: 10.3389/fnut.2025.1577169 (PMC12488439; doi:10.3389/fnut.2025.1577169)
Supplement: Supplementary file 1 [file Supplementary_file_1.docx]

**Supplementary materials**

**Global, regional, and national burden of iodine deficiency for women of** **reproductive age, 1990–2021: A systematic analysis based on the Global Burden of Disease Study 2021**

**Additional Results in Tables and Figure**

[**Supplementary Table 1**. The iodine deficiency burden for reproductive-age women from 1990 to 2021 by nations. 3](#_Toc22430)

[**Supplementary Table 2** Age-specific AAPC of iodine deficiency burden from 1990 to 2021 globally and by SDI categories. 44](#_Toc28953)

[**Supplementary Figure 1.** The Proportion of cases in different age groups for iodine deficiency burden globally and by SDI categories from 1990 to 2021. 48](#_Toc16991)

Supplementary Figure 2. The incidence number of iodine deficiency among WRA in different age groups globally and by SDI categories from 1990 to 2021. ……………………………………………………………………………….. 49

[**Supplementary Figure 3.** The age specific rate of iodine deficiency burden among WRA globally and by SDI quintiles from 1990 to 2021**…………….** 50](#_Toc29514)

[**Supplementary Figure 4.** Joinpoint regression analysis of temporal trends for age-standardized prevalence rate of iodine deficiency among WRA globally and by SDI quintiles from 1990 to 2021. 51](#_Toc2884)

[**Supplementary Figure 5.** Joinpoint regression analysis of temporal trends for age-standardized YLD rate of iodine deficiency among WRA globally and by SDI quintiles from 1990 to 2021 52](#_Toc17422)

[**Supplementary Figure 6.** Joinpoint regression analysis of temporal trends of age-specific incidence rate for iodine deficiency globally by ages 1990 to 2021. 53](#_Toc11040)

[**Supplementary Figure 7.** Age-standardized disease burden of iodine deficiency for reproductive-age women across 204 countries and territories in 1990. 54](#_Toc31052)

Supplementary Figure 8. Temporal trend of iodine deficiency burden in women of reproductive age in middle SDI country (Philippines) from 1990 to 2021………………………………………………………………………………….55

[**Supplementary Figure 9.** Temporal trend of iodine deficiency burden in women of reproductive age in High-middle SDI country (China) from 1990 to 2021. 56](#_Toc28018)

[**Supplementary Figure 10.** Temporal trend of iodine deficiency burden in women of reproductive age in India of low-middle SDI country from 1990 to 2021. 57](#_Toc27365)

[**Supplementary Figure 11.** Temporal trend of iodine deficiency burden in women of reproductive age in Pakistan of low-middle SDI country from 1990 to 2021. 58](#_Toc26934)

[**Supplementary Figure 12.** The projections of global iodine deficiency burden among reproductive- age women burden from 2021 to 2044.. 59](#_Toc26934)

# Supplementary Table 1. The iodine deficiency burden for reproductive-age women from 1990 to 2021 by nations.

| **Location** | **Incidence number 1990 (95% UI)** | **ASIR 1990 (95% CI)** | **Incidence number 2021  (95% UI)** | **ASIR 2021 (95% CI)** | **AAPC (95% CI)** | ***P*** |
| --- | --- | --- | --- | --- | --- | --- |
| Afghanistan | 1458.3 (840.7 to 2280.6) | 61.1 (34.1 to 97) | 4181.4 (2350.2 to 7003.5) | 56 (30.5 to 95) | -0.29(-0.35 to -0.23) | <0.001 |
| Albania | 181.9 (99.7 to 289) | 20.7 (11.2 to 33.2) | 81.4 (44.5 to 128.4) | 13.6 (7.5 to 21.4) | -1.36(-1.43 to -1.3) | <0.001 |
| Algeria | 2295.4 (1374.5 to 3533.5) | 38.7(23 to 59.8) | 3484.2 (1994.4 to 5497.9) | 31.1 (17.9 to 49.2) | -0.71(-0.79 to -0.63) | <0.001 |
| American Samoa | 0.7 (0.4 to 1) | 5.2 (2.9 to 8.1) | 0.5 (0.2 to 0.7) | 4 (2.1 to 6.3) | -0.9(-0.92 to -0.88) | <0.001 |
| Andorra | 8.7 (5.2 to 13) | 59.3 (35.6 to 89) | 10.4 (6.2 to 15.5) | 61.9 (38.3 to 90.4) | 0.14(0.11 to 0.17) | <0.001 |
| Angola | 14322.1 (9773.9 to 20012.5) | 521.4 (353 to 732.6) | 28080 (18200.5 to 40033.7) | 313.5 (200.6 to 451.1) | -1.64(-1.78 to -1.51) | <0.001 |
| Antigua and Barbuda | 3.9 (2.3 to 5.9) | 23.1 (13.4 to 35.1) | 2.8 (1.5 to 4.3) | 11.9 (6.7 to 18.2) | -2.13(-2.2 to -2.06) | <0.001 |
| Argentina | 700.5 (385.5 to 1127.4) | 8.7 (4.7 to 14) | 882.2 (477.8 to 1421.8) | 7.5 (4.1 to 12.1) | -0.44(-0.45 to -0.42) | <0.001 |
| Armenia | 383 (225.6 to 610.8) | 43.7 (26 to 69.4) | 248.8 (134.7 to 414.7) | 39.5 (22.2 to 64.1) | -0.52(-1.07 to 0.02) | 0.06 |
| Australia | 660.4 (360.6 to 1044.5) | 14.9 (8.2 to 23.6) | 818.1 (446.8 to 1284.1) | 14 (7.7 to 21.9) | -0.18(-0.28 to -0.08) | 0.001 |
| Austria | 1240.3 (771.3 to 1840) | 64.1 (40 to 94.8) | 1134.2 (688 to 1705) | 63.6 (38.9 to 94.7) | -0.02(-0.06 to 0.02) | 0.36 |
| Azerbaijan | 379 (225.1 to 575.6) | 19.1 (11.3 to 29.2) | 407.1 (233.9 to 620.8) | 15.8 (9.2 to 23.8) | -0.63(-0.72 to -0.54) | <0.001 |
| Bahamas | 12 (7.1 to 17.9) | 15.8 (9.3 to 23.8) | 11.4 (6.4 to 17.7) | 10.7 (6 to 16.6) | -1.26(-1.29 to -1.23) | <0.001 |
| Bahrain | 33.2 (19.7 to 50.5) | 29.2 (17.3 to 44.3) | 76.4 (45 to 117.5) | 23.6 (13.9 to 36.1) | -0.68(-0.72 to -0.64) | <0.001 |
| Bangladesh | 105023 (84631.7 to 129009) | 345.2 (275.9 to 427.7) | 107072.8 (74935.8 to 150387.9) | 224.8 (157.3 to 316.1) | -1.46(-1.71 to -1.2) | <0.001 |
| Barbados | 23.1 (13.3 to 35.2) | 33 .0(18.9 to 50.4) | 18.4 (9.5 to 30.3) | 27.7 (14.4 to 45.2) | -0.56(-0.6 to -0.53) | <0.001 |
| Belarus | 237.7 (123.5 to 392.6) | 9.6(5.0 to 15.8) | 159.6 (84 to 265.8) | 8.2 (4.4 to 13.4) | -0.54(-0.6 to -0.47) | <0.001 |
| Belgium | 1513.1 (944.9 to 2251.4) | 64.3(40.3 to 95.2) | 1415.9 (865.4 to 2129.8) | 62.2 (38.3 to 92.8) | -0.11(-0.12 to -0.1) | <0.001 |
| Belize | 5.2 (3.1 to 7.8) | 11.4(6.8 to 17.1) | 9 (5.2 to 13.7) | 7.3 (4.2 to 11.2) | -1.44(-1.5 to -1.39) | <0.001 |
| Benin | 1098 (662.8 to 1618) | 90 (53.9 to 134.5) | 2591.6 (1531.6 to 3882.9) | 73 (42.9 to 110.8) | -0.68(-0.76 to -0.6) | <0.001 |
| Bermuda | 2.3 (1.4 to 3.5) | 14 (8.3 to 21) | 1 (0.6 to 1.6) | 8.1 (4.6 to 12.5) | -1.75(-1.83 to -1.67) | <0.001 |
| Bhutan | 168.5(114.1 to 241.9) | 98.4 (66.1 to 142.4) | 124.9 (80.6 to 179) | 59.1 (38.1 to 84.9) | -1.6(-1.77 to -1.44) | <0.001 |
| Bolivia (Plurinational State of) | 120.4 (67.8 to 186.7) | 7.6 (4.2 to 11.9) | 179.6 (94.7 to 292.8) | 5.7 (3 to 9.4) | -0.91(-0.94 to -0.88) | <0.001 |
| Bosnia and Herzegovina | 546.6 (259.5 to 957.7) | 46.6 (22.2 to 81.7) | 177.9 (90.5 to 296.5) | 26.8 (13.9 to 43.9) | -1.79(-1.93 to -1.64) | <0.001 |
| Botswana | 99.1 (60.4 to 145.6) | 29 (17.5 to 42.7) | 160.2 (96.4 to 235) | 23.5 (14.1 to 34.5) | -0.67(-0.7 to -0.64) | <0.001 |
| Brazil | 3217.2 (1775.8 to 5092.5) | 8 (4.4 to 12.8) | 4268.2 (2289 to 6815) | 7.5 (4 to 11.8) | -0.23(-0.26 to -0.21) | <0.001 |
| Brunei Darussalam | 11.9 (6.7 to 18) | 17.1 (9.6 to 26.2) | 20.2 (11.1 to 32) | 16.5 (9.1 to 26.1) | -0.12(-0.15 to -0.1) | <0.001 |
| Bulgaria | 389.6 (213.2 to 615.7) | 19.5 (10.7 to 30.6) | 187 (100.6 to 302.5) | 14.3 (7.7 to 22.8) | -1.02(-1.06 to -0.99) | <0.001 |
| Burkina Faso | 2582.4 (1591.2 to 3807.6) | 110.5 (67.5 to 164.5) | 4177.9 (2571 to 6296.8) | 69.6 (42.4 to 106.4) | -1.5(-1.58 to -1.41) | <0.001 |
| Burundi | 3864 (2985.2 to 4947.1) | 263.3 (201.3 to 341.3) | 6886.5 (4468.7 to 9800.3) | 190.5 (122.6 to 273.4) | -1.07(-1.18 to -0.96) | <0.001 |
| Cabo Verde | 127.5 (80.4 to 181.3) | 135.6 (84.8 to 195.6) | 148.7 (89.7 to 220.7) | 96.8 (58.1 to 144.4) | -1.08(-1.23 to -0.92) | <0.001 |
| Cambodia | 9614.5 (7177.3 to 12559.1) | 333.0 (248.1 to 436.3) | 3578.2 (2479 to 5000.8) | 77.3 (53.4 to 108.3) | -4.64(-4.76 to -4.52) | <0.001 |
| Cameroon | 1497.5 (919.4 to 2226.4) | 57.6 (35 to 86.2) | 4736.3 (2832.1 to 7077.4) | 56.5 (33.5 to 85.4) | -0.07(-0.13 to -0.01) | 0.025 |
| Canada | 1152.5 (637 to 1823.8) | 16 .0(8.9 to 25.2) | 1218.6 (674 to 1934.5) | 15.2 (8.5 to 24) | -0.15(-0.16 to -0.14) | <0.001 |
| Central African Republic | 3061.1 (2307 to 3881.1) | 403.0 (302 to 514) | 4598 (3066.2 to 6543.7) | 286.8 (189.3 to 411.6) | -1.08(-1.33 to -0.84) | <0.001 |
| Chad | 1975.1 (1188.6 to 2893.4) | 130.4 (77.6 to 193.5) | 3519.2 (2083.6 to 5352.2) | 81.1 (47.9 to 124.8) | -1.52(-1.61 to -1.43) | <0.001 |
| Chile | 345.1 (197.1 to 547) | 9.4 (5.3 to 14.9) | 345.6 (186.1 to 561.2) | 7.6 (4.1 to 12.2) | -0.7(-0.71 to -0.68) | <0.001 |
| China | 430210.1 (295983.7 to 592036.6) | 120.2 (82.5 to 166.2) | 428508 (318682 to 553773.6) | 165.9 (123.8 to 213) | 1.04(0.9 to 1.18) | <0.001 |
| Colombia | 2442.3 (1357.7 to 3920.4) | 26.6(14.7 to 43) | 3066.5 (1706.3 to 4836.6) | 23.8 (13.4 to 37.3) | -0.35(-0.38 to -0.33) | <0.001 |
| Comoros | 37.6 (23.8 to 53.9) | 33 (20.7 to 47.8) | 69.5 (42.4 to 101.3) | 34.7 (21.1 to 50.7) | 0.16(0.13 to 0.19) | <0.001 |
| Congo | 3356.8 (2323.1 to 4568.1) | 477.5 (326.6 to 654.7) | 6812.3 (4392.1 to 9840.7) | 442 (282.8 to 641.7) | -0.25(-0.4 to -0.1) | 0.001 |
| Cook Islands | 0.3 (0.2 to 0.4) | 6.1 (3.3 to 9.5) | 0.2 (0.1 to 0.3) | 4.2 (2.2 to 6.7) | -1.13(-1.16 to -1.11) | <0.001 |
| Costa Rica | 206.7 (115.3 to 326) | 25.4 (14.1 to 40.3) | 280.2 (152.8 to 448.9) | 22.4 (12.3 to 35.6) | -0.41(-0.42 to -0.39) | <0.001 |
| Coted'Ivoire | 5414.4 (3428.6 to 7704.4) | 166.7 (105 to 239.6) | 7666.5 (4764.2 to 11326) | 106.3 (65.6 to 158.4) | -1.47(-1.61 to -1.34) | <0.001 |
| Croatia | 197.8 (108.5 to 306.9) | 17 (9.4 to 26.2) | 110.6 (58.8 to 175.1) | 13.1 (7 to 20.5) | -0.85(-0.89 to -0.81) | <0.001 |
| Cuba | 1066.9 (604.9 to 1662.4) | 33.1 (18.8 to 51.7) | 500.7 (266.5 to 804.5) | 21.5 (11.4 to 34.3) | -1.4(-1.55 to -1.26) | <0.001 |
| Cyprus | 132.1 (82.1 to 194) | 67.9 (42.3 to 99.6) | 205.8 (126.6 to 307.8) | 66.8 (41.5 to 98.3) | -0.05(-0.07 to -0.03) | <0.001 |
| Czechia | 278.4 (160.9 to 438.1) | 11.0(6.4 to 17.3) | 184.5 (103.6 to 287.1) | 8.6 (4.9 to 13.3) | -0.8(-0.82 to -0.78) | <0.001 |
| Democratic People's  Republic of Korea | 526.9 (319 to 782.5) | 9.1 (5.5 to 13.6) | 403.9 (230.4 to 614) | 6.2 (3.5 to 9.5) | -1.22(-1.26 to -1.18) | <0.001 |
| Democratic Republic of the Congo | 79421.8 (65037.5 to 96742.9) | 764.6 (623.4 to 934.6) | 140586.8 (84064.7 to 208576.1) | 552.2 (324.8 to 824.3) | -1.04(-1.14 to -0.94) | <0.001 |
| Denmark | 751.4 (460.1 to 1128.3) | 60.6 (37.4 to 90.2) | 691.7 (429 to 1021) | 58 (36.2 to 85) | -0.14(-0.17 to -0.12) | <0.001 |
| Djibouti | 1147.8 (845.6 to 1469.8) | 946.2 (698.9 to 1208.9) | 2754.7 (2052 to 3567.8) | 864.8 (643.6 to 1121.3) | -0.3(-0.35 to -0.25) | <0.001 |
| Dominica | 6.9 (4 to 10.3) | 36.4 (20.8 to 55.4) | 3.1 (1.7 to 4.8) | 18.9 (10.3 to 29.2) | -2.1(-2.17 to -2.02) | <0.001 |
| Dominican Republic | 972.9 (539.7 to 1461.1) | 45.4 (25.1 to 69) | 667.4 (356.4 to 1051.8) | 22.8 (12.2 to 36) | -2.2(-2.23 to -2.17) | <0.001 |
| Ecuador | 155.2 (83.5 to 252.6) | 6.0 (3.2 to 9.8) | 245.1 (130.7 to 398.4) | 5.2 (2.8 to 8.4) | -0.45(-0.47 to -0.42) | <0.001 |
| Egypt | 9540.2 (5597.5 to 15595.1) | 69.3 (39.9 to 114.1) | 11274 (6340.3 to 17886.4) | 43.3 (24.3 to 68.7) | -1.53(-1.69 to -1.38) | <0.001 |
| El Salvador | 449.9 (252 to 716.8) | 31.2 (17.2 to 50.1) | 454 (253.2 to 708.8) | 25.5 (14.4 to 39.7) | -0.64(-0.66 to -0.62) | <0.001 |
| Equatorial Guinea | 1113.9 (716.2 to 1543.1) | 941 (601.6 to 1304.9) | 1053.6 (700.9 to 1483.2) | 253.9 (167.8 to 360) | -4.15(-4.38 to -3.92) | <0.001 |
| Eritrea | 528.9 (334 to 767.2) | 61.5(38.4 to 90.1) | 722.7 (450.3 to 1055.7) | 41.7 (25.9 to 61.3) | -1.26(-1.33 to -1.18) | <0.001 |
| Estonia | 26.2 (13.9 to 43.5) | 7.1 (3.8 to 11.7) | 15.8 (8.5 to 25.9) | 6 (3.3 to 9.8) | -0.54(-0.64 to -0.44) | <0.001 |
| Eswatini | 169.5 (103.1 to 252.6) | 78.1 (47.1 to 117.5) | 207.6 (122.9 to 319) | 62.9 (37 to 97.1) | -0.7(-0.78 to -0.61) | <0.001 |
| Ethiopia | 132941.7 (99830.2 to 170647.8) | 976.3 (734.1 to 1252.9) | 234037.5 (175264.9 to 300972.9) | 708.1 (530.2 to 910.8) | -1.08(-1.29 to -0.87) | <0.001 |
| Fiji | 15.6 (8.4 to 24.3) | 7.7 (4.1 to 12.1) | 11.1 (5.8 to 17.9) | 4.9 (2.5 to 7.8) | -1.47(-1.52 to -1.43) | <0.001 |
| Finland | 755.1 (461.7 to 1134.1) | 65.4 (40.3 to 97) | 666.7 (411.3 to 997.8) | 63.4 (39.4 to 94.2) | -0.09(-0.12 to -0.06) | <0.001 |
| France | 9008.6 (5669.1 to 13363.3) | 63.8 (40.3 to 94.3) | 8451.5 (5139.7 to 12473.1) | 64.2 (39.3 to 93.9) | 0.02(0 to 0.03) | 0.049 |
| Gabon | 896.8 (623.9 to 1210.9) | 330.0 (227.6 to 449.3) | 764.5 (507.7 to 1063.8) | 141.7 (93.3 to 198.8) | -2.71(-2.86 to -2.56) | <0.001 |
| Gambia | 959.9 (652.7 to 1289.8) | 346.6 (234.9 to 468.7) | 1791.2 (1160.7 to 2517.3) | 249.4 (161.1 to 353.3) | -1.09(-1.2 to -0.98) | <0.001 |
| Georgia | 287.5 (167 to 444.8) | 21.2 (12.4 to 32.8) | 141.7 (79 to 224.1) | 19.7 (11.2 to 30.7) | -0.25(-0.28 to -0.22) | <0.001 |
| Germany | 11842.5 (7337.4 to 17467.2) | 63.5(39.5 to 93.2) | 9720 (5985.6 to 14372.7) | 63 (39.2 to 92.2) | -0.03(-0.05 to 0) | 0.087 |
| Ghana | 14388.2 (9799.8 to 19585.4) | 348.6(236.7 to 476.7) | 15085.1 (9410.1 to 21602.5) | 153.7 (95.5 to 221.4) | -2.62(-2.69 to -2.56) | <0.001 |
| Greece | 1849.3 (1116.2 to 2745.5) | 75.4 (45.7 to 111.5) | 1361.5 (821.4 to 2078.7) | 73.8 (45.3 to 110.5) | -0.06(-0.13 to 0.02) | 0.135 |
| Greenland | 2.2 (1.2 to 3.5) | 15.0 (8.2 to 23.7) | 1.9 (1 to 3) | 15 (8.2 to 23.6) | 0(-0.02 to 0.01) | 0.68 |
| Grenada | 8.1 (4.6 to 12.7) | 37.6 (21.2 to 59.4) | 4 (2.2 to 6.2) | 15.7 (8.6 to 24.3) | -2.78(-2.86 to -2.7) | <0.001 |
| Guam | 1.9 (1 to 2.8) | 5.3 (2.9 to 8) | 1.5 (0.8 to 2.4) | 4.2 (2.2 to 6.7) | -0.75(-0.79 to -0.71) | <0.001 |
| Guatemala | 526.2 (301 to 832) | 26.3 (14.7 to 42.1) | 1160.1 (645.1 to 1830.7) | 25.2 (14 to 39.9) | -0.14(-0.21 to -0.07) | <0.001 |
| Guinea | 6330 (3782.8 to 9165.1) | 415.5 (247.7 to 602.8) | 7249.5 (4680.2 to 10523.1) | 191.9 (123.2 to 281) | -2.46(-2.56 to -2.36) | <0.001 |
| Guinea-Bissau | 1267.2 (870.6 to 1717.6) | 458.5 (314.1 to 623.1) | 1530 (1030.8 to 2105) | 257.2 (172.5 to 355.9) | -1.83(-1.9 to -1.75) | <0.001 |
| Guyana | 130.1 (73.7 to 194.4) | 55.9 (31.5 to 84.6) | 61.3 (32.1 to 96.1) | 29.4 (15.5 to 46.2) | -2.07(-2.18 to -1.95) | <0.001 |
| Haiti | 1382.4 (836.4 to 2049.5) | 79.7(47.8 to 119.1) | 2539.2 (1364.7 to 3877.4) | 69.2 (37.1 to 106.1) | -0.48(-0.63 to -0.34) | <0.001 |
| Honduras | 391.1 (217 to 629.8) | 33.2 (18 to 54.4) | 892.2 (488.9 to 1435.3) | 29.9 (16.3 to 48.3) | -0.34(-0.39 to -0.29) | <0.001 |
| Hungary | 460.9 (252.2 to 737.7) | 18.9 (10.4 to 30.2) | 261.4 (142.2 to 419.3) | 13.2 (7.2 to 20.7) | -1.18(-1.22 to -1.14) | <0.001 |
| Iceland | 16.5 (10.2 to 24) | 25.2 (15.6 to 36.8) | 18.9 (11.7 to 27.9) | 24.6 (15.3 to 36.1) | -0.05(-0.09 to -0.02) | 0.006 |
| India | 1005404.3 (693382.9 to 1365621) | 446.1 (306.8 to 607.3) | 1567896.2 (1151139.1 to 2053295.6) | 402.7 (295.6 to 527.7) | -0.33(-0.69 to 0.02) | 0.066 |
| Indonesia | 40907.4 (27348.9 to 58121) | 77.1 (51.2 to 110.3) | 29266.7 (19051.4 to 42127.2) | 40.2 (26.3 to 57.6) | -2.07(-2.12 to -2.03) | <0.001 |
| Iran (Islamic Republic of) | 3204.1 (1876.3 to 4907.3) | 24.7(14.4 to 38) | 4215.7 (2388.3 to 6506.5) | 18.2 (10.4 to 28.1) | -0.98(-1.05 to -0.92) | <0.001 |
| Iraq | 2484.6 (1426.9 to 3999.7) | 58.3 (32.8 to 94.6) | 5120.9 (2833.3 to 8285.3) | 48.2 (26.5 to 78.2) | -0.63(-0.74 to -0.51) | <0.001 |
| Ireland | 611.9 (372.6 to 922.1) | 68.3 (41.5 to 103.1) | 650.5 (392.8 to 986.1) | 62.1 (38 to 92.6) | -0.3(-0.35 to -0.26) | <0.001 |
| Israel | 847.3 (523.3 to 1258.3) | 68.1 (42 to 101.3) | 1414.3 (860.4 to 2124.5) | 65.2 (39.8 to 97.6) | -0.13(-0.16 to -0.11) | <0.001 |
| Italy | 54584.1 (40215.3 to 70155) | 394.1 (290.8 to 505.7) | 26413.2 (18812.3 to 35296) | 274 (196.7 to 361.8) | -1.16(-1.27 to -1.05) | <0.001 |
| Jamaica | 241.2 (137.2 to 369.5) | 36.2 (20.4 to 55.9) | 167.5 (89.6 to 263.8) | 21.7 (11.7 to 34.2) | -1.62(-1.78 to -1.47) | <0.001 |
| Japan | 6128.9 (3460.3 to 9488.9) | 19.6 (11.2 to 30.2) | 4238.8 (2367.9 to 6591.2) | 18.1 (10.2 to 27.9) | -0.24(-0.29 to -0.2) | <0.001 |
| Jordan | 307.3 (184.4 to 474.6) | 35.8 (21.2 to 55.4) | 917.7 (540.4 to 1409.7) | 29.4 (17.3 to 45.3) | -0.65(-0.69 to -0.6) | <0.001 |
| Kazakhstan | 1318.3 (778.5 to 2077) | 31.2 (18.3 to 49.3) | 1002.5 (567.3 to 1589.5) | 22.4 (12.7 to 35.4) | -1.1(-1.27 to -0.94) | <0.001 |
| Kenya | 4636.5 (2918.8 to 6686.6) | 78.4 (48.7 to 114.7) | 11581.4 (7167.1 to 16823) | 80.2 (49.3 to 117.4) | 0.08(0.02 to 0.14) | 0.015 |
| Kiribati | 2.2 (1.1 to 3.4) | 11.1 (5.8 to 17.5) | 2.3 (1.2 to 3.7) | 7.1 (3.7 to 11.4) | -1.48(-1.59 to -1.37) | <0.001 |
| Kuwait | 113.6 (66.8 to 172) | 27.7 (16.4 to 41.8) | 332.1 (194.9 to 508.4) | 22.7 (13.5 to 34.8) | -0.64(-0.66 to -0.62) | <0.001 |
| Kyrgyzstan | 202.1 (123.6 to 300.8) | 17.9 (10.8 to 26.9) | 300.8 (173 to 465.3) | 17.5 (10.1 to 27.1) | -0.08(-0.14 to -0.03) | 0.002 |
| Lao People's Democratic Republic | 865.9 (590.4 to 1188.7) | 79.3 (53.7 to 109.8) | 899.2 (600.3 to 1282.4) | 43.4 (28.8 to 62.2) | -1.94(-1.97 to -1.9) | <0.001 |
| Latvia | 43.4 (23.3 to 70.2) | 6.9 (3.7 to 11.1) | 23.2 (12.6 to 38) | 6.3 (3.5 to 10.3) | -0.31(-0.43 to -0.19) | <0.001 |
| Lebanon | 470.9 (310.8 to 706.2) | 60.7 (39.5 to 91.6) | 760.5 (412.6 to 1238.3) | 52.3 (28.7 to 85) | -0.47(-0.5 to -0.44) | <0.001 |
| Lesotho | 2079.7 (1639.6 to 2548.3) | 483.7 (377.9 to 596.9) | 1376.6 (859.4 to 1959.1) | 242.3 (150.8 to 347.1) | -2.28(-2.51 to -2.05) | <0.001 |
| Liberia | 349.6 (218.2 to 512.6) | 57.6 (35.5 to 85.3) | 815.3 (484.5 to 1218.8) | 55.5 (32.9 to 83.5) | -0.17(-0.34 to 0) | 0.055 |
| Libya | 415.3 (246.5 to 640.9) | 43.6 (25.3 to 68) | 946.1 (525.4 to 1508.5) | 47.9 (26.8 to 76.1) | 0.34(0.15 to 0.52) | <0.001 |
| Lithuania | 61.4 (32.3 to 102) | 6.8 (3.6 to 11.2) | 32.7 (17.5 to 53.4) | 6 (3.3 to 9.8) | -0.39(-0.43 to -0.36) | <0.001 |
| Luxembourg | 56.6 (34.6 to 83.6) | 61.1 (37.5 to 89.8) | 85.4 (51.3 to 128.4) | 61.2 (37.1 to 90.8) | 0(0 to 0.01) | 0.287 |
| Madagascar | 5592.5 (3835.6 to 7914.1) | 177.8 (120.9 to 254) | 13674.1 (8866.2 to 19623.2) | 166.5 (107.4 to 241) | -0.23(-0.29 to -0.17) | <0.001 |
| Malawi | 5412.8 (3543.6 to 7678) | 202.2 (131.7 to 289) | 10282 (6657 to 14857.1) | 175 (112.4 to 255.6) | -0.51(-0.6 to -0.42) | <0.001 |
| Malaysia | 25915.5 (21316.7 to 31852.6) | 516.1 (423.9 to 635.3) | 8398.5 (5877.7 to 11886.1) | 98.4 (68.8 to 139.6) | -5.24(-5.48 to -5.01) | <0.001 |
| Maldives | 53.1 (35.2 to 75) | 92.5 (61 to 131.7) | 29.8 (19.8 to 42.5) | 27.4 (18.2 to 38.9) | -3.85(-3.89 to -3.82) | <0.001 |
| Mali | 2088.7 (1290.1 to 3092.6) | 98.8 (60.6 to 147.8) | 3871.1 (2369.1 to 5680.8) | 64.5 (39.1 to 96) | -1.37(-1.42 to -1.31) | <0.001 |
| Malta | 64.6 (39.5 to 97.5) | 73.7 (45.3 to 110.2) | 55 (33.6 to 82.3) | 65.9 (40.7 to 97.7) | -0.36(-0.41 to -0.31) | <0.001 |
| Marshall Islands | 1 (0.5 to 1.6) | 9.8 (5.2 to 15.7) | 0.9 (0.4 to 1.4) | 5.7 (3 to 9.3) | -1.73(-1.85 to -1.62) | <0.001 |
| Mauritania | 1869.3 (1187.8 to 2592.5) | 339 (214.8 to 472.4) | 2950.4 (1904.3 to 4202.8) | 239.9 (154.2 to 343.9) | -1.09(-1.23 to -0.96) | <0.001 |
| Mauritius | 273.4 (188.2 to 389.7) | 86.4 (59.2 to 123.6) | 125.4 (81.9 to 180.7) | 42 (27.6 to 60.2) | -2.3(-2.35 to -2.26) | <0.001 |
| Mexico | 10417.4 (6307.1 to 15547.8) | 42.5 (25.3 to 64.4) | 14296.9 (8487.7 to 21716) | 41.7 (24.9 to 63.1) | -0.06(-0.22 to 0.1) | 0.466 |
| Micronesia  (Federated States of) | 2.5 (1.3 to 3.9) | 10 (5.3 to 16) | 1.6 (0.8 to 2.5) | 5.9 (3 to 9.6) | -1.68(-1.75 to -1.6) | <0.001 |
| Monaco | 3.4 (2 to 5.2) | 55.1 (33.3 to 82.1) | 3.7 (2.2 to 5.6) | 57.8 (35.5 to 86.5) | 0.16(0.14 to 0.18) | <0.001 |
| Mongolia | 236.5 (133.5 to 385.4) | 39.4 (21.8 to 65) | 165.8 (94.1 to 264.3) | 20.7 (11.9 to 32.8) | -2.09(-2.22 to -1.95) | <0.001 |
| Montenegro | 26.9 (15 to 42.1) | 17.2 (9.6 to 26.9) | 18.6 (10.2 to 29.3) | 13.5 (7.5 to 21) | -0.79(-0.89 to -0.7) | <0.001 |
| Morocco | 5543.1 (3168.7 to 9404.5) | 82.6 (45.9 to 142.1) | 5316.2 (2874.3 to 8958.6) | 55.2 (30 to 92.8) | -1.3(-1.32 to -1.28) | <0.001 |
| Mozambique | 5435 (3202.5 to 8008.2) | 153.4 (89.6 to 227.6) | 6327 (3931.7 to 9335.3) | 74.7 (45.9 to 111.4) | -2.32(-2.41 to -2.24) | <0.001 |
| Myanmar | 16497.7 (11730.3 to 22273.9) | 138.4 (97.9 to 188.4) | 7803.5 (5203.4 to 11039.7) | 51.3 (34.3 to 72.6) | -3.18(-3.28 to -3.07) | <0.001 |
| Namibia | 201.7 (121.5 to 300.4) | 54.4 (32.6 to 81.7) | 281.4 (169.9 to 422.1) | 41.5 (25 to 62.5) | -0.86(-0.96 to -0.77) | <0.001 |
| Nauru | 0.1 (0.1 to 0.2) | 5.9 (3.2 to 9.3) | 0.1 (0.1 to 0.2) | 4.7 (2.5 to 7.5) | -0.74(-0.86 to -0.62) | <0.001 |
| Nepal | 5869.7 (3853.5 to 8353.9) | 114.3 (74.6 to 163.6) | 9344.1 (6153.4 to 13216.8) | 97.1 (63.8 to 137.8) | -0.49(-0.74 to -0.25) | <0.001 |
| Netherlands | 2374.9 (1459.8 to 3552.2) | 61.6 (38.1 to 91.8) | 2084.1 (1298.1 to 3117.3) | 60.4 (37.9 to 89.8) | -0.07(-0.08 to -0.05) | <0.001 |
| New Zealand | 155.6 (86.6 to 241.9) | 17.2 (9.6 to 26.8) | 193.8 (107.2 to 301.7) | 16.6 (9.2 to 25.9) | -0.11(-0.15 to -0.06) | <0.001 |
| Nicaragua | 318.5 (177.5 to 498.7) | 31.5 (17.2 to 50.3) | 539.7 (298.6 to 876.2) | 29.4 (16.3 to 47.8) | -0.23(-0.27 to -0.19) | <0.001 |
| Niger | 3583 (2135.3 to 5292.7) | 175.5 (103.4 to 262.1) | 13549.9 (8865 to 19367.8) | 208.6 (135.4 to 300.7) | 0.58(0.48 to 0.68) | <0.001 |
| Nigeria | 29494.2 (19077.3 to 41896.2) | 125 (79.9 to 179.6) | 62133.5 (39340.4 to 90111.3) | 97.3 (61.1 to 142.5) | -0.84(-1.09 to -0.59) | <0.001 |
| Niue | 0 (0 to 0.1) | 6.8 (3.7 to 10.8) | 0 (0 to 0) | 4.6 (2.4 to 7.4) | -1.26(-1.35 to -1.16) | <0.001 |
| North Macedonia | 93 (50.9 to 145.6) | 18.3 (10 to 28.6) | 69.2 (37.6 to 109.7) | 13.8 (7.6 to 21.7) | -0.91(-0.96 to -0.86) | <0.001 |
| Northern Mariana Islands | 0.7 (0.4 to 1.2) | 5.3 (2.9 to 8.3) | 0.5 (0.3 to 0.8) | 4.3 (2.3 to 6.8) | -0.68(-0.75 to -0.61) | <0.001 |
| Norway | 702 (439.9 to 1039.3) | 68.5 (43.2 to 100.9) | 763.5 (476 to 1125.9) | 67.7 (42.5 to 99.2) | -0.03(-0.06 to 0) | 0.067 |
| Oman | 103.9 (62.6 to 158.6) | 30.2 (17.9 to 46.4) | 245.3 (144 to 373.6) | 24.2 (14.3 to 36.8) | -0.72(-0.75 to -0.68) | <0.001 |
| Pakistan | 141032.4 (103589.2 to 183053.4) | 506.4 (371.3 to 658) | 441797.7 (331333.3 to 558278.2) | 666.5 (499.8 to 843) | 0.9(0.74 to 1.06) | <0.001 |
| Palau | 0.3 (0.1 to 0.4) | 6.3 (3.5 to 9.8) | 0.2 (0.1 to 0.3) | 4.5 (2.4 to 7.3) | -1.06(-1.09 to -1.04) | <0.001 |
| Palestine | 148.9 (89.5 to 227.3) | 33 (19.6 to 50.4) | 321.5 (191.1 to 489.4) | 24.7 (14.6 to 37.7) | -0.93(-0.95 to -0.9) | <0.001 |
| Panama | 95.4 (56.5 to 145.8) | 15 (8.8 to 23) | 131.7 (76.3 to 201.4) | 12.4 (7.2 to 18.9) | -0.61(-0.64 to -0.58) | <0.001 |
| Papua New Guinea | 107.1 (55 to 168.8) | 10.4 (5.4 to 16.7) | 157.4 (82.3 to 251.9) | 5.9 (3.1 to 9.5) | -1.84(-1.97 to -1.72) | <0.001 |
| Paraguay | 97.2 (51.3 to 152.9) | 10 (5.2 to 15.8) | 152.9 (80.6 to 246.2) | 8 (4.2 to 12.9) | -0.71(-0.74 to -0.68) | <0.001 |
| Peru | 332.8 (178.8 to 534.8) | 5.9 (3.2 to 9.6) | 487.9 (257.2 to 788) | 5.1 (2.7 to 8.2) | -0.5(-0.53 to -0.47) | <0.001 |
| Philippines | 17962.2 (11347.4 to 25708.8) | 104.8 (65.7 to 151.4) | 69889.4 (51774.5 to 90507.6) | 225.5 (166.9 to 292.4) | 2.51(2.35 to 2.67) | <0.001 |
| Poland | 1417.7 (798.2 to 2186.7) | 15.4 (8.7 to 23.7) | 1128.9 (623.2 to 1762.7) | 13.8 (7.7 to 21.2) | -0.37(-0.41 to -0.33) | <0.001 |
| Portugal | 2176.7 (1381.2 to 3210.6) | 87 (55.3 to 128) | 1653.3 (1024.7 to 2488) | 83.8 (52.9 to 123.2) | -0.12(-0.16 to -0.08) | <0.001 |
| Puerto Rico | 175.8 (102 to 263.7) | 18.3 (10.6 to 27.5) | 70.6 (39.9 to 109.1) | 9.7 (5.5 to 14.9) | -2.01(-2.12 to -1.9) | <0.001 |
| Qatar | 20.7 (12.4 to 31.1) | 26.1 (15.6 to 39.3) | 118.1 (69.3 to 178.4) | 21.3 (12.7 to 32.2) | -0.63(-0.71 to -0.56) | <0.001 |
| Republic of Korea | 2854.7 (1604.1 to 4498.5) | 22.1 (12.3 to 35) | 1783.6 (988.1 to 2823.2) | 16.3 (9.1 to 25.4) | -0.99(-1.02 to -0.95) | <0.001 |
| Republic of Moldova | 47.4 (25.8 to 74.6) | 4.3 (2.3 to 6.7) | 42.3 (22.3 to 69) | 5.1 (2.7 to 8.2) | 0.59(0.46 to 0.72) | <0.001 |
| Romania | 1378.4 (780.1 to 2138.9) | 24.6 (14 to 38) | 673.5 (356 to 1105.6) | 18 (9.6 to 29.1) | -1.04(-1.16 to -0.92) | <0.001 |
| Russian Federation | 3976.4 (2210.2 to 6236.1) | 11.1 (6.2 to 17.3) | 3494.1 (1923 to 5524.6) | 11.4 (6.4 to 17.7) | 0.07(0 to 0.14) | 0.066 |
| Rwanda | 5225.3 (3315.8 to 7379.1) | 270.8 (170.9 to 384.8) | 8205.2 (5379.8 to 11837.4) | 210.6 (137.4 to 304.9) | -0.82(-0.91 to -0.73) | <0.001 |
| Saint Kitts and Nevis | 2.9 (1.7 to 4.4) | 26.7 (15.6 to 40.9) | 1.8 (1 to 2.8) | 11.7 (6.5 to 18.1) | -2.63(-2.73 to -2.53) | <0.001 |
| Saint Lucia | 5 (3 to 7.5) | 13.7 (8.1 to 20.7) | 4.1 (2.3 to 6.4) | 9.2 (5.2 to 14.2) | -1.28(-1.32 to -1.25) | <0.001 |
| Saint Vincent  and the Grenadines | 11.8 (6.7 to 17.8) | 39 (21.9 to 60.1) | 4.8 (2.6 to 7.5) | 17.6 (9.5 to 27.4) | -2.52(-2.57 to -2.47) | <0.001 |
| Samoa | 1.9 (1 to 2.9) | 4.9 (2.7 to 7.6) | 1.9 (1 to 3) | 3.8 (1.9 to 6) | -0.87(-0.88 to -0.85) | <0.001 |
| San Marino | 3.7 (2.3 to 5.5) | 60.1 (37.6 to 88.8) | 3.9 (2.4 to 5.8) | 62.5 (39.2 to 91.7) | 0.13(0.12 to 0.14) | <0.001 |
| Sao Tome and Principe | 22.4 (14.1 to 33.2) | 77.2 (47.9 to 116) | 36.8 (22.1 to 55) | 62.3 (37.3 to 93.9) | -0.7(-0.79 to -0.62) | <0.001 |
| Saudi Arabia | 444 (272.2 to 659.9) | 13.4 (8.2 to 20) | 1075.2 (648.2 to 1599.9) | 10.7 (6.5 to 15.8) | -0.74(-0.77 to -0.71) | <0.001 |
| Senegal | 3688.5 (2592.4 to 5014) | 182.2 (127 to 250.6) | 4956.1 (2978.5 to 7303.3) | 115.4 (69 to 171.5) | -1.44(-1.6 to -1.29) | <0.001 |
| Serbia | 271.5 (154.3 to 412.3) | 11.8 (6.7 to 17.9) | 169.1 (93.9 to 265.1) | 8.6 (4.8 to 13.4) | -1.02(-1.06 to -0.98) | <0.001 |
| Seychelles | 14 (9.5 to 19.5) | 67.9 (45.8 to 95.3) | 8.7 (5.7 to 12.4) | 38.3 (25.4 to 54.6) | -1.83(-1.86 to -1.8) | <0.001 |
| Sierra Leone | 1277.6 (798.9 to 1844) | 113.2 (70.3 to 164.9) | 2454.2 (1454.3 to 3636.8) | 96.9 (57 to 145.1) | -0.49(-0.58 to -0.41) | <0.001 |
| Singapore | 176 (97.9 to 269.9) | 18.9 (10.6 to 28.9) | 221.9 (121.1 to 347.8) | 16.4 (9 to 25.3) | -0.47(-0.5 to -0.44) | <0.001 |
| Slovakia | 229 (125.5 to 360.8) | 17.5 (9.6 to 27.5) | 149.4 (80.6 to 237) | 12.7 (7 to 19.9) | -1.03(-1.05 to -1.01) | <0.001 |
| Slovenia | 81.3 (44.5 to 127.3) | 16.7 (9.2 to 26.1) | 49.7 (26.6 to 79.1) | 12.7 (6.9 to 19.9) | -0.89(-0.93 to -0.84) | <0.001 |
| Solomon Islands | 5.5 (3 to 8.5) | 6.9 (3.7 to 10.9) | 9.6 (5 to 15.6) | 5.5 (2.8 to 8.9) | -0.73(-0.75 to -0.7) | <0.001 |
| Somalia | 22347.8  (17156.1 to 28053.2) | 1161.6 (893.7 to 1455.1) | 74475.3 (57455.2 to 92125.1) | 1267.6  (983.2 to 1562.7) | 0.29 (0.25 to 0.33) | <0.001 |
| South Africa | 5260.9 (3092.2 to 7967.8) | 51.5 (30.1 to 78.4) | 7139.6 (4200.3 to 10826.8) | 46.4 (27.2 to 70.5) | -0.31(-0.5 to -0.13) | 0.001 |
| South Sudan | 1690.9 (1074.5 to 2422.6) | 112.5 (70.8 to 163.2) | 3154.8 (2010.1 to 4632.5) | 123.4 (78.3 to 182.3) | 0.29(0.17 to 0.42) | <0.001 |
| Spain | 6292.4 (3871.6 to 9245) | 64.9 (40 to 95.3) | 5344.9 (3224.5 to 8228.9) | 63.7 (39.2 to 95.3) | -0.05(-0.08 to -0.03) | <0.001 |
| Sri Lanka | 10471.9 (8147.7 to 13316.6) | 212.6 (164.9 to 271.3) | 5590.8 (3896.2 to 7807.6) | 104.5 (73.1 to 145.4) | -2.28(-2.31 to -2.25) | <0.001 |
| Sudan | 3678.1 (2044.3 to 5999.3) | 74.4 (40.5 to 122.3) | 5858.8 (3266.5 to 9442.7) | 50.9 (28.1 to 82.5) | -1.25(-1.48 to -1.03) | <0.001 |
| Suriname | 32.2 (18.1 to 48.2) | 30.7 (17.2 to 46.3) | 22.9 (12.5 to 35.9) | 16 (8.8 to 25) | -2.1(-2.17 to -2.04) | <0.001 |
| Sweden | 1378.7 (842.1 to 2058.6) | 72 (44.4 to 106.5) | 1428.8 (902.9 to 2077.1) | 70.5 (44.7 to 101.9) | -0.07(-0.09 to -0.04) | <0.001 |
| Switzerland | 619.1 (378.9 to 925.1) | 36.8 (22.7 to 54.6) | 689.4 (421.2 to 1026.9) | 38.5 (23.8 to 56.8) | 0.15(0.09 to 0.22) | <0.001 |
| Syrian Arab Republic | 1832.9 (1070 to 2875.4) | 62.5 (35.5 to 99.5) | 2069.3 (1161.6 to 3372.2) | 51.2 (28.3 to 83.5) | -0.65(-0.71 to -0.59) | <0.001 |
| Taiwan (Province of China) | 850.8 (505 to 1271.9) | 15.2 (8.9 to 22.7) | 510.7 (293.4 to 810.9) | 9.7 (5.6 to 15.2) | -1.48(-1.55 to -1.41) | <0.001 |
| Tajikistan | 448.4 (270.5 to 679.7) | 32.1 (18.9 to 49.5) | 774.8 (443 to 1233.8) | 29.9 (17.1 to 47.7) | -0.22(-0.34 to -0.11) | <0.001 |
| Thailand | 25278.9 (18324.3 to 33772.9) | 144.9 (104.7 to 194.3) | 7525.9 (4938.2 to 10976.4) | 53.1 (35.3 to 76.5) | -3.22(-3.34 to -3.09) | <0.001 |
| Timor-Leste | 208.7 (143.3 to 291.3) | 100.6 (68.7 to 141.3) | 179.6 (120.1 to 257.1) | 45.5 (30.2 to 65.6) | -2.49(-2.63 to -2.36) | <0.001 |
| Togo | 1971.8 (1486 to 2589.2) | 200.9 (148.9 to 269.4) | 3466.8 (2129.2 to 4962.5) | 151.3 (92.6 to 217.9) | -0.93(-0.98 to -0.87) | <0.001 |
| Tokelau | 0 (0 to 0.1) | 8.9 (4.8 to 14.1) | 0 (0 to 0) | 4.9 (2.5 to 7.8) | -1.94(-2 to -1.88) | <0.001 |
| Tonga | 2.1 (1.1 to 3.3) | 8.8 (4.7 to 14) | 1.4 (0.7 to 2.2) | 5.3 (2.8 to 8.5) | -1.66(-1.76 to -1.55) | <0.001 |
| Trinidad and Tobago | 160.8 (91.2 to 243.1) | 49.4 (27.9 to 75.2) | 72 (38.3 to 114.4) | 22.5 (12 to 35.5) | -2.53(-2.66 to -2.41) | <0.001 |
| Tunisia | 572 (346.1 to 879) | 27.8 (16.7 to 42.7) | 598.1 (361.5 to 902.5) | 19.5 (11.8 to 29.3) | -1.14(-1.18 to -1.11) | <0.001 |
| Turkey | 10444.1 (6058.7 to 17218.3) | 69.8 (39.6 to 116.1) | 9815.7 (5433.8 to 15900.5) | 45.9 (25.7 to 74.1) | -1.35(-1.4 to -1.31) | <0.001 |
| Turkmenistan | 210.7 (125.5 to 314.8) | 21.7 (12.7 to 32.9) | 244.5 (139.8 to 377.7) | 19.4 (11.1 to 30) | -0.37(-0.38 to -0.35) | <0.001 |
| Tuvalu | 0.3 (0.2 to 0.5) | 11.8 (6.1 to 18.8) | 0.2 (0.1 to 0.3) | 5.7 (2.9 to 9.2) | -2.32(-2.42 to -2.23) | <0.001 |
| Uganda | 4900.8 (3067.2 to 7175.5) | 108.4 (67.4 to 160.6) | 8190.9 (5109 to 11979.6) | 70.3 (43.4 to 103.9) | -1.42(-1.53 to -1.31) | <0.001 |
| Ukraine | 2114.5 (1225 to 3290.9) | 17.6 (10.3 to 27.2) | 1585 (911.3 to 2489.2) | 19 (11.4 to 29.1) | 0.21(0.03 to 0.39) | 0.026 |
| United Arab Emirates | 170.7 (100.7 to 266.4) | 50.6 (30 to 78.8) | 794.7 (421.7 to 1288.1) | 45.8 (25.3 to 73.9) | -0.32(-0.37 to -0.28) | <0.001 |
| United Kingdom | 9190.3 (5691.3 to 13569) | 66.8 (41.6 to 98.1) | 9471 (5896.9 to 13991.2) | 66.5 (41.7 to 97.5) | -0.01(-0.03 to 0.01) | 0.253 |
| United Republic of Tanzania | 3197 (2020 to 4677.2) | 48.2 (30.1 to 71.1) | 5570.6 (3395.4 to 8076.5) | 35.3 (21.4 to 51.6) | -1.02(-1.08 to -0.96) | <0.001 |
| United States of America | 10765.6 (6021.4 to 16636.1) | 16.4 (9.2 to 25.2) | 11998.1 (6717.4 to 18631.3) | 16.2 (9.1 to 25.1) | -2.46(-2.52 to -2.4) | <0.001 |
| United States Virgin Islands | 5.3 (3.1 to 8) | 18.9 (10.9 to 28.6) | 1.4 (0.8 to 2.3) | 8.7 (4.9 to 13.5) | -0.02(-0.06 to 0.02) | 0.347 |
| Uruguay | 68.6 (37.8 to 109.9) | 9.1 (5 to 14.6) | 62.5 (33.2 to 101.3) | 7.7 (4.1 to 12.4) | -0.56(-0.61 to -0.52) | <0.001 |
| Uzbekistan | 2212.7 (1328 to 3384.3) | 39.3 (23.1 to 61.2) | 2387.5 (1347.4 to 3836.5) | 28 (16 to 44.7) | -1.1(-1.22 to -0.98) | <0.001 |
| Vanuatu | 23.4 (14.4 to 33.2) | 57.8 (35.3 to 82.9) | 40.5 (26.5 to 57.1) | 47.6 (31.1 to 67.3) | -0.64(-0.67 to -0.6) | <0.001 |
| Venezuela (Bolivarian Republic of) | 1187.1 (663.9 to 1876.9) | 23.2 (12.9 to 37) | 1601.5 (888.7 to 2552.5) | 24.3 (13.4 to 38.5) | 0.14(0.1 to 0.18) | <0.001 |
| Viet Nam | 54050 (39403.3 to 72234.3) | 268.1 (194.8 to 359.8) | 19129.7 (13306.5 to 26789.4) | 82.2 (57.4 to 114.7) | -3.76(-3.82 to -3.71) | <0.001 |
| Yemen | 2179.4 (1238.4 to 3547.2) | 74.6 (41.1 to 124.2) | 6076.4 (3404.2 to 10386.7) | 68.3 (37 to 119.3) | -0.29(-0.38 to -0.2) | <0.001 |
| Zambia | 2477 (1536.2 to 3616.9) | 113.8 (70 to 168.9) | 4393.2 (2675.9 to 6434.2) | 80.5 (48.7 to 119) | -1.14(-1.27 to -1) | <0.001 |
| Zimbabwe | 5299.5 (3971.9 to 6777.2) | 187.8 (137.3 to 246.3) | 7945.5 (4680.9 to 11531.8) | 177.5 (104.2 to 259.6) | -0.23(-0.36 to -0.11) | <0.001 |
| **Location** | **Prevalence Number 1990 (95% UI)** | **ASPR 1990**  **(95% CI)** | **Prevalence Number 2021 (95% UI)** | **ASPR 2021**  **(95% CI)** | **AAPC (95% CI)** | ***P*** |
| Afghanistan | 51687.3 (37507.4 to 66824) | 2357.3 (1731.3 to 3039.4) | 162379.6 (116716.9 to 211624.3) | 2262.1 (1648.7 to 2932.9) | -0.16(-0.43 to 0.11) | 0.245 |
| Albania | 4483.8(3242.5 to 5887.3) | 538.3 (391 to 708.1) | 1979.7 (1407.1 to 2659) | 321.3 (227.9 to 431.6) | -1.68(-1.79 to -1.58) | <0.001 |
| Algeria | 67331 (49081.6 to 87899.9) | 1168 (857.1 to 1520.8) | 98810.6 (72108.3 to 128678.1) | 878.9 (639.1 to 1146) | -0.95(-1.04 to -0.85) | <0.001 |
| American Samoa | 10.7 (7.2 to 15.1) | 91.5 (62.2 to 128) | 7.8 (5.1 to 11.2) | 67 (43.6 to 96.6) | -1.01(-1.05 to -0.97) | <0.001 |
| Andorra | 188.2 (135.1 to 245.4) | 1230 (885 to 1602.6) | 270.2 (197.3 to 352.8) | 1278.5 (929 to 1668.6) | 0.14(0.11 to 0.17) | <0.001 |
| Angola | 476751.1 (376511.1 to 579175.6) | 21159 (16723.4 to 25724.2) | 961539.2 (749999.4 to 1179837.6) | 12770.5 (9979.4 to 15665.1) | -1.59(-1.77 to -1.42) | <0.001 |
| Antigua and Barbuda | 87.4 (63.7 to 113.5) | 539.1 (393.6 to 700.7) | 61.7 (44.1 to 82) | 252.9 (179.9 to 336.1) | -2.42(-2.5 to -2.34) | <0.001 |
| Argentina | 17622.1 (12162.4 to 23541.8) | 219.4 (151.7 to 293) | 22559 (15743.3 to 30419.9) | 190.1 (132.4 to 256.6) | -0.47(-0.49 to -0.45) | <0.001 |
| Armenia | 24155.7 (17925.1 to 30500.4) | 2753.7 (2045 to 3480.1) | 16141.6 (12013 to 20670.7) | 2216.5 (1632.9 to 2850) | -0.76(-1.01 to -0.52) | <0.001 |
| Australia | 14005.9 (9942.9 to 18774.1) | 312.2 (221.2 to 418.7) | 17789.1 (12585.6 to 23701.4) | 290.9 (204.7 to 387.8) | -0.22(-0.24 to -0.21) | <0.001 |
| Austria | 27111.6 (19807.8 to 35622.5) | 1346.8 (983.8 to 1768.5) | 27247 (19994.2 to 35610.8) | 1322.6 (967.9 to 1726) | -0.06(-0.07 to -0.05) | <0.001 |
| Azerbaijan | 12640 (9290.9 to 16455.6) | 656.3 (483.2 to 855.6) | 12845.6 (9391.9 to 16706.9) | 472.3 (342.9 to 614.7) | -1.06(-1.27 to -0.85) | <0.001 |
| Bahamas | 251.2(180.9 to 332.4) | 347.5 (250.9 to 459.9) | 244.3 (171.6 to 325.9) | 226 (158.5 to 301.3) | -1.39(-1.43 to -1.35) | <0.001 |
| Bahrain | 899.7 (667.2 to 1168.3) | 786 (583.3 to 1021.2) | 1924.2 (1399.4 to 2549.1) | 589.7 (427.9 to 781.5) | -0.94(-0.99 to -0.88) | <0.001 |
| Bangladesh | 3060121.5 (2816781.8 to 3300088.9) | 12807.4 (11776.7 to 13827.2) | 2374826 (1825174.6 to 3030862.3) | 5200.6 (3996.5 to 6639.6) | -2.92(-3.08 to -2.76) | <0.001 |
| Barbados | 560.7(407.5 to 732.7) | 816 (593.9 to 1067.7) | 489.5 (353.5 to 642) | 677.8 (488.1 to 888.1) | -0.6(-0.63 to -0.58) | <0.001 |
| Belarus | 7478.9 (5283.2 to 9962.8) | 294.9 (207.4 to 393.4) | 4927.1 (3477.5 to 6653.4) | 236.2 (164.9 to 320.1) | -0.75(-0.88 to -0.63) | <0.001 |
| Belgium | 33547.2 (24494.7 to 43822.3) | 1347.9 (983.9 to 1759) | 32919.9 (24081.2 to 43106.1) | 1284.9 (938.5 to 1681.8) | -0.16(-0.17 to -0.15) | <0.001 |
| Belize | 102.5 (72.5 to 135.9) | 246.7 (176.4 to 327) | 179 (122.9 to 244.6) | 149.2 (102.8 to 203.6) | -1.61(-1.66 to -1.55) | <0.001 |
| Benin | 22358.6 (16399.6 to 28677.9) | 2129.3 (1569.3 to 2732.1) | 50499.9 (36939.9 to 65632.2) | 1636.1 (1200.2 to 2124.7) | -0.88(-0.99 to -0.77) | <0.001 |
| Bermuda | 52.6 (37.8 to 69.9) | 303 (216.6 to 403) | 22.7 (15.9 to 30.6) | 165.3 (114.6 to 224) | -1.95(-2.03 to -1.88) | <0.001 |
| Bhutan | 3049.7 (2311 to 3956.7) | 2213.8 (1676.1 to 2880.5) | 2508.3 (1846.6 to 3268.5) | 1208.2 (889.8 to 1575) | -1.91(-2.02 to -1.79) | <0.001 |
| Bolivia (Plurinational State of) | 2341.1 (1715.4 to 3077.2) | 156.3 (115 to 205.5) | 3618.4 (2414.4 to 5008.5) | 116.4 (77.8 to 161.1) | -0.95(-0.96 to -0.93) | <0.001 |
| Bosnia and Herzegovina | 17738.7 (13022.3 to 22967.1) | 1518.6 (1115.4 to 1967.5) | 5432.2 (3910 to 7175.8) | 734 (526.1 to 970.9) | -2.39(-2.67 to -2.12) | <0.001 |
| Botswana | 1766.8 (1314.9 to 2312.3) | 575.4 (430.5 to 753.5) | 3144.9 (2310.4 to 4128.1) | 461.4 (338.6 to 606.1) | -0.72(-0.76 to -0.67) | <0.001 |
| Brazil | 63749.1 (44104.1 to 86500) | 165.4 (115 to 224.2) | 92083.1 (64225.2 to 124756.3) | 155.2 (107.7 to 210.6) | -0.21(-0.24 to -0.18) | <0.001 |
| Brunei Darussalam | 244.2 (175.2 to 323.5) | 363.6 (261.1 to 482.7) | 438.4 (314.5 to 584.2) | 346.9 (248.1 to 462.6) | -0.16(-0.19 to -0.14) | <0.001 |
| Bulgaria | 10346.2 (7437.5 to 13812.5) | 499.6 (358.1 to 666.9) | 4687.6 (3398.4 to 6340.4) | 325.8 (235.2 to 440.5) | -1.38(-1.41 to -1.36) | <0.001 |
| Burkina Faso | 53104 (39509.3 to 68497.4) | 2660.6 (1982.6 to 3435.3) | 81460 (59549.8 to 106039.7) | 1558.1 (1142 to 2025) | -1.72(-1.84 to -1.61) | <0.001 |
| Burundi | 94383.7 (83103.4 to 106218.7) | 7766 (6843.2 to 8739.8) | 132724.5 (100667.4 to 171260.5) | 4487.9 (3405.2 to 5804.9) | -1.78(-1.9 to -1.65) | <0.001 |
| Cabo Verde | 2448 (1838.9 to 3108) | 3289 (2475.8 to 4179.8) | 3302.4 (2382.7 to 4247) | 2201.2 (1592.4 to 2829.4) | -1.28(-1.4 to -1.16) | <0.001 |
| Cambodia | 165182.2 (127479.4 to 211472.6) | 6906.6 (5319.2 to 8860) | 63567.9 (46186 to 83293.3) | 1399.3 (1017.2 to 1833.2) | -5.04(-5.18 to -4.89) | <0.001 |
| Cameroon | 29653.8 (21960.5 to 38079.6) | 1304.2 (969.6 to 1674.4) | 92295.9 (67017.2 to 119158.6) | 1227.5 (893.9 to 1583.1) | -0.21(-0.26 to -0.15) | <0.001 |
| Canada | 25352.4 (18170.6 to 34099.2) | 340.5 (243 to 458.4) | 26918.7 (19122.8 to 36083.8) | 319.5 (225.9 to 428.2) | -0.21(-0.23 to -0.2) | <0.001 |
| Central African Republic | 100294.1 (89843.3 to 111206.8) | 15975.1 (14282 to 17740.1) | 155667.1 (121591.8 to 192610.2) | 11459.1 (8967.1 to 14157) | -1.03(-1.42 to -0.63) | <0.001 |
| Chad | 46830.8 (35806.3 to 59236.1) | 3651.3 (2791.5 to 4633.2) | 66640.1 (48094.3 to 85998.3) | 1841.7 (1335.9 to 2378.3) | -2.18(-2.24 to -2.12) | <0.001 |
| Chile | 11036 (7905.6 to 14676.1) | 301.6 (216.5 to 401.1) | 8947.6 (6188.7 to 12035.4) | 190.4 (130.9 to 256.5) | -1.48(-1.53 to -1.43) | <0.001 |
| China | 8147375.3 (6106429.1 to 10416479.8) | 2594.1 (1944.6 to 3322.4) | 11275570.6 (8659587.3 to 14030748) | 3303.6 (2537.3 to 4104.1) | 0.78(0.64 to 0.92) | <0.001 |
| Colombia | 70864.6 (51477.2 to 92199.7) | 817.2 (596.4 to 1063.5) | 97260.3 (70941.7 to 126452.2) | 740.8 (539.4 to 963.5) | -0.34(-0.4 to -0.28) | <0.001 |
| Comoros | 731.4 (539 to 958.7) | 712.9 (528.2 to 935.8) | 1343.2 (990.7 to 1745.1) | 697 (515.1 to 905.7) | -0.08(-0.16 to -0.01) | 0.024 |
| Congo | 115681.8 (91271.7 to 143563.1) | 20951.6 (16551.3 to 25958) | 275751.6 (218593 to 330774.5) | 19414.2 (15417 to 23274.3) | -0.22(-0.46 to 0.02) | 0.073 |
| Cook Islands | 4.9 (3.3 to 6.8) | 110.1 (75 to 152.2) | 3.1 (2 to 4.5) | 72 (47.3 to 103.6) | -1.35(-1.44 to -1.26) | <0.001 |
| Costa Rica | 5931.7 (4302 to 7705.9) | 762.7 (554.9 to 990.7) | 8672.5 (6314.9 to 11350.1) | 667.6 (484.9 to 875.1) | -0.43(-0.51 to -0.35) | <0.001 |
| Coted'Ivoire | 109746.4 (82332.8 to 140716.3) | 4223.9 (3168.2 to 5410.3) | 157885.7 (117700.9 to 203999.1) | 2449.3 (1830.1 to 3162.9) | -1.77(-1.89 to -1.64) | <0.001 |
| Croatia | 4961.8 (3594.5 to 6577) | 411.5 (297.2 to 546) | 2677.7 (1919.1 to 3588.3) | 296.2 (211 to 397.5) | -1.07(-1.12 to -1.02) | <0.001 |
| Cuba | 25097.1 (18319.3 to 33017.3) | 827 (603.9 to 1088.4) | 12702.9 (9142.5 to 16915.3) | 502.2 (360.2 to 668.7) | -1.65(-1.86 to -1.44) | <0.001 |
| Cyprus | 2871.7 (2109 to 3710) | 1437.7 (1056 to 1857.1) | 5297.1 (3880.5 to 6947.8) | 1392.1 (1018.3 to 1824.5) | -0.1(-0.13 to -0.08) | <0.001 |
| Czechia | 6636.5 (4756.1 to 8820.5) | 258.7 (185 to 344.2) | 4228.1 (2976.4 to 5667.1) | 183.2 (127.4 to 246.3) | -1.09(-1.16 to -1.02) | <0.001 |
| Democratic People's Republic of Korea | 8206.3 (5807.4 to 11069.2) | 147.3 (104.5 to 198.3) | 6764.6 (4671.3 to 9322.8) | 100.5 (69 to 138.7) | -1.23(-1.34 to -1.13) | <0.001 |
| Democratic Republic of the Congo | 2992705.4 (2787620.1 to 3207898.6) | 35911.9 (33452.7 to 38475.4) | 5475354.6 (4457317 to 6563733.9) | 26208.5 (21370.9 to 31411.9) | -1.02(-1.2 to -0.84) | <0.001 |
| Denmark | 16805.2 (12164.3 to 21984.4) | 1260.7 (910.7 to 1649.9) | 15321.7 (11172.2 to 19973.5) | 1185.5 (863.2 to 1543.9) | -0.21(-0.22 to -0.2) | <0.001 |
| Djibouti | 21522.3 (17001.3 to 26338) | 23655.6 (18623.1 to 28984.5) | 68516.5 (54333.5 to 84927.8) | 21121.1 (16752.4 to 26181.5) | -0.38(-0.44 to -0.32) | <0.001 |
| Dominica | 153.2 (112 to 197.5) | 922.3 (676.4 to 1188.7) | 71.3 (51.4 to 94.5) | 433.5 (312.8 to 575) | -2.42(-2.51 to -2.32) | <0.001 |
| Dominican Republic | 21804.5 (15806 to 28181.5) | 1186.2 (862.1 to 1536.4) | 15593.7 (11241 to 20621) | 538.5 (388.2 to 712.4) | -2.55(-2.59 to -2.5) | <0.001 |
| Ecuador | 2975.4 (1984.4 to 4155.4) | 120.4 (80.9 to 167.7) | 4731.4 (3164.7 to 6602.7) | 100.5 (67.2 to 140.2) | -0.58(-0.6 to -0.55) | <0.001 |
| Egypt | 445744.2 (339043.4 to 572185.7) | 3390.9 (2591.4 to 4340.1) | 352581.3 (255487.5 to 458879.9) | 1362.5 (989.4 to 1771.9) | -2.92(-3.07 to -2.76) | <0.001 |
| El Salvador | 13384.5 (9780 to 17400.3) | 1027.9 (755.6 to 1333.2) | 14521.8 (10638 to 18825.8) | 816.8 (598.2 to 1058.8) | -0.76(-0.88 to -0.63) | <0.001 |
| Equatorial Guinea | 46092.9 (38808.6 to 52927.5) | 47586.2 (40145.2 to 54589.6) | 35764.7 (27832.7 to 44078.5) | 9935.2 (7737 to 12253.9) | -4.91(-5.25 to -4.57) | <0.001 |
| Eritrea | 10267.6 (7574.8 to 13350.1) | 1358.9 (1007.4 to 1764.2) | 13934.2 (10251.5 to 18034.8) | 861.1 (635.2 to 1116.2) | -1.49(-1.59 to -1.38) | <0.001 |
| Estonia | 749.7 (519.7 to 1016.8) | 197.7 (136.4 to 268.6) | 439.8 (304.4 to 597.3) | 160.1 (109.5 to 218.4) | -0.73(-0.79 to -0.67) | <0.001 |
| Eswatini | 3510.2 (2607.7 to 4461.9) | 1899.8 (1419.6 to 2415.2) | 4426.3 (3245.9 to 5785.1) | 1436.5 (1057.5 to 1876.4) | -0.9(-0.93 to -0.86) | <0.001 |
| Ethiopia | 2779120.5 (2224176.5 to 3390743.1) | 26030.9 (20796.2 to 31788.5) | 4313123.1 (3379185.2 to 5320876.1) | 16517.5 (12918.1 to 20396.8) | -1.51(-1.72 to -1.3) | <0.001 |
| Fiji | 272.2 (186.2 to 375.9) | 141.7 (97.3 to 195.4) | 189.9 (123.9 to 270.9) | 83.1 (54.2 to 118.6) | -1.69(-1.77 to -1.61) | <0.001 |
| Finland | 17944.2 (13145.3 to 23528.1) | 1368.1 (1000.8 to 1793) | 15423.2 (11173.8 to 20177.6) | 1314.5 (951.3 to 1717.9) | -0.13(-0.17 to -0.09) | <0.001 |
| France | 196833.7 (142053.4 to 258140.9) | 1341.2 (968 to 1758.9) | 193485.2 (139823.1 to 252650.6) | 1331.2 (959.4 to 1738.4) | -0.03(-0.06 to 0) | 0.06 |
| Gabon | 26617.2 (21083.7 to 32318.2) | 12302.2 (9742.9 to 14967.3) | 23380.5 (17836.8 to 29403.4) | 4809.5 (3667.9 to 6057.4) | -2.94(-3.21 to -2.67) | <0.001 |
| Gambia | 18157.7 (14098.5 to 22702.4) | 8534.2 (6603.6 to 10678.6) | 34454 (25725.6 to 44186.8) | 5940.1 (4422.9 to 7636.9) | -1.18(-1.27 to -1.1) | <0.001 |
| Georgia | 10197.5 (7509.5 to 13216.1) | 740.3 (544.5 to 960.7) | 4942.4 (3600.3 to 6420.7) | 639.1 (463.2 to 828.9) | -0.48(-0.53 to -0.43) | <0.001 |
| Germany | 267404.9 (194969.1 to 348538.8) | 1335.2 (973.6 to 1738.9) | 231725.2 (169075.3 to 304334.9) | 1304 (950 to 1711) | -0.07(-0.09 to -0.06) | <0.001 |
| Ghana | 288393 (221401.2 to 363051) | 8611.1 (6596.5 to 10856.1) | 323216 (240086.3 to 414524.4) | 3620.5 (2690.4 to 4644.9) | -2.78(-2.86 to -2.7) | <0.001 |
| Greece | 42811.7 (31444.4 to 55243.7) | 1684.8 (1236.7 to 2173.6) | 35348.6 (25413.1 to 46796.3) | 1554.3 (1113.6 to 2057.9) | -0.26(-0.38 to -0.14) | <0.001 |
| Greenland | 46.6 (33.3 to 62.4) | 314.9 (225 to 422) | 40.2 (28.6 to 54.1) | 313.9 (222.5 to 422.7) | -0.01(-0.02 to 0) | 0.007 |
| Grenada | 183.2 (132.5 to 238.2) | 960.6 (697.4 to 1251.2) | 89.6 (64.6 to 119.5) | 349.5 (252 to 466.1) | -3.22(-3.26 to -3.18) | <0.001 |
| Guam | 31.9 (21.7 to 44.7) | 92 (62.7 to 128.5) | 25.7 (16.8 to 37.4) | 71 (46.3 to 103.2) | -0.84(-0.88 to -0.81) | <0.001 |
| Guatemala | 14760.7 (10706 to 19404.1) | 808.2 (590.9 to 1061.2) | 34738.8 (25274.6 to 45460.8) | 791.7 (577.8 to 1034.6) | -0.08(-0.13 to -0.03) | 0.003 |
| Guinea | 155767.7 (119139.7 to 197600.4) | 11711.9 (8930.7 to 14912.6) | 144055.6 (107813.3 to 185686.4) | 4554.1 (3411.8 to 5882.2) | -3.02(-3.15 to -2.89) | <0.001 |
| Guinea-Bissau | 26230.1 (20271.2 to 33188) | 11893.1 (9183 to 15067.8) | 31041.2 (23567.6 to 39412.4) | 6158.1 (4674.2 to 7821.8) | -2.11(-2.21 to -2.01) | <0.001 |
| Guyana | 2965.1 (2165.5 to 3808.8) | 1490 (1091 to 1919.5) | 1473 (1060.6 to 1943.3) | 728.2 (524.5 to 961.5) | -2.32(-2.44 to -2.2) | <0.001 |
| Haiti | 33359.4 (24789.8 to 42793.3) | 2215.8 (1649.1 to 2841.9) | 64045.8 (47579.9 to 82984.8) | 1816.5 (1350.9 to 2354.7) | -0.68(-0.79 to -0.58) | <0.001 |
| Honduras | 11681.8 (8541.9 to 15230.8) | 1115.6 (824.2 to 1449.9) | 28682.6 (20953.7 to 36787.8) | 1011.9 (742 to 1297) | -0.31(-0.39 to -0.24) | <0.001 |
| Hungary | 11827.4 (8556.2 to 15752.5) | 465.5 (336.1 to 620.4) | 6452.2 (4629.7 to 8601.5) | 299.5 (213.9 to 398.8) | -1.41(-1.5 to -1.37) | <0.001 |
| Iceland | 307.6 (226.6 to 403.3) | 473.2 (348.6 to 620.7) | 369.7 (271.6 to 486.7) | 456.3 (334.3 to 600.4) | -0.1(-0.15 to -0.05) | <0.001 |
| India | 28232548.4 (22472261.6 to 34277659.8) | 14221.5 (11318.2 to 17299.5) | 34259561.9 (26802828.8 to 42258757.5) | 9098.9 (7115.7 to 11227.3) | -1.44(-1.51 to -1.37) | <0.001 |
| Indonesia | 671960.8 (496680.4 to 869655.9) | 1441 (1066.9 to 1869.8) | 508731.1 (369763.4 to 668317.3) | 666.7 (483.8 to 875.1) | -2.47(-2.54 to -2.4) | <0.001 |
| Iran (Islamic Republic of) | 80847.1 (58184.8 to 105749.6) | 641.5 (466.1 to 838.5) | 103187.5 (74674.8 to 136349.6) | 442.5 (318 to 586.4) | -1.2(-1.33 to -1.07) | <0.001 |
| Iraq | 85947.7 (63220.5 to 111547.4) | 2133.7 (1584.9 to 2761.7) | 176191.2 (127791.2 to 227758.5) | 1675.7 (1221 to 2162.5) | -0.81(-1.02 to -0.6) | <0.001 |
| Ireland | 12625.9 (9137.8 to 16578) | 1446.3 (1046.7 to 1899.8) | 15505.7 (11308.5 to 20462.7) | 1287.3 (935.5 to 1700.3) | -0.38(-0.41 to -0.35) | <0.001 |
| Israel | 17358.1 (12544.8 to 22612.1) | 1437.7 (1039.2 to 1872.8) | 30317.5 (22113.2 to 39318.4) | 1356.1 (987.9 to 1758.9) | -0.18(-0.19 to -0.17) | <0.001 |
| Italy | 1293414.3 (1012443.9 to 1595935.9) | 8959.4 (7015.7 to 11048.6) | 759470.5 (581999.3 to 950642.6) | 5961.1 (4571.9 to 7446.3) | -1.29(-1.42 to -1.16) | <0.001 |
| Jamaica | 5319.2 (3875.9 to 6886.3) | 912.1 (666.2 to 1183.2) | 3975.2 (2870.2 to 5218.2) | 512.2 (369.9 to 672.8) | -1.85(-1.88 to -1.81) | <0.001 |
| Japan | 134817.1 (98939.9 to 178719.6) | 417.3 (306 to 552.5) | 96162.8 (70137.2 to 128128) | 378.9 (275 to 503.5) | -0.31(-0.32 to -0.3) | <0.001 |
| Jordan | 8783.4 (6504.5 to 11338.3) | 1057.1 (787.5 to 1363.1) | 25165.9 (18282.3 to 33118.8) | 813.7 (593.4 to 1069.7) | -0.87(-0.94 to -0.8) | <0.001 |
| Kazakhstan | 58981.2 (43693.2 to 75285.6) | 1418.6 (1052.1 to 1812.1) | 36213.9 (26323.4 to 47581.1) | 770.7 (558.6 to 1013.4) | -1.98(-2.11 to -1.84) | <0.001 |
| Kenya | 86464.2 (64257.4 to 110458.9) | 1768.7 (1322.5 to 2262) | 215155.7 (158575.4 to 276500.6) | 1687.5 (1247.4 to 2169.8) | -0.16(-0.22 to -0.1) | <0.001 |
| Kiribati | 38.6 (26.6 to 52) | 210.4 (145.7 to 284.1) | 40.1 (26.9 to 56.7) | 126.8 (85.3 to 179.2) | -1.65(-1.74 to -1.56) | <0.001 |
| Kuwait | 2953.1 (2177 to 3870.8) | 716.4 (528.3 to 940) | 8234.8 (6021.1 to 10837.9) | 562.1 (406.1 to 741.8) | -0.8(-0.83 to -0.76) | <0.001 |
| Kyrgyzstan | 6580.3 (4858.2 to 8560.2) | 609.8 (451 to 793.7) | 9431.7 (6815.8 to 12392.8) | 545.1 (393.6 to 716.6) | -0.36(-0.41 to -0.32) | <0.001 |
| Lao People's Democratic Republic | 13928.2 (10193.5 to 18009.6) | 1495.8 (1094.1 to 1934.7) | 14161.7 (10186.9 to 18641.6) | 717.4 (516.9 to 944.6) | -2.34(-2.47 to -2.21) | <0.001 |
| Latvia | 1236.7 (860.5 to 1672.8) | 191.8 (132.8 to 259.6) | 650.8 (452.7 to 882.7) | 169.4 (116.4 to 230.4) | -0.44(-0.51 to -0.37) | <0.001 |
| Lebanon | 28115.1 (22894.2 to 33119.9) | 3708.3 (3011.5 to 4378.7) | 28114.5 (20525.9 to 36198.2) | 1893 (1372.6 to 2440.5) | -2.16(-2.23 to -2.09) | <0.001 |
| Lesotho | 86589.9 (81361.4 to 92073.7) | 23250.2 (21832.7 to 24736.4) | 29842.9 (22194 to 38040.7) | 6100.7 (4536.5 to 7787.8) | -4.27(-4.37 to -4.18) | <0.001 |
| Liberia | 6973 (5129.3 to 8933.5) | 1309.7 (968.2 to 1679.7) | 16412.6 (12015.2 to 21282.1) | 1216.1 (892.2 to 1577.6) | -0.29(-0.44 to -0.13) | <0.001 |
| Libya | 12170.6 (8947.1 to 15700.8) | 1350.5 (1002.6 to 1739.7) | 33228.6 (24256.2 to 42683.9) | 1665.5 (1211.5 to 2143.1) | 0.69(0.59 to 0.79) | <0.001 |
| Lithuania | 1728.7 (1191.6 to 2319.9) | 187.9 (129.2 to 252.2) | 904.8 (624.2 to 1214.5) | 159.7 (109.2 to 214.8) | -0.55(-0.59 to -0.51) | <0.001 |
| Luxembourg | 1281.8 (936 to 1661.8) | 1275.4 (931.5 to 1652.4) | 2061.8 (1486.2 to 2697.5) | 1262.4 (909.7 to 1649.5) | -0.03(-0.05 to -0.01) | 0.002 |
| Madagascar | 95297.3 (70905.1 to 123279.8) | 3813 (2834.8 to 4933.6) | 268105.8 (201136.4 to 346017.7) | 3894.4 (2922.9 to 5032.3) | 0.04(-0.02 to 0.1) | 0.18 |
| Malawi | 103232.5 (78595.4 to 132747.3) | 4871.7 (3709.7 to 6277.7) | 191153.7 (141058.5 to 247434.5) | 4091.8 (3016 to 5312.1) | -0.61(-0.81 to -0.41) | <0.001 |
| Malaysia | 488502.4 (420809.3 to 568744.2) | 11202.8 (9642 to 13048.5) | 153629.3 (112904.2 to 203744.6) | 1803.7 (1326.5 to 2391.2) | -5.77(-6.02 to -5.51) | <0.001 |
| Maldives | 790.1 (572.5 to 1032.3) | 1752.8 (1270.1 to 2300) | 529.4 (387.1 to 692.7) | 440.9 (322 to 577.1) | -4.37(-4.41 to -4.32) | <0.001 |
| Mali | 43414.5 (31962.8 to 55707.3) | 2355.8 (1734.9 to 3023) | 73391.9 (53881.7 to 94255.3) | 1432 (1055.4 to 1838.4) | -1.6(-1.64 to -1.55) | <0.001 |
| Malta | 1533.8 (1121.7 to 2021.6) | 1572.4 (1148.7 to 2071.3) | 1365.2 (1002.8 to 1775) | 1376.6 (1010.2 to 1785.6) | -0.43(-0.49 to -0.38) | <0.001 |
| Marshall Islands | 17.3 (11.9 to 23.7) | 184.6 (127.7 to 252.7) | 14.9 (9.9 to 21.2) | 101.7 (67.3 to 144) | -1.92(-1.97 to -1.86) | <0.001 |
| Mauritania | 39421.3 (30077.2 to 50080) | 8826 (6712.6 to 11244.7) | 58007.2 (42924.1 to 74720) | 5716.2 (4224.2 to 7378.7) | -1.38(-1.45 to -1.31) | <0.001 |
| Mauritius | 4793.5 (3536.6 to 6240.3) | 1599.3 (1180.8 to 2084.4) | 2243.9 (1619.9 to 2991) | 696.9 (502.5 to 928.4) | -2.65(-2.7 to -2.59) | <0.001 |
| Mexico | 301582 (226595.3 to 381089.1) | 1386 (1046.4 to 1749.8) | 504574.9 (382550.1 to 636569.2) | 1439.6 (1090.5 to 1816) | 0.12(0.03 to 0.21) | 0.008 |
| Micronesia (Federated States of) | 42 (28.8 to 57.9) | 188.5 (130.2 to 259.9) | 27 (17.9 to 38) | 105.4 (70.4 to 148.2) | -1.85(-1.92 to -1.78) | <0.001 |
| Monaco | 83.1 (60.8 to 108.6) | 1134.7 (827.8 to 1484.2) | 88.2 (64.3 to 114.3) | 1189.2 (864.2 to 1539) | 0.15(0.14 to 0.16) | <0.001 |
| Mongolia | 10081.2 (7499.4 to 12816.6) | 1930.7 (1436.1 to 2469.8) | 5933.6 (4367.6 to 7740.3) | 708.1 (519.4 to 923.5) | -3.26(-3.49 to -3.03) | <0.001 |
| Montenegro | 650.5 (470.3 to 868) | 416.3 (300.9 to 555.8) | 446.5 (319.4 to 596.4) | 308.5 (219.7 to 412.4) | -0.98(-1.01 to -0.95) | <0.001 |
| Morocco | 283780.3 (212035.8 to 361446) | 4492.9 (3394 to 5703.6) | 208844.9 (153946.4 to 270984.3) | 2155.1 (1586 to 2797.7) | -2.37(-2.47 to -2.26) | <0.001 |
| Mozambique | 128315.4 (97578.4 to 161894.3) | 4206.2 (3200.1 to 5319) | 118718.4 (87425 to 152647.8) | 1655.3 (1222.6 to 2128.6) | -3.03(-3.16 to -2.91) | <0.001 |
| Myanmar | 274345.9 (206110.4 to 349026.2) | 2732.4 (2050.3 to 3480.3) | 134354.1 (97408.9 to 177051.3) | 893.7 (647.8 to 1177.7) | -3.57(-3.68 to -3.46) | <0.001 |
| Namibia | 4344.7 (3231.8 to 5577.1) | 1332 (995.5 to 1708.8) | 5837.3 (4330.3 to 7598.8) | 897.1 (666.4 to 1167.9) | -1.26(-1.41 to -1.11) | <0.001 |
| Nauru | 2.6 (1.7 to 3.5) | 107.1 (72.5 to 148) | 2.2 (1.4 to 3.2) | 80.2 (52.2 to 113) | -0.93(-1.15 to -0.71) | <0.001 |
| Nepal | 122827.1 (91827.9 to 158269.4) | 2758.7 (2062.4 to 3553.8) | 189894.5 (139009.8 to 247109.7) | 2123.2 (1553.9 to 2765) | -0.85(-1.07 to -0.62) | <0.001 |
| Netherlands | 52061.9 (38007.2 to 68342.7) | 1291.3 (942.5 to 1694.2) | 46835.7 (34219.1 to 61121.5) | 1246.4 (909.4 to 1625.5) | -0.13(-0.17 to -0.09) | <0.001 |
| New Zealand | 3266.1 (2356 to 4357.4) | 360.7 (260 to 481.4) | 4184.6 (3018 to 5564.9) | 346.2 (248.7 to 460.4) | -0.13(-0.14 to -0.12) | <0.001 |
| Nicaragua | 9357.3 (6812 to 12166.4) | 1044.3 (767.2 to 1350.8) | 18083.1 (13311.2 to 23292.4) | 996.3 (734 to 1283.5) | -0.17(-0.31 to -0.04) | 0.011 |
| Niger | 94995 (74618.8 to 117020.8) | 5644.5 (4411 to 6987.3) | 240596 (181138.5 to 309677.5) | 4953.2 (3721.7 to 6399.8) | -0.46(-0.57 to -0.34) | <0.001 |
| Nigeria | 559282 (420962.4 to 701268.6) | 2941.3 (2217.5 to 3695.8) | 1148970.4 (855609.6 to 1467603.8) | 2118.4 (1580.4 to 2706.3) | -1.13(-1.48 to -0.78) | <0.001 |
| Niue | 0.6 (0.4 to 0.8) | 124.6 (85.1 to 172.1) | 0.3 (0.2 to 0.4) | 78.9 (51.2 to 111.9) | -1.48(-1.53 to -1.43) | <0.001 |
| North Macedonia | 2282.2 (1641.3 to 3014.8) | 448.1 (322.1 to 592) | 1702.2 (1237.8 to 2262) | 317.5 (229.6 to 422.6) | -1.11(-1.14 to -1.08) | <0.001 |
| Northern Mariana Islands | 12.7 (8.6 to 17.8) | 92.3 (62.7 to 130) | 8.5 (5.6 to 12.2) | 72.9 (48.1 to 105.4) | -0.78(-0.82 to -0.73) | <0.001 |
| Norway | 14817 (10799.4 to 19220.3) | 1385.2 (1008.5 to 1796.6) | 16940.8 (12355 to 21903.8) | 1358.9 (989.7 to 1756.1) | -0.06(-0.07 to -0.05) | <0.001 |
| Oman | 2786.4 (2048.2 to 3630.7) | 826.3 (610.1 to 1076.5) | 6363.9 (4589.2 to 8344.5) | 627.8 (450.4 to 825.5) | -0.89(-0.91 to -0.87) | <0.001 |
| Pakistan | 2809107.1 (2212613.2 to 3450560.6) | 12566.2 (9870.1 to 15470.2) | 9239273.6 (7325204.4 to 11319298.6) | 15582.4 (12354.6 to 19094.7) | 0.7(0.64 to 0.77) | <0.001 |
| Palau | 4.7 (3.2 to 6.5) | 114.9 (78.6 to 159) | 3 (2 to 4.2) | 77.2 (50.7 to 110.7) | -1.29(-1.32 to -1.25) | <0.001 |
| Palestine | 4086.8 (2975.6 to 5361.1) | 930.5 (682.2 to 1216.3) | 8123.1 (5835 to 10703.5) | 626.5 (452.8 to 824.5) | -1.28(-1.36 to -1.2) | <0.001 |
| Panama | 2556.2 (1819.5 to 3368.5) | 414.8 (297.2 to 545.8) | 3320 (2368.3 to 4409.5) | 310.6 (221.6 to 412.5) | -0.95(-1.01 to -0.88) | <0.001 |
| Papua New Guinea | 1842.4 (1255.9 to 2547.7) | 197.1 (135.2 to 272.1) | 2714 (1802.9 to 3827.5) | 104.9 (69.9 to 147.9) | -2.04(-2.15 to -1.93) | <0.001 |
| Paraguay | 2016.6 (1381.7 to 2756) | 218 (150.3 to 297) | 3155.2 (2184 to 4315.4) | 166.7 (115.5 to 228.1) | -0.87(-0.88 to -0.85) | <0.001 |
| Peru | 6397.2 (4253.9 to 8935.3) | 120.2 (80.6 to 167.5) | 9566.8 (6417.9 to 13556) | 98.9 (66.2 to 140.4) | -0.63(-0.67 to -0.6) | <0.001 |
| Philippines | 366081.5 (276223.1 to 461260.6) | 2441.4 (1848.9 to 3079.6) | 1223393.2 (938641.6 to 1534198.8) | 4237.3 (3251.3 to 5317.8) | 1.79(1.61 to 1.97) | <0.001 |
| Poland | 34083.2 (24977.6 to 44902.8) | 361 (263.7 to 476.4) | 28024.4 (20499.8 to 37109) | 313.4 (226.8 to 415.2) | -0.48(-0.53 to -0.43) | <0.001 |
| Portugal | 46052.3 (34244.1 to 59217.7) | 1818.8 (1351.2 to 2339.3) | 43486.7 (31860.3 to 57029) | 1786.6 (1303.7 to 2345.3) | -0.06(-0.09 to -0.02) | 0.001 |
| Puerto Rico | 3932 (2866.6 to 5190) | 411 (299.7 to 542.5) | 1532.2 (1080.2 to 2050.4) | 202.7 (142.3 to 271.4) | -2.25(-2.32 to -2.19) | <0.001 |
| Qatar | 524.5 (382.8 to 687.6) | 666.2 (484.6 to 874.9) | 2875.5 (2110.1 to 3726.1) | 519.3 (375.8 to 676.4) | -0.81(-0.88 to -0.75) | <0.001 |
| Republic of Korea | 61757.7 (44831.4 to 82230.2) | 491.3 (357.2 to 654.3) | 40472.2 (29417.9 to 54023.7) | 341.5 (246.5 to 455.5) | -1.17(-1.2 to -1.14) | <0.001 |
| Republic of Moldova | 1211.8 (811.8 to 1644.7) | 107.7 (71.9 to 146.3) | 1152.2 (798.3 to 1578.4) | 132.4 (90.4 to 182.3) | 0.65(0.51 to 0.79) | <0.001 |
| Romania | 39195.5 (31210.1 to 48021.1) | 697.2 (554.5 to 854.7) | 17733.6 (12805.7 to 23677.6) | 433.3 (311.3 to 577.3) | -1.53(-1.6 to -1.47) | <0.001 |
| Russian Federation | 126380.3 (91704.1 to 166140) | 341.4 (246.5 to 449.4) | 116186 (84455.6 to 152873.2) | 348.6 (251 to 459.2) | 0.04(-0.09 to 0.16) | 0.551 |
| Rwanda | 106621.1 (81797.9 to 134816.8) | 6915.1 (5279.7 to 8776.5) | 167522.7 (126017.3 to 215806.2) | 4942.8 (3715.2 to 6375) | -1.09(-1.22 to -0.95) | <0.001 |
| Saint Kitts and Nevis | 63.2 (45.8 to 82.3) | 640.6 (466.2 to 835.7) | 39.3 (27.9 to 52.8) | 249.1 (176.4 to 335.4) | -3(-3.08 to -2.92) | <0.001 |
| Saint Lucia | 101.5 (72.7 to 135) | 300.8 (217 to 400.2) | 88.5 (62.4 to 119.2) | 191.9 (134.6 to 258.8) | -1.44(-1.47 to -1.41) | <0.001 |
| Saint Vincent and the Grenadines | 255.4 (185.1 to 330.2) | 997.9 (726.4 to 1289.3) | 111.8 (80.5 to 148.5) | 400.5 (287.7 to 531.7) | -2.9(-2.94 to -2.86) | <0.001 |
| Samoa | 30.2 (19.9 to 42.8) | 85.4 (57.3 to 120.1) | 30.2 (19.4 to 44.1) | 63.6 (41.2 to 92.4) | -0.96(-0.98 to -0.94) | <0.001 |
| San Marino | 78.2 (57.3 to 102.1) | 1251.5 (917.6 to 1633.9) | 95.8 (70.7 to 124.5) | 1298.6 (955.9 to 1687.8) | 0.12(0.1 to 0.14) | <0.001 |
| Sao Tome and Principe | 435.3 (323.7 to 563.3) | 1808.2 (1351.4 to 2338.8) | 741.8 (541.8 to 961.6) | 1366.9 (1000.1 to 1772.4) | -0.91(-0.95 to -0.88) | <0.001 |
| Saudi Arabia | 10590 (7523.4 to 13788.9) | 322.7 (231.2 to 418.9) | 24406.4 (17550.7 to 31804.2) | 240.9 (171.9 to 314.6) | -0.94(-1.01 to -0.86) | <0.001 |
| Senegal | 75654.3 (62065.8 to 90050.6) | 4659.6 (3820.2 to 5543.9) | 100195.6 (74292.5 to 128265.4) | 2678.7 (1987.6 to 3434.5) | -1.75(-1.87 to -1.64) | <0.001 |
| Serbia | 6213.5 (4488.8 to 8296.6) | 265.8 (191.6 to 355) | 3748.6 (2624.8 to 5052.1) | 183.5 (127.8 to 247.7) | -1.19(-1.26 to -1.12) | <0.001 |
| Seychelles | 217 (159.3 to 284.2) | 1228.5 (904.2 to 1608.6) | 154.7 (111.6 to 204.1) | 627.9 (452.1 to 827.7) | -2.15(-2.17 to -2.12) | <0.001 |
| Sierra Leone | 26359.7 (19789.4 to 33675.9) | 2726.6 (2051 to 3491.9) | 48254.7 (35375 to 63081.8) | 2228 (1637.1 to 2913.4) | -0.65(-0.72 to -0.58) | <0.001 |
| Singapore | 3838.4 (2787.4 to 5108.1) | 408.5 (296 to 544.5) | 5171.3 (3730.5 to 6898.1) | 343.9 (245.4 to 459.3) | -0.55(-0.56 to -0.54) | <0.001 |
| Slovakia | 5612.4 (4061.6 to 7391) | 422.1 (305 to 555.9) | 3688.8 (2640.6 to 4934.2) | 289.5 (205.5 to 387.8) | -1.24(-1.3 to -1.19) | <0.001 |
| Slovenia | 2005.2 (1456 to 2661.6) | 401.4 (290.7 to 533) | 1219.5 (867.6 to 1637.5) | 287.5 (203.3 to 386.2) | -1.08(-1.13 to -1.03) | <0.001 |
| Solomon Islands | 89.4 (60.2 to 123.9) | 126 (85.8 to 173.9) | 164.6 (108.8 to 234.7) | 98 (65.1 to 139.3) | -0.81(-0.84 to -0.78) | <0.001 |
| Somalia | 479416 (388667 to 577804.1) | 29638.2 (24015.7 to 35724.4) | 1452229 (1168797.1 to 1730219.8) | 32237.8 (25911.1 to 38381) | 0.27(0.23 to 0.32) | <0.001 |
| South Africa | 113828 (85021.1 to 147168.6) | 1205.7 (904 to 1557.2) | 152428.9 (112949.8 to 198061.5) | 976.6 (723.1 to 1269.3) | -0.68(-0.88 to -0.47) | <0.001 |
| South Sudan | 31717.2 (23641.4 to 40150.1) | 2612.1 (1949.5 to 3312.3) | 62424.8 (46919.8 to 80228.1) | 2841.2 (2136.3 to 3663.3) | 0.25(0.1 to 0.39) | 0.001 |
| Spain | 131159.7 (95709.8 to 171284.8) | 1366.2 (996.8 to 1784.3) | 137592.5 (100686.4 to 180616) | 1323.2 (963 to 1736.8) | -0.11(-0.11 to -0.1) | <0.001 |
| Sri Lanka | 222111 (191803.6 to 255989.3) | 4905.4 (4238.5 to 5654.1) | 108678.3 (79960.4 to 143210.3) | 1904.3 (1400.3 to 2509.1) | -3.01(-3.09 to -2.94) | <0.001 |
| Sudan | 144687.6 (108725.1 to 184433.1) | 3112.5 (2360.7 to 3960.5) | 207679.7 (149623.4 to 269608.8) | 1847.9 (1341.1 to 2394.4) | -1.72(-2.05 to -1.39) | <0.001 |
| Suriname | 722.5 (522.6 to 940.4) | 755 (547.4 to 983.4) | 517.3 (373.7 to 689.8) | 355.2 (256.3 to 473.6) | -2.42(-2.52 to -2.32) | <0.001 |
| Sweden | 30833.2 (22181.9 to 40366.7) | 1467.8 (1052.7 to 1919.8) | 32262.6 (23621.2 to 42010.4) | 1422.1 (1040.1 to 1850.7) | -0.1(-0.11 to -0.09) | <0.001 |
| Switzerland | 13499.7 (9951.8 to 17681.9) | 749 (551.4 to 980.4) | 15327.9 (11189.5 to 20234.5) | 751.2 (546.4 to 989.1) | 0.01(-0.02 to 0.04) | 0.539 |
| Syrian Arab Republic | 66061.3 (48291.1 to 85096.3) | 2413.7 (1787 to 3089.4) | 72202.7 (52206.7 to 93360.8) | 1852.1 (1346.4 to 2391.6) | -0.86(-1.01 to -0.72) | <0.001 |
| Taiwan (Province of China) | 13126.4 (9211.8 to 17723.1) | 238.7 (167.8 to 322.4) | 9024.2 (6338.2 to 12452.9) | 151.5 (105.4 to 209.4) | -1.49(-1.54 to -1.44) | <0.001 |
| Tajikistan | 18701.4 (13646.6 to 23862.6) | 1510.3 (1110.8 to 1928.3) | 33383.1 (24375.5 to 43332.5) | 1303.3 (953.4 to 1692.2) | -0.49(-0.69 to -0.29) | <0.001 |
| Thailand | 414637.6 (319105.8 to 536134.8) | 2634 (2026.5 to 3403.4) | 155154.5 (112349.7 to 207042.5) | 920.2 (664.7 to 1228.2) | -3.36(-3.49 to -3.23) | <0.001 |
| Timor-Leste | 3464.4 (2602.1 to 4501.9) | 1890.9 (1422.1 to 2459.1) | 2553.4 (1845.6 to 3369.3) | 782.8 (566.9 to 1035) | -2.77(-2.97 to -2.57) | <0.001 |
| Togo | 46330.9 (41037.4 to 51986.4) | 5707.4 (5059.8 to 6405.3) | 74830.2 (55455 to 96133) | 3549.6 (2633.3 to 4563.2) | -1.57(-1.68 to -1.46) | <0.001 |
| Tokelau | 0.6 (0.4 to 0.8) | 165.9 (114.7 to 227.8) | 0.3 (0.2 to 0.4) | 86.2 (56.4 to 123.6) | -2.11(-2.17 to -2.04) | <0.001 |
| Tonga | 35.1 (23.7 to 48.2) | 163.9 (111.9 to 224.6) | 23.2 (15 to 33.2) | 93.5 (60.8 to 133.7) | -1.81(-1.88 to -1.73) | <0.001 |
| Trinidad and Tobago | 3958.4 (2929.1 to 5076.4) | 1293.8 (958.5 to 1659.6) | 1825.1 (1322.2 to 2415.1) | 527.4 (380.6 to 698) | -2.89(-3.03 to -2.74) | <0.001 |
| Tunisia | 14876.5 (11056.5 to 19321.1) | 732 (545.3 to 949.2) | 14776.2 (10786.3 to 19352.5) | 480.6 (349.2 to 630.6) | -1.34(-1.36 to -1.32) | <0.001 |
| Turkey | 483801.7 (358547.2 to 614456.7) | 3388.4 (2526.3 to 4293.6) | 333544.8 (245075 to 430359.4) | 1534.7 (1123 to 1982.8) | -2.57(-2.67 to -2.47) | <0.001 |
| Turkmenistan | 7013.1 (5077.1 to 9069.8) | 771.8 (562.3 to 1001) | 7915.8 (5781.2 to 10313.4) | 628.9 (459.4 to 819.5) | -0.66(-0.7 to -0.63) | <0.001 |
| Tuvalu | 5.5 (3.8 to 7.5) | 224.5 (154.5 to 308.5) | 2.9 (1.9 to 4.1) | 101.7 (67.7 to 143.6) | -2.52(-2.59 to -2.45) | <0.001 |
| Uganda | 90827.7 (67332.7 to 115814.6) | 2535.3 (1884.9 to 3238.2) | 149130.9 (109570.5 to 193346.1) | 1518.4 (1121.1 to 1969.1) | -1.69(-1.77 to -1.61) | <0.001 |
| Ukraine | 108303 (79239.9 to 138720) | 861.8 (628.7 to 1104.5) | 98360.8 (72424.8 to 125475.7) | 1005.3 (738.3 to 1283) | 0.52(0.33 to 0.72) | <0.001 |
| United Arab Emirates | 6971.3 (5154.2 to 8895.4) | 2036.6 (1510.9 to 2596.1) | 26301.5 (19387.7 to 33844.8) | 1505.1 (1093 to 1950.9) | -0.96(-1.07 to -0.84) | <0.001 |
| United Kingdom | 196441.9 (143760.1 to 253883.6) | 1360.4 (994.6 to 1757.4) | 212058.6 (155050.2 to 273862.3) | 1337.8 (976.2 to 1727.3) | -0.06(-0.07 to -0.04) | <0.001 |
| United Republic of Tanzania | 58821.5 (43260.3 to 76230) | 1031.2 (763.3 to 1335.8) | 102603.4 (75370.3 to 134358.6) | 713.2 (526.2 to 936.2) | -1.22(-1.29 to -1.14) | <0.001 |
| United States of America | 231524.6 (167333.7 to 307768.9) | 341.8 (246 to 455) | 258738.4 (187014.3 to 343449.1) | 338.6 (244 to 449.4) | -0.02(-0.03 to -0.01) | <0.001 |
| United States Virgin Islands | 123 (89.7 to 162.5) | 428.3 (312.3 to 565.6) | 31 (21.8 to 42.2) | 178.8 (125.2 to 243.1) | -2.76(-2.83 to -2.69) | <0.001 |
| Uruguay | 1761.1 (1229.8 to 2367.6) | 234.6 (163.8 to 315.3) | 1599.4 (1110.6 to 2150.7) | 192.8 (133.4 to 259.4) | -0.62(-0.69 to -0.55) | <0.001 |
| Uzbekistan | 110653 (82370.6 to 140835.5) | 2207.7 (1654.3 to 2799.9) | 101506.3 (75114.4 to 129752.2) | 1142 (843 to 1460.7) | -2.13(-2.27 to -1.99) | <0.001 |
| Vanuatu | 411.7 (294.9 to 545.6) | 1226.6 (878.5 to 1629.6) | 755.5 (547.9 to 995.8) | 990.1 (717.4 to 1307.4) | -0.7(-0.75 to -0.66) | <0.001 |
| Venezuela (Bolivarian Republic of) | 32174.4 (23249.4 to 42468.5) | 664.8 (482.5 to 877.3) | 51751.2 (38085.8 to 67207) | 753.7 (553.9 to 978.2) | 0.4(0.26 to 0.53) | <0.001 |
| Viet Nam | 896213.6 (677209.1 to 1159020.6) | 5493 (4150.1 to 7114) | 396576.7 (291977.8 to 521621) | 1492.2 (1098.4 to 1961) | -4.14(-4.19 to -4.1) | <0.001 |
| Yemen | 94189.8 (69427.4 to 120489.6) | 3443.4 (2565.4 to 4388.5) | 324066.3 (236983.9 to 419411.4) | 3862.1 (2842.5 to 4976.2) | 0.38(0.21 to 0.55) | <0.001 |
| Zambia | 45218.1 (33575.7 to 57657.1) | 2662.3 (1981.7 to 3401.7) | 83513.1 (60844.5 to 108334.7) | 1782.2 (1303.5 to 2312.2) | -1.32(-1.45 to -1.18) | <0.001 |
| Zimbabwe | 136625.9 (125139.8 to 148591.4) | 5982.7 (5486 to 6506.4) | 172671.7 (127510.1 to 222320.3) | 4408.7 (3258 to 5683.9) | -1.01(-1.11 to -0.91) | <0.001 |
| **Location** | **YLD 1990 (95% UI)** | **ASYR 1990**  **(95% CI)** | **YLD 2021 (95% UI)** | **ASYR 2021 (95% CI)** | **AAPC (95% CI)** | ***P*** |
| Afghanistan | 1156.7 (572.6 to 1927.7) | 52.3 (26.3 to 87.1) | 3674.6 (1848.7 to 6183) | 50.5 (25.6 to 84.7) | 0.11 (-0.25 to 0.03) | 0.117 |
| Albania | 93.3 (33.7 to 178.8) | 11.2 (4.1 to 21.4) | 40.8 (14.7 to 76.7) | 6.6 (2.4 to 12.5) | -1.69 (-1.85 to 1.54) | <0.001 |
| Algeria | 1393.7 (634 to 2435.8) | 24.1 (10.9 to 42) | 2058.3 (923 to 3733.4) | 18.3 (8.2 to 33.3) | -0.9(-0.99 to -0.82) | <0.001 |
| American Samoa | 0.1 (0 to 0.3) | 1 (0.3 to 2.2) | 0.1 (0 to 0.2) | 0.7 (0.2 to 1.6) | -1.07(-1.36 to -0.77) | <0.001 |
| Andorra | 2 (0.9 to 4) | 13.2 (5.7 to 26) | 2.9 (1.3 to 5.6) | 13.7 (6 to 26.5) | 0.14(0.1 to 0.18) | <0.001 |
| Angola | 5256.9 (2418.4 to 9783) | 232.5 (107.4 to 434.3) | 10661 (4913.6 to 19755.8) | 141.1 (65.1 to 262.6) | -1.58(-1.75 to -1.4) | <0.001 |
| Antigua and Barbuda | 1.5 (0.6 to 3.1) | 9.4 (3.5 to 18.8) | 1.2 (0.5 to 2.1) | 4.9 (2 to 8.8) | -2.05(-2.16 to -1.95) | <0.001 |
| Argentina | 188.9 (71.3 to 376.6) | 2.4 (0.9 to 4.7) | 243 (90 to 496.2) | 2 (0.8 to 4.2) | -0.46(-0.51 to -0.42) | <0.001 |
| Armenia | 260.1 (109.3 to 499.1) | 29.6 (12.5 to 56.9) | 173.6 (73.8 to 331.3) | 23.9 (10.1 to 45.7) | -0.76(-1 to -0.51) | <0.001 |
| Australia | 149.4 (61.6 to 308) | 3.3 (1.4 to 6.9) | 190.5 (74.3 to 390.5) | 3.1 (1.2 to 6.4) | -0.21(-0.28 to -0.15) | <0.001 |
| Austria | 290.4 (127.3 to 566.5) | 14.4 (6.3 to 28.2) | 292 (127.2 to 572.3) | 14.2 (6.2 to 27.8) | -0.06(-0.08 to -0.03) | <0.001 |
| Azerbaijan | 250.4 (97.9 to 466.3) | 13 (5.1 to 24.3) | 138.2 (56.6 to 276.2) | 5.1 (2.1 to 10.1) | -2.96(-3.63 to -2.28) | <0.001 |
| Bahamas | 4.4 (1.8 to 8.3) | 6.1 (2.5 to 11.4) | 4.6 (1.7 to 8.5) | 4.3 (1.6 to 7.9) | -1.13(-1.24 to -1.02) | <0.001 |
| Bahrain | 18.7 (8.1 to 34.7) | 16.3 (7.1 to 30) | 20.5 (8.5 to 40.6) | 6.3 (2.6 to 12.5) | -3.04(-3.18 to -2.9) | <0.001 |
| Bangladesh | 36246.7 (18401.6 to 64298.8) | 150.5 (76.2 to 267.3) | 27725.7 (13277.6 to 51933.7) | 60.6 (29 to 113.6) | -2.93(-3.07 to -2.8) | <0.001 |
| Barbados | 8.6 (3.7 to 17) | 12.6 (5.3 to 24.7) | 7.8 (3.1 to 15.2) | 10.8 (4.4 to 21) | -0.49(-0.64 to -0.34) | <0.001 |
| Belarus | 154.7 (68.7 to 273.9) | 6.1 (2.7 to 10.8) | 102.8 (42.1 to 179.7) | 4.9 (2 to 8.6) | -0.75(-0.9 to -0.6) | <0.001 |
| Belgium | 359.6 (155.3 to 713.7) | 14.5 (6.2 to 28.7) | 351.4 (150.6 to 689.3) | 13.7 (5.9 to 26.9) | -0.16(-0.2 to -0.12) | <0.001 |
| Belize | 2.3 (1.1 to 3.8) | 5.5 (2.7 to 9.1) | 3.9 (1.8 to 6.9) | 3.3 (1.5 to 5.7) | -1.61(-1.68 to -1.54) | <0.001 |
| Benin | 339.3 (161.2 to 604.6) | 31.7 (15.2 to 56.5) | 794.5 (360.6 to 1473.7) | 25.1 (11.6 to 46.7) | -0.75(-0.86 to -0.65) | <0.001 |
| Bermuda | 0.9 (0.4 to 1.7) | 5.2 (2.1 to 9.8) | 0.4 (0.1 to 0.7) | 2.7 (1 to 5.5) | -2.08(-2.15 to -2.01) | <0.001 |
| Bhutan | 61.7 (31.9 to 105.1) | 44.2 (22.9 to 75.6) | 26.9 (11.6 to 52.8) | 13 (5.6 to 25.4) | -3.83(-4.07 to -3.59) | <0.001 |
| Bolivia (Plurinational State of) | 52.6 (27.9 to 89.2) | 3.5 (1.9 to 5.9) | 80.8 (38.9 to 141.5) | 2.6 (1.3 to 4.5) | -0.97(-1 to -0.93) | <0.001 |
| Bosnia and Herzegovina | 336.1 (155.4 to 597.2) | 28.8 (13.3 to 51.1) | 97.1 (37.4 to 188.9) | 13.1 (5 to 25.7) | -2.57(-2.86 to -2.28) | <0.001 |
| Botswana | 31 (12.2 to 59) | 10 (3.9 to 19.1) | 33.5 (13.6 to 66.7) | 4.9 (2 to 9.8) | -2.26(-2.47 to -2.06) | <0.001 |
| Brazil | 729.5 (310.3 to 1413.4) | 1.9 (0.8 to 3.7) | 980.8 (389 to 1962.1) | 1.7 (0.7 to 3.3) | -0.43(-0.46 to -0.4) | <0.001 |
| Brunei Darussalam | 2.6 (1.1 to 5.4) | 3.9 (1.6 to 8) | 4.7 (2 to 9.5) | 3.7 (1.6 to 7.5) | -0.17(-0.23 to -0.12) | <0.001 |
| Bulgaria | 111.4 (45.6 to 219.9) | 5.4 (2.2 to 10.6) | 50.4 (19.9 to 101.6) | 3.5 (1.4 to 7) | -1.48(-1.88 to -1.07) | <0.001 |
| Burkina Faso | 800.3 (391.9 to 1391.5) | 39.3 (19.3 to 68.7) | 1351.1 (595.6 to 2511.5) | 25.3 (11.2 to 47) | -1.42(-1.53 to -1.3) | <0.001 |
| Burundi | 1158.7 (602.3 to 2042) | 94.4 (48.9 to 166.5) | 1857.6 (955.5 to 3299.3) | 61.4 (31.6 to 109.7) | -1.42(-1.55 to -1.28) | <0.001 |
| Cabo Verde | 32.8 (16.3 to 59.6) | 43.3 (21.7 to 78.8) | 35.4 (15.4 to 69.2) | 23.6 (10.2 to 46.1) | -1.98(-2.11 to -1.85) | <0.001 |
| Cambodia | 2306.1 (1254.2 to 4063.7) | 95.1 (51.5 to 168.4) | 1189.3 (570.3 to 1983.3) | 26.2 (12.5 to 43.6) | -4.16(-4.34 to -3.99) | <0.001 |
| Cameroon | 475 (204.8 to 871.3) | 20.4 (8.9 to 37.5) | 1505.1 (664.3 to 2838.7) | 19.6 (8.8 to 37) | -0.14(-0.21 to -0.08) | <0.001 |
| Canada | 272.2 (110.2 to 551.7) | 3.7 (1.5 to 7.4) | 290.1 (115.7 to 573.9) | 3.4 (1.4 to 6.8) | -0.2(-0.23 to -0.17) | <0.001 |
| Central African Republic | 1138.9 (569.6 to 2044.9) | 180.4 (90.1 to 323.9) | 1849 (915.7 to 3313.9) | 135.2 (66.9 to 243) | -0.89(-1.24 to -0.55) | <0.001 |
| Chad | 653.2 (336 to 1167.6) | 50.1 (25.8 to 89.8) | 1045 (493.1 to 1910.6) | 28.1 (13.4 to 51.2) | -1.84(-1.95 to -1.73) | <0.001 |
| Chile | 118.2 (49.7 to 235.7) | 3.2 (1.4 to 6.4) | 96.1 (34.3 to 197.7) | 2 (0.7 to 4.2) | -1.47(-1.58 to -1.36) | <0.001 |
| China | 102893.9 (52404.9 to 188819.3) | 32.6 (16.6 to 60) | 121863.7 (56048.2 to 235918.6) | 35.8 (16.4 to 69.1) | 0.31(0.21 to 0.41) | <0.001 |
| Colombia | 1190.4 (494.5 to 2312.7) | 13.8 (5.7 to 26.6) | 1545.7 (651.7 to 2981.6) | 11.8 (5 to 22.7) | -0.49(-0.76 to -0.22) | <0.001 |
| Comoros | 14.1 (5.7 to 26.4) | 13.6 (5.5 to 25.3) | 25.8 (9.7 to 49.1) | 13.3 (5.1 to 25.3) | -0.07(-0.21 to 0.08) | 0.387 |
| Congo | 1270.3 (581.6 to 2389.6) | 229.1 (105.2 to 429.7) | 2936.4 (1325.3 to 5492) | 206.6 (93.5 to 386.4) | -0.31(-0.56 to -0.06) | 0.017 |
| Cook Islands | 0.1 (0.1 to 0.2) | 2.4 (1.2 to 4.2) | 0 (0 to 0.1) | 0.8 (0.2 to 1.8) | -3.68(-3.78 to -3.58) | <0.001 |
| Costa Rica | 96.4 (40.5 to 186.1) | 12.5 (5.2 to 24) | 92.8 (38.7 to 179.2) | 7.1 (3 to 13.8) | -1.77(-2.09 to -1.44) | <0.001 |
| Coted'Ivoire | 1355.6 (658.8 to 2527.6) | 51.5 (25 to 96.4) | 2118.8 (1021.3 to 3900.3) | 32.5 (15.7 to 59.9) | -1.49(-1.62 to -1.35) | <0.001 |
| Croatia | 53.6 (21.5 to 106.8) | 4.4 (1.8 to 8.9) | 28.9 (11.4 to 58.4) | 3.2 (1.3 to 6.5) | -1.06(-1.13 to -1) | <0.001 |
| Cuba | 426.9 (171.1 to 813.8) | 14 (5.6 to 26.7) | 241.3 (85.8 to 480) | 9.6 (3.4 to 19) | -1.25(-1.42 to -1.09) | <0.001 |
| Cyprus | 30.8 (13.6 to 60) | 15.4 (6.8 to 30) | 56.8 (25.1 to 113.7) | 14.9 (6.6 to 29.9) | -0.1(-0.13 to -0.06) | <0.001 |
| Czechia | 71.8 (27.7 to 147.5) | 2.8 (1.1 to 5.8) | 45.4 (17.2 to 96) | 2 (0.7 to 4.2) | -1.11(-1.21 to -1.01) | <0.001 |
| Democratic People's Republic of Korea | 183.1 (90.7 to 308.2) | 3.3 (1.6 to 5.5) | 153.1 (78.9 to 264.6) | 2.3 (1.2 to 3.9) | -1.25(-1.53 to -0.96) | <0.001 |
| Democratic Republic of the Congo | 32464.4 (16046.4 to 58622.1) | 388.1 (191.3 to 701.1) | 61179.6 (28699.4 to 112822.2) | 291.7 (137.2 to 539.2) | -0.93(-1.11 to -0.75) | <0.001 |
| Denmark | 180 (78.5 to 353.8) | 13.5 (5.9 to 26.6) | 163.9 (70.7 to 324) | 12.7 (5.5 to 25) | -0.21(-0.25 to -0.18) | <0.001 |
| Djibouti | 237.5 (114.1 to 431.5) | 259.8 (125 to 472.1) | 756.5 (362.2 to 1438.3) | 233.2 (111.7 to 443.5) | -0.37(-0.44 to -0.3) | <0.001 |
| Dominica | 2.6 (1.1 to 4.8) | 15.5 (6.7 to 28.7) | 1.3 (0.5 to 2.6) | 8.1 (2.9 to 15.9) | -2.09(-2.2 to -1.98) | <0.001 |
| Dominican Republic | 348.6 (151.8 to 648.5) | 18.8 (8.3 to 34.9) | 265.3 (101.2 to 522.7) | 9.2 (3.5 to 18.1) | -2.3(-2.47 to -2.12) | <0.001 |
| Ecuador | 66.4 (32.3 to 116.5) | 2.7 (1.3 to 4.7) | 50.9 (18 to 107.7) | 1.1 (0.4 to 2.3) | -2.93(-3.04 to -2.82) | <0.001 |
| Egypt | 8560.6 (4566 to 14381.7) | 64.8 (34.7 to 108.9) | 3763.4 (1538.9 to 7135.3) | 14.5 (6 to 27.6) | -4.74(-4.99 to -4.49) | <0.001 |
| El Salvador | 241.3 (102 to 440.4) | 18.4 (7.9 to 33.7) | 244.5 (100.5 to 460.3) | 13.8 (5.6 to 25.9) | -0.92(-1.11 to -0.74) | <0.001 |
| Equatorial Guinea | 498.9 (237.1 to 898) | 513.7 (245.2 to 924.6) | 390.9 (176.9 to 737.8) | 108.3 (49.1 to 205.1) | -4.88(-5.21 to -4.55) | <0.001 |
| Eritrea | 188.7 (87.3 to 345.2) | 24.5 (11.4 to 45) | 267 (107.5 to 482.6) | 16.3 (6.7 to 29.6) | -1.34(-1.46 to -1.21) | <0.001 |
| Estonia | 16 (6.8 to 28) | 4.2 (1.8 to 7.4) | 8.9 (3.1 to 16.1) | 3.2 (1.1 to 5.9) | -0.91(-0.96 to -0.86) | <0.001 |
| Eswatini | 47.3 (22.6 to 87.5) | 25.3 (12.2 to 46.8) | 47 (20.4 to 91.2) | 15.2 (6.6 to 29.5) | -1.63(-1.71 to -1.55) | <0.001 |
| Ethiopia | 32241.4 (16163.8 to 58751.7) | 299.5 (149.7 to 548.5) | 48996.2 (23969.5 to 89352.5) | 186.3 (91.1 to 341.2) | -1.57(-1.78 to -1.36) | <0.001 |
| Fiji | 6.1 (3.1 to 10.4) | 3.2 (1.6 to 5.4) | 2 (0.6 to 4.6) | 0.9 (0.3 to 2) | -3.81(-4.68 to -2.93) | <0.001 |
| Finland | 191.9 (84.3 to 381.4) | 14.6 (6.4 to 29) | 164.7 (72.7 to 324.1) | 14 (6.2 to 27.6) | -0.13(-0.19 to -0.08) | <0.001 |
| France | 2105.1 (909.1 to 4096) | 14.3 (6.2 to 27.9) | 2065.2 (908.3 to 4023.1) | 14.2 (6.2 to 27.6) | -0.03(-0.09 to 0.02) | 0.231 |
| Gabon | 290.8 (131.6 to 542.2) | 134 (60.8 to 250.4) | 264.8 (125.9 to 488.6) | 54.3 (25.9 to 100.6) | -2.83(-3.08 to -2.57) | <0.001 |
| Gambia | 213.7 (105.7 to 393.9) | 99.3 (49.1 to 183.7) | 423.5 (211.8 to 783.3) | 72 (36 to 133.8) | -1.05(-1.14 to -0.97) | <0.001 |
| Georgia | 110.2 (45.9 to 209.9) | 8 (3.3 to 15.3) | 53.2 (22.2 to 104.6) | 6.9 (2.9 to 13.5) | -0.48(-0.52 to -0.44) | <0.001 |
| Germany | 2868 (1243.1 to 5762.3) | 14.3 (6.2 to 28.7) | 2476.9 (1082.1 to 4947.6) | 14 (6.1 to 27.8) | -0.09(-0.12 to -0.05) | <0.001 |
| Ghana | 3379.8 (1665 to 6191.8) | 100.1 (49 to 183.8) | 3982.6 (1962.7 to 7312.2) | 44.4 (21.9 to 81.7) | -2.61(-2.69 to -2.53) | <0.001 |
| Greece | 457.9 (203.3 to 897.7) | 18 (8 to 35.3) | 378.2 (168.3 to 756.9) | 16.6 (7.4 to 33.2) | -0.26(-0.38 to -0.14) | <0.001 |
| Greenland | 0.5 (0.2 to 1) | 3.4 (1.3 to 6.8) | 0.4 (0.2 to 0.9) | 3.4 (1.3 to 7) | 0(-0.03 to 0.04) | 0.816 |
| Grenada | 3.2 (1.3 to 6) | 16.6 (6.9 to 30.8) | 1.8 (0.7 to 3.5) | 6.9 (2.6 to 13.6) | -2.78(-2.86 to -2.69) | <0.001 |
| Guam | 0.3 (0.1 to 0.7) | 1 (0.3 to 2.1) | 0.3 (0.1 to 0.6) | 0.8 (0.2 to 1.7) | -0.83(-0.95 to -0.71) | <0.001 |
| Guatemala | 275.6 (113.8 to 520.4) | 15 (6.2 to 28.4) | 590.5 (235.3 to 1114.8) | 13.5 (5.4 to 25.3) | -0.35(-0.5 to -0.2) | <0.001 |
| Guinea | 1788.5 (888.4 to 3387.8) | 133.9 (66.4 to 254.6) | 1834.5 (903 to 3375.7) | 57.2 (28.2 to 105.6) | -2.71(-2.8 to -2.62) | <0.001 |
| Guinea-Bissau | 304 (149.1 to 580.7) | 136.7 (66.8 to 261.7) | 381.4 (189.1 to 697.8) | 74.9 (37 to 137.4) | -1.93(-2.02 to -1.84) | <0.001 |
| Guyana | 49.5 (22.6 to 87.1) | 24.5 (11.2 to 43.1) | 26.4 (10.3 to 49.8) | 13 (5.1 to 24.5) | -2.04(-2.16 to -1.91) | <0.001 |
| Haiti | 501.7 (246.5 to 903) | 32.9 (16.2 to 59.3) | 1024.9 (489.4 to 1819) | 29 (13.9 to 51.5) | -0.46(-0.56 to -0.36) | <0.001 |
| Honduras | 212.1 (93.9 to 388.7) | 20.2 (9 to 36.8) | 498.4 (208.3 to 931.9) | 17.5 (7.4 to 32.7) | -0.42(-0.77 to -0.08) | 0.015 |
| Hungary | 127.2 (51.2 to 255.2) | 5 (2 to 10) | 69.6 (27.8 to 144.5) | 3.2 (1.3 to 6.7) | -1.41(-1.51 to -1.32) | <0.001 |
| Iceland | 3.3 (1.4 to 6.4) | 5.1 (2.1 to 9.9) | 4 (1.6 to 7.9) | 4.9 (2 to 9.8) | -0.1(-0.16 to -0.04) | 0.001 |
| India | 521712 (330546.3 to 809404.3) | 259 (163.8 to 402.7) | 422300.2 (219391.1 to 744750.1) | 111.9 (58.1 to 197.7) | -2.71(-2.88 to -2.54) | <0.001 |
| Indonesia | 16279.8 (9440.1 to 26219.1) | 34.6 (20 to 55.5) | 5931.9 (2788.6 to 11375.9) | 7.8 (3.7 to 14.9) | -4.69(-5.07 to -4.31) | <0.001 |
| Iran (Islamic Republic of) | 2206.7 (1234.1 to 3630.5) | 17.5 (9.9 to 28.8) | 2195.8 (1154.3 to 3698.9) | 9.4 (4.9 to 15.9) | -2(-2.12 to -1.88) | <0.001 |
| Iraq | 1603 (816.5 to 2729.9) | 39.4 (20.3 to 67.2) | 3295.4 (1565.1 to 5664.3) | 31.2 (14.9 to 53.7) | -0.77(-0.93 to -0.62) | <0.001 |
| Ireland | 135.1 (59 to 272.9) | 15.5 (6.8 to 31.2) | 165.9 (72.6 to 323.7) | 13.8 (6 to 26.9) | -0.38(-0.4 to -0.35) | <0.001 |
| Israel | 186 (78.4 to 363.7) | 15.4 (6.5 to 30.1) | 325.3 (143.1 to 642.3) | 14.6 (6.4 to 28.7) | -0.18(-0.21 to -0.15) | <0.001 |
| Italy | 13845.4 (6398.9 to 26464.7) | 95.9 (44.3 to 183.3) | 8133.2 (3738 to 15744.8) | 63.9 (29.2 to 123) | -1.28(-1.43 to -1.13) | <0.001 |
| Jamaica | 92.9 (39.6 to 175.1) | 15.8 (6.8 to 29.8) | 75.1 (26.8 to 152) | 9.7 (3.5 to 19.6) | -1.59(-1.74 to -1.43) | <0.001 |
| Japan | 1454.9 (604.6 to 2891.3) | 4.5 (1.9 to 8.9) | 1037.2 (432.1 to 2041) | 4.1 (1.7 to 8) | -0.31(-0.33 to -0.3) | <0.001 |
| Jordan | 186.6 (84.1 to 330.8) | 22.4 (10.2 to 39.5) | 530.9 (228 to 952.8) | 17.1 (7.4 to 30.8) | -0.89(-1.04 to -0.74) | <0.001 |
| Kazakhstan | 926.1 (431.8 to 1679.8) | 22.3 (10.4 to 40.5) | 387.7 (163.3 to 762.3) | 8.3 (3.5 to 16.2) | -3.2(-3.47 to -2.94) | <0.001 |
| Kenya | 926.7 (404.8 to 1769.2) | 18.9 (8.3 to 36.3) | 2302.3 (1007.7 to 4439) | 18 (7.9 to 34.9) | -0.16(-0.23 to -0.1) | <0.001 |
| Kiribati | 0.9 (0.5 to 1.5) | 4.7 (2.6 to 8.2) | 0.9 (0.5 to 1.6) | 2.9 (1.5 to 5) | -1.64(-1.72 to -1.56) | <0.001 |
| Kuwait | 31.6 (13.3 to 61.3) | 7.7 (3.2 to 14.9) | 87.6 (36.1 to 173) | 6 (2.5 to 11.8) | -0.8(-0.88 to -0.71) | <0.001 |
| Kyrgyzstan | 70.8 (29.5 to 138.9) | 6.6 (2.7 to 12.9) | 101.8 (43.2 to 199.1) | 5.9 (2.5 to 11.5) | -0.35(-0.42 to -0.28) | <0.001 |
| Lao People's Democratic Republic | 364.2 (185 to 609.5) | 38.5 (19.6 to 64.3) | 152.9 (67 to 303.2) | 7.7 (3.4 to 15.4) | -5.13(-5.53 to -4.73) | <0.001 |
| Latvia | 25.4 (9.9 to 45.2) | 3.9 (1.5 to 7) | 12.9 (4.9 to 23.9) | 3.3 (1.2 to 6.1) | -0.62(-0.69 to -0.55) | <0.001 |
| Lebanon | 300.1 (138.7 to 559.5) | 39.5 (18.3 to 73.8) | 298.8 (124.4 to 578.6) | 20.1 (8.4 to 39) | -2.16(-2.24 to -2.08) | <0.001 |
| Lesotho | 962.9 (471.8 to 1751.8) | 258 (126.2 to 468.6) | 349.7 (169.5 to 646.6) | 71 (34.4 to 131.2) | -4.12(-4.21 to -4.04) | <0.001 |
| Liberia | 121.3 (55.3 to 219.6) | 22.3 (10.3 to 40.3) | 299.4 (134.8 to 544.3) | 21.9 (9.9 to 39.9) | -0.08(-0.21 to 0.04) | 0.192 |
| Libya | 218.9 (98.3 to 389) | 24.2 (11 to 43.1) | 616 (294.3 to 1055.8) | 30.9 (14.7 to 53.1) | 0.81(0.68 to 0.94) | <0.001 |
| Lithuania | 35.5 (14.5 to 62.8) | 3.8 (1.6 to 6.8) | 18 (6.5 to 33) | 3.1 (1.1 to 5.8) | -0.68(-0.77 to -0.6) | <0.002 |
| Luxembourg | 13.7 (6 to 26.4) | 13.6 (5.9 to 26.3) | 22.1 (9.5 to 43.8) | 13.5 (5.8 to 26.8) | -0.03(-0.07 to 0.01) | 0.188 |
| Madagascar | 1292.5 (647.5 to 2344) | 50.5 (25.5 to 92.3) | 3619.9 (1791.9 to 6518.1) | 51.7 (25.7 to 93.6) | 0.05(0 to 0.1) | 0.032 |
| Malawi | 1360.5 (677.3 to 2431.2) | 63.1 (31.4 to 112.8) | 2616.6 (1293.4 to 4713.5) | 54.7 (27.1 to 99) | -0.49(-0.65 to -0.33) | <0.001 |
| Malaysia | 5824 (2906.5 to 10470.6) | 133 (66.5 to 239.6) | 2253.5 (1058.2 to 4125.2) | 26.5 (12.4 to 48.6) | -5.1(-5.48 to -4.73) | <0.001 |
| Maldives | 8.5 (3.8 to 16.9) | 18.8 (8.3 to 37.4) | 5.7 (2.4 to 11.6) | 4.7 (2 to 9.6) | -4.37(-4.45 to -4.28) | <0.001 |
| Mali | 682.7 (331.6 to 1209.9) | 36.3 (17.7 to 64.4) | 1215.9 (535.9 to 2186.2) | 23.1 (10.4 to 41.4) | -1.45(-1.5 to -1.39) | <0.001 |
| Malta | 16.4 (7.3 to 31.9) | 16.9 (7.4 to 32.7) | 14.6 (6.5 to 28.7) | 14.8 (6.5 to 28.8) | -0.44(-0.52 to -0.36) | <0.001 |
| Marshall Islands | 0.4 (0.2 to 0.7) | 4.2 (2.2 to 7.1) | 0.3 (0.2 to 0.6) | 2.3 (1.2 to 4) | -1.9(-1.97 to -1.82) | <0.001 |
| Mauritania | 460 (226 to 858) | 102.2 (50.1 to 190.8) | 694.5 (333.9 to 1292.6) | 67.8 (32.4 to 126.5) | -1.31(-1.38 to -1.24) | <0.001 |
| Mauritius | 86.3 (41.8 to 151.2) | 28.7 (13.9 to 50.4) | 24.1 (10.3 to 48.7) | 7.5 (3.2 to 15.1) | -4.26(-4.38 to -4.14) | <0.001 |
| Mexico | 3706.7 (1859.5 to 6814.3) | 17 (8.6 to 31.3) | 5677.2 (2595.4 to 10633.7) | 16.2 (7.4 to 30.3) | -0.18(-0.27 to -0.09) | <0.001 |
| Micronesia (Federated States of) | 0.9 (0.5 to 1.6) | 4.2 (2.2 to 7.2) | 0.6 (0.3 to 1.1) | 2.4 (1.2 to 4.2) | -1.84(-1.94 to -1.74) | <0.001 |
| Monaco | 0.9 (0.4 to 1.8) | 12.2 (5.3 to 23.9) | 0.9 (0.4 to 1.9) | 12.7 (5.5 to 25.1) | 0.14(0.11 to 0.17) | <0.001 |
| Mongolia | 169.7 (81.3 to 300.5) | 32.4 (15.6 to 57.3) | 112.9 (45.3 to 215.5) | 13.5 (5.4 to 25.7) | -2.84(-3.09 to -2.6) | <0.001 |
| Montenegro | 7 (2.8 to 14.1) | 4.5 (1.8 to 9) | 4.8 (1.9 to 9.7) | 3.3 (1.3 to 6.7) | -0.99(-1.09 to -0.88) | <0.001 |
| Morocco | 4557.7 (2467.5 to 7851.7) | 71.7 (39 to 123.5) | 3841.6 (1931.7 to 6442.5) | 39.7 (20 to 66.6) | -1.9(-1.97 to -1.84) | <0.001 |
| Mozambique | 1908.2 (995.1 to 3345.4) | 61.7 (32.1 to 108.4) | 1988 (931.9 to 3633) | 27 (12.8 to 49.4) | -2.69(-2.82 to -2.56) | <0.001 |
| Myanmar | 5850.6 (3134.3 to 9446.8) | 56.9 (30.5 to 91.8) | 2730.2 (1201 to 4893.9) | 18.2 (8 to 32.5) | -3.68(-4 to -3.35) | <0.001 |
| Namibia | 61.8 (27.9 to 116.6) | 18.7 (8.6 to 35.3) | 83.7 (36.2 to 161.7) | 12.8 (5.6 to 24.7) | -1.2(-1.33 to -1.06) | <0.001 |
| Nauru | 0.1 (0 to 0.1) | 2.4 (1.1 to 4.2) | 0 (0 to 0.1) | 0.9 (0.3 to 1.9) | -3.19(-3.42 to -2.95) | <0.001 |
| Nepal | 2411.6 (1292.4 to 3983.7) | 53.6 (28.8 to 88.6) | 2029.2 (900.9 to 3978.1) | 22.7 (10.1 to 44.6) | -2.71(-3.12 to -2.3) | <0.001 |
| Netherlands | 559.4 (240.6 to 1110.5) | 13.9 (6 to 27.5) | 502.1 (216.3 to 986.5) | 13.4 (5.8 to 26.3) | -0.13(-0.15 to -0.11) | <0.001 |
| New Zealand | 34.7 (14.1 to 70.4) | 3.8 (1.6 to 7.8) | 44.7 (18.6 to 91.3) | 3.7 (1.5 to 7.5) | -0.12(-0.17 to -0.07) | <0.001 |
| Nicaragua | 166.5 (71.4 to 307.9) | 18.5 (8 to 34.2) | 310 (132.5 to 586.1) | 17.1 (7.3 to 32.3) | -0.31(-0.61 to -0.01) | 0.04 |
| Niger | 1237.1 (634.5 to 2262.8) | 72.6 (36.9 to 133.7) | 3257 (1641.9 to 5924.2) | 65.2 (32.9 to 119.5) | -0.38(-0.49 to -0.28) | <0.001 |
| Nigeria | 6425.4 (3160.1 to 11835.5) | 33.5 (16.4 to 62) | 12269.6 (5425.4 to 23512.1) | 22.6 (10.1 to 43.4) | -1.31(-1.66 to -0.97) | <0.001 |
| Niue | 0 (0 to 0) | 2.8 (1.4 to 4.9) | 0 (0 to 0) | 0.8 (0.3 to 1.9) | -3.78(-3.99 to -3.57) | <0.001 |
| North Macedonia | 24.6 (9.9 to 47.9) | 4.8 (2 to 9.4) | 18.3 (7.3 to 36.2) | 3.4 (1.4 to 6.8) | -1.15(-1.32 to -0.98) | <0.001 |
| Northern Mariana Islands | 0.1 (0 to 0.3) | 1 (0.3 to 2.2) | 0.1 (0 to 0.2) | 0.8 (0.3 to 1.7) | -0.82(-0.89 to -0.74) | <0.001 |
| Norway | 158.7 (69.8 to 311.5) | 14.8 (6.5 to 29.1) | 181.4 (80.1 to 353.5) | 14.6 (6.4 to 28.3) | -0.06(-0.07 to -0.05) | <0.001 |
| Oman | 57.6 (24.6 to 104.4) | 17 (7.3 to 30.7) | 131.7 (51.4 to 248.4) | 13 (5 to 24.5) | -0.87(-0.96 to -0.77) | <0.001 |
| Pakistan | 35852.3 (18982.7 to 63603.4) | 158.1(83.2 to 281.7) | 114276.5 (58999.5 to 203134.8) | 191.6 (98.8 to 341.2) | 0.63 (0.57 to 0.69) | <0.001 |
| Palau | 0.1 (0.1 to 0.2) | 2.6 (1.3 to 4.5) | 0 (0 to 0.1) | 0.8 (0.3 to 1.9) | -3.63(-3.83 to -3.43) | <0.001 |
| Palestine | 93.2 (41.2 to 171.1) | 21.1 (9.4 to 38.6) | 87 (35.5 to 169.4) | 6.7 (2.7 to 13.1) | -3.66(-4.11 to -3.2) | <0.001 |
| Panama | 50.3 (16.7 to 99.2) | 8.2 (2.7 to 16.2) | 35.7 (14.3 to 71.7) | 3.3 (1.3 to 6.7) | -2.91(-3.05 to -2.76) | <0.001 |
| Papua New Guinea | 41.4 (21.9 to 71.6) | 4.4 (2.4 to 7.6) | 61.2 (31.1 to 107.6) | 2.4 (1.2 to 4.2) | -2.03(-2.15 to -1.91) | <0.001 |
| Paraguay | 44 (19.5 to 75.9) | 4.8 (2.1 to 8.2) | 33.7 (12.6 to 70.2) | 1.8 (0.7 to 3.7) | -3.19(-3.46 to -2.93) | <0.001 |
| Peru | 142.7 (69.9 to 252) | 2.7 (1.3 to 4.7) | 103.7 (35.4 to 225.6) | 1.1 (0.4 to 2.3) | -3.02(-3.16 to -2.87) | <0.001 |
| Philippines | 6792.4 (3778.7 to 11394.2) | 44.5 (24.8 to 74.7) | 16534.8 (8761.8 to 29077) | 57 (30.2 to 100.5) | 0.79(0.37 to 1.21) | <0.001 |
| Poland | 454.9 (216 to 828.4) | 4.8 (2.3 to 8.8) | 302 (124.9 to 601) | 3.4 (1.4 to 6.7) | -1.17(-1.27 to -1.06) | <0.001 |
| Portugal | 492.5 (220.9 to 954.2) | 19.5 (8.7 to 37.7) | 463.7 (202.7 to 903.4) | 19.1 (8.3 to 37) | -0.07(-0.16 to 0.03) | 0.176 |
| Puerto Rico | 67 (27.8 to 124.2) | 7 (2.9 to 13) | 27.1 (9.7 to 51.9) | 3.6 (1.3 to 6.9) | -2.17(-2.25 to -2.1) | <0.001 |
| Qatar | 5.6 (2.3 to 11) | 7.1 (2.9 to 14) | 30.8 (12.6 to 60.7) | 5.6 (2.3 to 11) | -0.81(-0.9 to -0.71) | <0.001 |
| Republic of Korea | 665.4 (279.2 to 1317) | 5.3 (2.2 to 10.5) | 437.8 (172.4 to 871.2) | 3.7 (1.5 to 7.3) | -1.14(-1.19 to -1.1) | <0.001 |
| Republic of Moldova | 26.9 (12.9 to 45.6) | 2.4 (1.1 to 4.1) | 25.6 (12.7 to 43.6) | 2.9 (1.4 to 5) | 0.65(0.54 to 0.76) | <0.001 |
| Romania | 421.4 (187.1 to 806.6) | 7.5 (3.3 to 14.3) | 190.3 (78.8 to 374.9) | 4.7 (1.9 to 9.1) | -1.52(-1.63 to -1.42) | <0.001 |
| Russian Federation | 2382.5 (1073.1 to 4179.4) | 6.4 (2.9 to 11.2) | 2276.9 (991.2 to 3952.2) | 6.7 (2.9 to 11.8) | 0.18(0.07 to 0.3) | 0.002 |
| Rwanda | 1142.7 (521.3 to 2165.4) | 74 (33.9 to 140.2) | 1794.2 (793.2 to 3519.1) | 52.9 (23.4 to 103.7) | -1.11(-1.22 to -0.99) | <0.001 |
| Saint Kitts and Nevis | 1.1 (0.4 to 2.2) | 11.1 (4.3 to 22.1) | 0.8 (0.3 to 1.4) | 4.9 (1.9 to 9) | -2.63(-2.7 to -2.56) | <0.001 |
| Saint Lucia | 2.2 (0.9 to 3.9) | 6.4 (2.6 to 11.6) | 1.9 (0.8 to 3.3) | 4.1 (1.8 to 7.1) | -1.4(-1.46 to -1.34) | <0.001 |
| Saint Vincent  and the Grenadines | 4.5 (1.9 to 8.3) | 17.3 (7.5 to 31.8) | 2.2 (0.8 to 4.4) | 7.9 (2.8 to 15.8) | -2.49(-2.58 to -2.39) | <0.001 |
| Samoa | 0.3 (0.1 to 0.7) | 0.9 (0.3 to 2) | 0.3 (0.1 to 0.8) | 0.7 (0.2 to 1.6) | -1(-1.09 to -0.92) | <0.001 |
| San Marino | 0.8 (0.4 to 1.6) | 13.4 (5.7 to 26.1) | 1 (0.4 to 2) | 13.9 (6 to 27.1) | 0.1(0.07 to 0.13) | <0.001 |
| Sao Tome and Principe | 6.8 (3.1 to 12.3) | 27.4 (12.7 to 49.5) | 11.5 (5.3 to 21.6) | 20.9 (9.7 to 39.3) | -0.89(-0.96 to -0.82) | <0.001 |
| Saudi Arabia | 219.8 (74.9 to 434) | 6.7 (2.2 to 13.4) | 533.6 (256.2 to 875.8) | 5.3 (2.5 to 8.7) | -0.78(-0.91 to -0.65) | <0.001 |
| Senegal | 943.4 (476.4 to 1669.2) | 57.3 (28.9 to 101.4) | 1339.6 (656.5 to 2440.7) | 35.3 (17.4 to 64.6) | -1.57(-1.67 to -1.47) | <0.001 |
| Serbia | 67.1 (26.2 to 136.1) | 2.9 (1.1 to 5.8) | 40.4 (15.1 to 83) | 2 (0.7 to 4.1) | -1.29(-1.71 to -0.87) | <0.001 |
| Seychelles | 4 (1.8 to 7.1) | 22.7 (10.2 to 40.1) | 1.7 (0.7 to 3.3) | 6.8 (2.9 to 13.5) | -3.87(-3.96 to -3.77) | <0.001 |
| Sierra Leone | 379.3 (186.8 to 674.4) | 38.6 (19 to 68.6) | 743.9 (358.8 to 1331.2) | 33.6 (16.3 to 60.1) | -0.47(-0.54 to -0.41) | <0.001 |
| Singapore | 41.4 (16.8 to 84.1) | 4.4 (1.8 to 9) | 55.9 (22.3 to 110.9) | 3.7 (1.5 to 7.4) | -0.53(-0.56 to -0.5) | <0.001 |
| Slovakia | 60.7 (24.4 to 122) | 4.6 (1.8 to 9.2) | 39.5 (15.6 to 79.1) | 3.1 (1.2 to 6.2) | -1.24(-1.29 to -1.19) | <0.001 |
| Slovenia | 21.6 (8.9 to 42.8) | 4.3 (1.8 to 8.6) | 13.1 (5.3 to 26.6) | 3.1 (1.2 to 6.3) | -1.09(-1.14 to -1.04) | <0.001 |
| Solomon Islands | 2 (1 to 3.5) | 2.8 (1.5 to 4.9) | 3.7 (1.9 to 6.6) | 2.2 (1.1 to 3.9) | -0.81(-0.87 to -0.75) | <0.001 |
| South Africa | 1442.2 (718.4 to 2674.1) | 15.2 (7.6 to 28.1) | 1739.9 (829.8 to 3315.4) | 11.2 (5.3 to 21.3) | -1.03(-1.14 to -0.92) | <0.001 |
| Somalia | 5534.2 (2820.7 to 10021.1) | 340.1 (173 to 616.9) | 16690.7 (8269.3 to 29889.6) | 367.4 (181.2 to 659.1) | 0.25(0.22 to 0.28) | <0.001 |
| South Sudan | 412 (198.4 to 749.3) | 33.3 (16.1 to 60.7) | 868.5 (429.8 to 1569) | 38.8 (19.3 to 70.3) | 0.48(0.36 to 0.59) | <0.001 |
| Spain | 1412.5 (611.5 to 2772.7) | 14.7 (6.4 to 28.9) | 1473.2 (641.8 to 2910.8) | 14.2 (6.1 to 28) | -0.12(-0.18 to -0.06) | <0.001 |
| Sri Lanka | 3158.1 (1684.8 to 5421.6) | 69.3 (37 to 119.1) | 1169.6 (511.2 to 2306.5) | 20.5 (9 to 40.4) | -3.9(-4.1 to -3.7) | <0.001 |
| Sudan | 3516.8 (1918.3 to 5638.3) | 74.6 (41 to 119.5) | 4366.1 (2159.6 to 7436.5) | 38.6 (19.2 to 65.8) | -2.16(-2.36 to -1.95) | <0.001 |
| Suriname | 12.4 (4.9 to 23.7) | 12.9 (5.1 to 24.6) | 10.2 (4 to 19) | 7 (2.7 to 13) | -1.98(-2.17 to -1.79) | <0.001 |
| Sweden | 330.1 (142.5 to 649.1) | 15.7 (6.8 to 30.9) | 345.7 (152.8 to 689.4) | 15.3 (6.7 to 30.3) | -0.11(-0.13 to -0.09) | <0.001 |
| Switzerland | 144.2 (62 to 285.4) | 8 (3.4 to 15.8) | 164 (70.9 to 321.5) | 8 (3.5 to 15.8) | 0.01(-0.06 to 0.08) | 0.786 |
| Syrian Arab Republic | 1294.2 (667.2 to 2173.9) | 46.9 (24.3 to 78.6) | 1447.5 (719.7 to 2523.6) | 37.2 (18.5 to 64.6) | -0.75(-0.91 to -0.58) | <0.001 |
| Taiwan (Province of China) | 141.8 (56.6 to 292.7) | 2.6 (1 to 5.3) | 97.2 (37.7 to 208.1) | 1.6 (0.6 to 3.5) | -1.49(-1.57 to -1.41) | <0.001 |
| Tajikistan | 327.2 (149.2 to 580.9) | 26.4 (12.1 to 47) | 629.6 (282.9 to 1106.2) | 24.6 (11.1 to 43.2) | -0.23(-0.37 to -0.09) | 0.002 |
| Thailand | 6575.2 (3395.4 to 11375.2) | 41.5 (21.4 to 71.9) | 2839.6 (1233.5 to 5307.7) | 17 (7.3 to 31.9) | -2.85(-3.28 to -2.42) | <0.001 |
| Timor-Leste | 65.7 (33.4 to 112) | 35.6 (18.1 to 60.7) | 53.5 (22.2 to 98.9) | 16.3 (6.9 to 30) | -2.46(-2.75 to -2.17) | <0.001 |
| Togo | 576.9 (300 to 1011.7) | 70.2 (36.3 to 123.2) | 1006.7 (501.5 to 1806.3) | 47.4 (23.7 to 85.1) | -1.31(-1.43 to -1.19) | <0.001 |
| Tokelau | 0 (0 to 0) | 3.7 (2 to 6.4) | 0 (0 to 0) | 1.9 (0.9 to 3.4) | -2.12(-2.19 to -2.05) | <0.001 |
| Tonga | 0.8 (0.4 to 1.3) | 3.7 (1.9 to 6.3) | 0.5 (0.3 to 0.9) | 2.1 (1 to 3.8) | -1.82(-1.9 to -1.74) | <0.001 |
| Trinidad and Tobago | 55.2 (25.2 to 104.4) | 18 (8.2 to 34) | 26.9 (11.7 to 52.4) | 7.8 (3.4 to 15.1) | -2.74(-2.93 to -2.55) | <0.001 |
| Tunisia | 326.4 (141.6 to 600.5) | 16 (7 to 29.5) | 311.7 (111.6 to 594.7) | 10.2 (3.6 to 19.4) | -1.44(-1.6 to -1.27) | <0.001 |
| Turkey | 7611.8 (3929.3 to 13066.1) | 53.2 (27.5 to 91.3) | 5980.7 (2801.8 to 10651.4) | 27.6 (12.9 to 49.1) | -2.13(-2.24 to -2.01) | <0.001 |
| Turkmenistan | 75.4 (31 to 142.1) | 8.3 (3.4 to 15.7) | 85.4 (35.6 to 168.1) | 6.8 (2.8 to 13.4) | -0.65(-0.73 to -0.58) | <0.001 |
| Tuvalu | 0.1 (0.1 to 0.2) | 5.2 (2.8 to 9.1) | 0.1 (0 to 0.1) | 2.3 (1.2 to 4) | -2.61(-2.66 to -2.56) | <0.001 |
| Uganda | 1417.2 (688.9 to 2524.3) | 38.5 (19 to 68.5) | 1596.6 (685.2 to 3153.6) | 16.2 (7 to 32) | -2.77(-3.13 to -2.41) | <0.001 |
| Ukraine | 1895.5 (808.2 to 3509.1) | 15 (6.4 to 27.8) | 1704.9 (748.8 to 3179.6) | 17.1 (7.5 to 32) | 0.46(0.23 to 0.69) | <0.001 |
| United Arab Emirates | 74.4 (31.9 to 142) | 21.7 (9.4 to 41.4) | 279.4 (118.2 to 536.6) | 16.1 (6.7 to 31) | -0.95(-1.06 to -0.85) | <0.001 |
| United Kingdom | 2099.4 (929.4 to 4098.9) | 14.5 (6.4 to 28.4) | 2264.2 (1001 to 4416.4) | 14.3 (6.3 to 27.8) | -0.06(-0.07 to -0.04) | <0.001 |
| United Republic  of Tanzania | 1083.8 (465.7 to 1953.3) | 18.7 (8.1 to 33.6) | 1939.8 (746.2 to 3711.1) | 13.3 (5.2 to 25.6) | -1.12(-1.21 to -1.03) | <0.001 |
| United States  of America | 2486.8 (1031.7 to 4895.8) | 3.7 (1.5 to 7.2) | 2759.3 (1145.6 to 5473.5) | 3.6 (1.5 to 7.2) | -2.69(-2.78 to -2.61) | <0.001 |
| United States  Virgin Islands | 2.1 (0.9 to 3.9) | 7.4 (3.1 to 13.8) | 0.6 (0.2 to 1.1) | 3.2 (1.1 to 6.3) | -0.05(-0.06 to -0.03) | <0.001 |
| Uruguay | 19 (7.4 to 38.7) | 2.5 (1 to 5.2) | 17.1 (6.5 to 34.8) | 2.1 (0.8 to 4.2) | -0.65(-0.71 to -0.59) | <0.001 |
| Uzbekistan | 1739.9 (852.1 to 3056.7) | 34.7 (17 to 61.1) | 1782.5 (800 to 3223.9) | 20.1 (9 to 36.3) | -1.78(-1.9 to -1.65) | <0.001 |
| Vanuatu | 7.6 (3.6 to 13.2) | 22.1 (10.5 to 38.6) | 14.2 (6.3 to 25.5) | 18.5 (8.3 to 33.1) | -0.58(-0.67 to -0.48) | <0.001 |
| Venezuela (Bolivarian Republic of) | 345.5 (144.5 to 682.9) | 7.1 (3 to 14.1) | 816.1 (336.8 to 1550.1) | 11.8 (4.9 to 22.4) | 1.42(0.87 to 1.97) | <0.001 |
| Viet Nam | 13266.5 (7070.6 to 22889.9) | 80.1 (42.8 to 139.2) | 7052.1 (3363.8 to 12368) | 26.8 (12.7 to 47.1) | -3.55(-3.91 to -3.18) | <0.001 |
| Yemen | 1762.6 (911 to 2948.3) | 63.8 (33.2 to 106.8) | 5883.1 (3092.7 to 9881) | 69.7 (36.7 to 117) | 0.28(0.15 to 0.42) | <0.001 |
| Zambia | 638.8 (316.5 to 1155.3) | 36.6 (18.4 to 66.5) | 1205.3 (562.6 to 2198.3) | 25.2 (11.8 to 46.1) | -1.23(-1.34 to -1.11) | <0.001 |
| Zimbabwe | 1459.1 (693.3 to 2681.3) | 63.7 (30.3 to 116.9) | 1843.7 (828.7 to 3565.4) | 47 (21.2 to 91) | -1.01(-1.11 to -0.91) | <0.001 |

Estimates are for individuals aged 15–49 years. Rates are reported per 100000 populations. ASIR: age-standardized incidence rate; ASPR: age-standardized prevalence rate; ASYR: age-standardized YLD rate; SDI: socio-demographic index; AAPC: average annual percent change.

# Supplementary Table 2 Age-specific AAPC of iodine deficiency burden from 1990 to 2021 globally and by SDI categories.

| **Age** | | **Global** | | | | | | | **High SDI** | | | |
| --- | --- | --- | --- | --- | --- | --- | --- | --- | --- | --- | --- | --- |
|  |  | **Rate in 1990 (95% UI)** | **Rate in 2021 (95% UI)** | | | **AAPC (95% CI)** | **P** | | **Rate in 2009 (95% UI)** | **Rate in 2021 (95% UI)** | **AAPC (95% CI)** | **P** |
|  |  |  |  |  |  |  |  |  |  |  |  |  |
| **Age specific incidence rate** | | | | | | | | | | | | |
| 15 to 19 | | 345.1(264.7 to 443.3) | 373.3(280.6 to 478.7) | | | 0.23(0.14 to 0.32) | <0.001 | | 17.8(8.1 to 443.1) | 45.4(30.9 to 62.6) | -0.1(-0.14 to -0.05) | <0.001 |
| 20 to 24 | | 305.6(210.3 to 443.3) | 364.4(263.4 to 477.5) | | | 0.54(0.39 to 0.68) | <0.001 | | 16.1(9.3 to 443.1) | 44.8(27.0 to 66.3) | -0.16(-0.25 to -0.07) | <0.001 |
| 25 to 29 | | 212.2(149.1 to 443.2) | 247.0(182.2 to 318.9) | | | 0.45(0.33 to 0.57) | <0.001 | | 46.9(31.7 to 443.4) | 37.2(24.7 to 51.8) | -0.21(-0.27 to -0.14) | <0.001 |
| 30 to 34 | | 131.4(96.3 to 443.1) | 147.9(111.1 to 187.3) | | | 0.36(0.26 to 0.45) | <0.001 | | 47.4(28.0 to 443.4) | 30.3(21.0 to 41.0) | -0.04(-0.09 to 0.01) | 0.111 |
| 35 to 39 | | 59.4(41.9 to 443.5) | 64.2(46.1 to 85.7) | | | 0.24(0.2 to 0.28) | <0.001 | | 39.6(25.5 to 443.3) | 21.8(12.4 to 34.9) | -0.1(-0.12 to -0.07) | <0.001 |
| 40 to 44 | | 23.1(9.8 to 443.2) | 23.0(9.8 to 45.0) | | | 0.01(-0.06 to 0.08) | 0.735 | | 30.7(20.7 to 443.3) | 16.8(7.8 to 29.9) | -0.18(-0.22 to -0.15) | <0.001 |
| 45 to 49 | | 20.4(11.1 to 443.2) | 19.6(10.8 to 33.7) | | | -0.13(-0.17 to -0.09) | <0.001 | | 22.5(12.6 to 443.2) | 15.0(8.7 to 23.7) | -0.23(-0.27 to -0.18) | <0.001 |
|  | | **High-middle SDI** | | | | | | | **Middle SDI** | | | |
| 15 to 19 | | 183.8(141.5 to 443.1) | 173.4(131.2 to 221.0) | | | -0.24(-0.36 to -0.11) | <0.001 | | 242.9(180.2 to 443.2) | 259.0(195.9 to 333.9) | 0.2(0.13 to 0.27) | <0.001 |
| 20 to 24 | | 183.2(127.5 to 443.1) | 184.0(132.5 to 240.5) | | | -0.01(-0.11 to 0.09) | 0.849 | | 234.8(158.3 to 443.2) | 273.6(198.0 to 359.1) | 0.48(0.37 to 0.6) | <0.001 |
| 25 to 29 | | 131.8(93.6 to 443.1) | 135.3(99.7 to 174.6) | | | 0.06(-0.06 to 0.17) | 0.339 | | 173.2(120.0 to 443.1) | 199.7(148.4 to 257.5) | 0.45(0.34 to 0.56) | <0.001 |
| 30 to 34 | | 82.6(61.4 to 443.8) | 94.7(71.8 to 120.0) | | | 0.45(0.29 to 0.62) | <0.001 | | 113.0(82.2 to 443.1) | 126.0(96.1 to 159.4) | 0.35(0.27 to 0.43) | <0.001 |
| 35 to 39 | | 41.2(27.7 to 443.4) | 42.8(29.6 to 59.7) | | | 0.09(-0.05 to 0.22) | 0.205 | | 53.5(36.7 to 443.5) | 55.1(39.7 to 74.0) | 0.1(0.02 to 0.19) | 0.02 |
| 40 to 44 | | 19.6(8.2 to 443.1) | 17.8(7.6 to 34.6) | | | -0.3(-0.39 to -0.2) | <0.001 | | 22.9(9.6 to 443.2) | 20.1(8.5 to 39.4) | -0.41(-0.47 to -0.36) | <0.001 |
| 45 to 49 | | 17.5(9.4 to 443.1) | 16.0(8.8 to 27.0) | | | -0.31(-0.53 to -0.1) | 0.004 | | 20.4(11.0 to 443.2) | 17.5(9.5 to 30.3) | -0.48(-0.52 to -0.43) | <0.001 |
|  | | **Low-middle SDI** | | | | | | | **Low SDI** | | | |
| 15 to 19 | | 603.2(466.9 to 443.6) | 474.2(356.2 to 606.2) | | | -3.17(-3.31 to -3.03) | <0.001 | | 800.7(628.0 to 443.8) | 655.7(491.9 to 847.5) | -0.65(-0.77 to -0.53) | <0.001 |
| 20 to 24 | | 534.8(365.6 to 443.5) | 486.2(350.8 to 635.2) | | | -2.57(-2.75 to -2.39) | <0.001 | | 715.9(521.8 to 443.7) | 626.5(446.4 to 824.3) | -0.43(-0.57 to -0.29) | <0.001 |
| 25 to 29 | | 380.3(263.9 to 443.3) | 343.8(255.4 to 444.8) | | | -2.25(-2.39 to -2.11) | <0.001 | | 496.2(373.6 to 443.4) | 433.7(316.2 to 559.8) | -0.45(-0.57 to -0.34) | <0.001 |
| 30 to 34 | | 240.0(171.6 to 443.2) | 213.1(158.9 to 269.7) | | | -2.2(-2.33 to -2.06) | <0.001 | | 306.8(235.0 to 443.3) | 269.2(200.8 to 339.9) | -0.45(-0.6 to -0.29) | <0.001 |
| 35 to 39 | | 103.2(75.5 to 443.1) | 92.0(67.4 to 121.0) | | | -2.22(-2.37 to -2.08) | <0.001 | | 128.4(96.5 to 443.1) | 116.0(85.7 to 148.9) | -0.33(-0.37 to -0.3) | <0.001 |
| 40 to 44 | | 29.9(12.5 to 443.2) | 30.1(12.7 to 60.2) | | | -2.27(-2.38 to -2.16) | <0.001 | | 33.5(13.7 to 443.3) | 34.9(14.7 to 69.0) | 0.11(0.06 to 0.16) | <0.001 |
| 45 to 49 | | 25.4(13.6 to 443.2) | 25.7(13.9 to 45.1) | | | -2.28(-2.4 to -2.17) | <0.001 | | 28.8(15.2 to 443.2) | 30.1(15.9 to 53.2) | 0.14(0.11 to 0.16) | <0.001 |
| **Age** | | **Global** | | | | | | | **High SDI** | | | |
|  |  | **Rate in 1990 (95% UI)** | **Rate in 2021 (95% UI)** | | | **AAPC (95% CI)** | | | **Rate in 2009 (95% UI)** | **Rate in 2021 (95% UI)** | **AAPC (95% CI)** | **P** |
| **Age specific prevalence rate** | | | | | | | | | | | | |
| 15 to 19 | 3341.3(2710.0 to 3943.1) | | | 2616.2(2112.8 to 3112.0) | -0.79(-0.9 to -0.68) | | | 451.7(318.2 to 595.8) | | 417.6(296.9 to 546.4) | -0.65(-0.77 to -0.53) | <0.001 |
| 20 to 24 | 4442.2(3667.7 to 5239.2) | | | 4063.3(3281.4 to 4937.9) | -0.3(-0.34 to -0.25) | | | 628.0(460.8 to 805.5) | | 569.9(425.1 to 727.2) | -0.43(-0.57 to -0.29) | <0.001 |
| 25 to 29 | 5095.7(4174.8 to 6125.4) | | | 4850.1(3821.8 to 5944.1) | -0.16(-0.2 to -0.12) | | | 744.5(538.3 to 959.7) | | 677.8(495.8 to 864.0) | -0.45(-0.57 to -0.34) | <0.001 |
| 30 to 34 | 5302.4(4310.2 to 6408.2) | | | 5003.1(3921.4 to 6082.3) | -0.21(-0.3 to -0.13) | | | 786.4(572.9 to 1018.4) | | 762.4(560.5 to 977.7) | -0.45(-0.6 to -0.29) | <0.001 |
| 35 to 39 | 5205.7(4193.6 to 6292.0) | | | 4921.4(3858.3 to 6047.5) | -0.18(-0.25 to -0.11) | | | 795.2(603.3 to 1031.5) | | 774.4(590.2 to 988.8) | -0.33(-0.37 to -0.3) | <0.001 |
| 40 to 44 | 4905.6(3978.0 to 5978.9) | | | 4557.8(3586.7 to 5673.3) | -0.23(-0.27 to -0.2) | | | 779.3(594.3 to 1017.8) | | 741.0(572.2 to 959.0) | 0.11(0.06 to 0.16) | <0.001 |
| 45 to 49 | 4695.5(3845.2 to 5752.5) | | | 4108.8(3220.2 to 5126.8) | -0.45(-0.52 to -0.38) | | | 761.9(576.4 to 1004.9) | | 717.6(550.6 to 937.9) | 0.14(0.11 to 0.16) | <0.001 |
| **High-middle SDI** | | | | | | | | **Middle SDI** | | | | |
| 15 to 19 | 1474.0(1148.9 to 1804.4) | | | 1072.1(862.9 to 1290.3) | -1.02(-1.14 to -0.91) | | | 2139.8(1673.5 to 2603.0) | | 1577.2(1261.9 to 1895.4) | -0.99(-1.06 to -0.91) | <0.001 |
| 20 to 24 | 2180.1(1740.4 to 2658.1) | | | 1825.5(1453.6 to 2237.8) | -0.58(-0.66 to -0.51) | | | 3058.2(2425.6 to 3717.7) | | 2735.4(2172.1 to 3352.1) | -0.36(-0.41 to -0.31) | <0.001 |
| 25 to 29 | 2620.8(2061.8 to 3234.4) | | | 2353.7(1832.0 to 2885.1) | -0.37(-0.47 to -0.27) | | | 3742.8(2945.4 to 4610.3) | | 3556.0(2777.9 to 4381.0) | -0.15(-0.22 to -0.08) | <0.001 |
| 30 to 34 | 2716.3(2123.2 to 3327.6) | | | 2817.4(2161.7 to 3479.9) | 0.13(-0.03 to 0.29) | | | 4057.6(3154.0 to 4996.5) | | 3901.9(3036.2 to 4817.8) | -0.13(-0.22 to -0.04) | 0.006 |
| 35 to 39 | 2717.6(2131.5 to 3352.6) | | | 2806.5(2166.1 to 3481.8) | 0.04(-0.15 to 0.23) | | | 3986.6(3107.1 to 4974.5) | | 3874.0(3002.9 to 4802.7) | -0.1(-0.16 to -0.04) | 0.001 |
| 40 to 44 | 2651.1(2109.6 to 3320.6) | | | 2627.4(2036.0 to 3310.3) | 0(-0.17 to 0.16) | | | 3846.0(3016.9 to 4837.2) | | 3645.3(2833.5 to 4534.8) | -0.19(-0.25 to -0.12) | <0.001 |
| 45 to 49 | 2493.0(1980.5 to 3123.5) | | | 2644.2(2050.2 to 3355.1) | 0.2(0.07 to 0.34) | | | 3625.8(2880.0 to 4570.7) | | 3472.0(2699.5 to 4347.7) | -0.13(-0.22 to -0.04) | 0.006 |
| **Low-middle SDI** | | | | | | | | **Low SDI** | | | | |
| 15 to 19 | 6465.8(5251.2 to 7595.5) | | | 3189.4(2576.0 to 3827.8) | -2.25(-2.31 to -2.2) | | | 7649.7(6510.3 to 8802.9) | | 5161.7(4186.9 to 6159.1) | -1.25(-1.36 to -1.14) | <0.001 |
| 20 to 24 | 8680.3(7243.3 to 10107.2) | | | 5231.9(4171.9 to 6399.0) | -1.63(-1.69 to -1.57) | | | 10834.2(9271.8 to 12491.9) | | 7928.3(6564.3 to 9500.6) | -1(-1.1 to -0.91) | <0.001 |
| 25 to 29 | 10090.7(8298.8 to 11961.2) | | | 6607.4(5155.7 to 8068.8) | -1.37(-1.41 to -1.32) | | | 13032.0(11120.9 to 15188.8) | | 9846.0(8020.0 to 11998.7) | -0.91(-0.99 to -0.83) | <0.001 |
| 30 to 34 | 10850.8(8835.1 to 13011.2) | | | 7216.9(5634.7 to 8828.4) | -1.31(-1.36 to -1.27) | | | 14235.2(11991.3 to 16674.6) | | 10855.5(8731.5 to 13234.7) | -0.89(-0.93 to -0.84) | <0.001 |
| 35 to 39 | 11149.2(9050.9 to 13448.3) | | | 7291.1(5686.7 to 8977.5) | -1.36(-1.4 to -1.31) | | | 14794.5(12433.5 to 17435.2) | | 11231.6(9004.5 to 13762.7) | -0.9(-0.94 to -0.85) | <0.001 |
| 40 to 44 | 10759.5(8764.0 to 12986.0) | | | 6942.7(5462.1 to 8615.9) | -1.41(-1.47 to -1.36) | | | 14139.2(11855.3 to 16714.6) | | 10904.9(8697.9 to 13240.9) | -0.84(-0.94 to -0.74) | <0.001 |
| 45 to 49 | 10074.3(8256.5 to 12278.8) | | | 6531.7(5107.7 to 8160.1) | -1.4(-1.47 to -1.33) | | | 13523.6(11285.4 to 16057.4) | | 10120.8(8108.6 to 12369.9) | -0.94(-1.04 to -0.83) | <0.001 |
| **Age** | | **Global** | | | | | | | **High SDI** | | | |
|  |  | **Rate in 1990 (95% UI)** | | **Rate in 2021 (95% UI)** | | **AAPC (95% CI)** | **P** | | **Rate in 2009 (95% UI)** | **Rate in 2021 (95% UI)** | **AAPC (95% CI)** | **P** |
|  |  |  |  |  |  |  |  |  |  |  |  |  |
| **Age specific YLD rate** | | | | | | | | | | | | |
| 15 to 19 | | 62.2(38.2 to 98.3) | | 37.2(20.9 to 63.4) | | -1.69(-1.89 to -1.49) | <0.001 | | 5.0(2.0 to 9.6) | 4.6(1.9 to 8.8) | -0.27(-0.31 to -0.23) | <0.001 |
| 20 to 24 | | 72.0(44.8 to 117.7) | | 51.9(27.1 to 90.1) | | -1.07(-1.17 to -0.98) | <0.001 | | 6.9(3.0 to 13.3) | 6.3(2.7 to 12.0) | -0.3(-0.4 to -0.2) | <0.001 |
| 25 to 29 | | 77.7(47.1 to 127.4) | | 58.9(29.6 to 103.2) | | -0.88(-1.05 to -0.71) | <0.001 | | 8.1(3.6 to 15.8) | 7.4(3.3 to 14.4) | -0.31(-0.38 to -0.24) | <0.001 |
| 30 to 34 | | 78.3(46.4 to 128.1) | | 59.2(29.7 to 104.6) | | -0.91(-1 to -0.82) | <0.001 | | 8.6(3.9 to 16.5) | 8.3(3.8 to 16.0) | -0.08(-0.14 to -0.03) | 0.003 |
| 35 to 39 | | 75.4(44.7 to 124.4) | | 57.7(29.2 to 104.3) | | -0.87(-1 to -0.74) | <0.001 | | 8.6(3.8 to 17.0) | 8.4(3.8 to 16.3) | -0.07(-0.13 to -0.01) | 0.022 |
| 40 to 44 | | 70.7(41.9 to 117.4) | | 53.1(27.0 to 98.0) | | -0.92(-0.98 to -0.86) | <0.001 | | 8.4(3.7 to 16.7) | 8.0(3.6 to 15.7) | -0.16(-0.22 to -0.09) | <0.001 |
| 45 to 49 | | 67.3(40.2 to 113.3) | | 47.2(24.1 to 88.6) | | -1.17(-1.29 to -1.05) | <0.001 | | 8.2(3.5 to 16.5) | 7.7(3.4 to 15.5) | -0.19(-0.24 to -0.15) | <0.001 |
| **High-middle SDI** | | | | | | | | | **Middle SDI** | | | |
| 15 to 19 | | 19.3(10.1 to 34.2) | | 13.7(7.0 to 24.6) | | -1.13(-1.21 to -1.05) | <0.001 | | 42.6(24.7 to 67.7) | 19.4(9.4 to 34.9) | -2.51(-2.82 to -2.19) | <0.001 |
| 20 to 24 | | 26.5(13.3 to 47.6) | | 21.8(10.8 to 39.6) | | -0.64(-0.7 to -0.59) | <0.001 | | 52.2(31.8 to 87.3) | 32.0(15.2 to 58.5) | -1.56(-1.69 to -1.43) | <0.001 |
| 25 to 29 | | 31.5(15.6 to 57.0) | | 27.4(13.3 to 49.9) | | -0.47(-0.58 to -0.37) | <0.001 | | 60.6(35.8 to 100.3) | 40.7(19.6 to 75.1) | -1.24(-1.36 to -1.11) | <0.001 |
| 30 to 34 | | 32.5(16.1 to 58.7) | | 32.2(15.5 to 59.8) | | -0.02(-0.17 to 0.12) | 0.749 | | 64.0(37.9 to 104.2) | 44.2(21.5 to 81.0) | -1.2(-1.28 to -1.11) | <0.001 |
| 35 to 39 | | 32.1(16.1 to 59.0) | | 32.0(15.4 to 59.6) | | -0.04(-0.18 to 0.1) | 0.571 | | 61.1(36.2 to 100.2) | 43.6(21.5 to 80.9) | -1.09(-1.23 to -0.95) | <0.001 |
| 40 to 44 | | 31.1(15.7 to 57.7) | | 29.9(14.6 to 56.3) | | -0.11(-0.27 to 0.05) | 0.181 | | 59.1(34.5 to 97.0) | 41.0(20.1 to 77.8) | -1.17(-1.28 to -1.05) | <0.001 |
| 45 to 49 | | 29.2(14.8 to 55.4) | | 29.6(14.5 to 57.4) | | 0.06(-0.07 to 0.19) | 0.351 | | 55.5(32.4 to 91.3) | 38.6(19.2 to 74.6) | -1.16(-1.25 to -1.07) | <0.001 |
| **Low-middle SDI** | | | | | | | | | **Low SDI** | | | |
| 15 to 19 | | 132.5(83.7 to 202.9) | | 49.0(29.2 to 81.1) | | -0.77(-0.91 to -0.63) | <0.001 | | 123.0(75.0 to 197.3) | 73.9(41.4 to 125.6) | -1.63(-1.69 to -1.57) | <0.001 |
| 20 to 24 | | 154.4(99.7 to 242.8) | | 69.9(37.6 to 121.3) | | -0.31(-0.49 to -0.14) | <0.001 | | 156.4(93.5 to 259.9) | 102.5(55.5 to 175.6) | -1.36(-1.45 to -1.28) | <0.001 |
| 25 to 29 | | 167.2(105.0 to 268.0) | | 82.9(42.6 to 144.7) | | -0.34(-0.5 to -0.17) | <0.001 | | 179.0(105.5 to 303.3) | 121.7(63.1 to 210.4) | -1.24(-1.29 to -1.2) | <0.001 |
| 30 to 34 | | 173.0(106.4 to 277.8) | | 87.7(45.1 to 154.7) | | -0.39(-0.52 to -0.25) | <0.001 | | 190.5(108.3 to 325.6) | 131.5(68.8 to 229.1) | -1.2(-1.27 to -1.14) | <0.001 |
| 35 to 39 | | 174.7(107.7 to 279.2) | | 87.3(44.2 to 155.6) | | -0.37(-0.47 to -0.27) | <0.001 | | 196.4(113.4 to 339.6) | 134.8(70.5 to 238.9) | -1.21(-1.27 to -1.16) | <0.001 |
| 40 to 44 | | 167.0(103.0 to 266.6) | | 82.5(43.0 to 150.9) | | 0.05(-0.04 to 0.14) | 0.235 | | 187.1(105.5 to 320.5) | 129.9(68.2 to 231.6) | -1.18(-1.29 to -1.08) | <0.001 |
| 45 to 49 | | 155.7(95.2 to 252.9) | | 76.8(40.0 to 143.1) | | 0.06(-0.01 to 0.12) | 0.108 | | 177.2(99.0 to 304.7) | 119.9(62.4 to 217.5) | -1.26(-1.36 to -1.15) | <0.001 |

SDI: socio-demographic index; AAPC: average annual percent change.


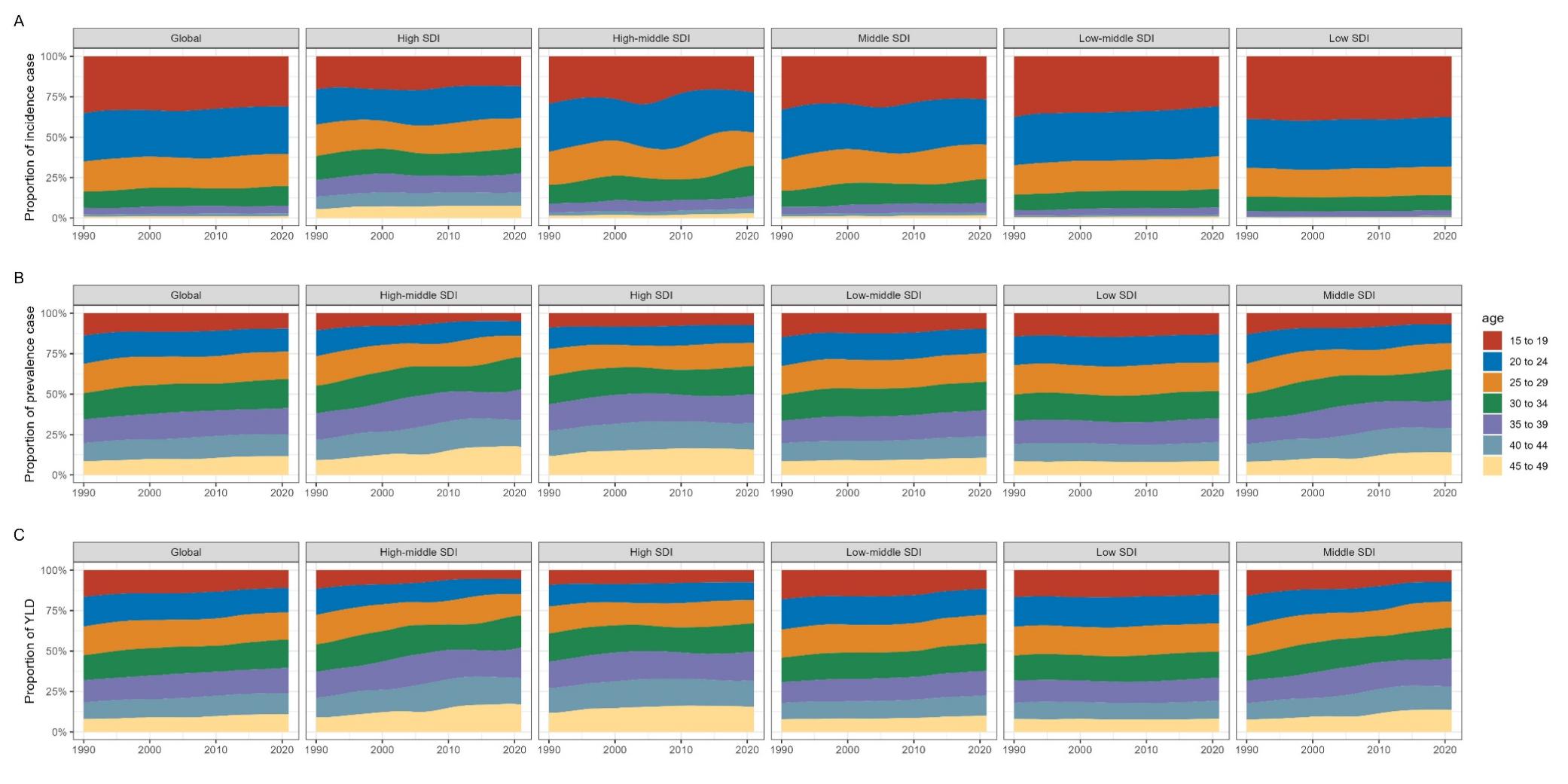


Supplementary Figure 1. The Proportion of cases in different age groups for iodine deficiency burden among WRA globally and by SDI categories from 1990 to 2021. (A) Proportion of incidence case; (B) Proportion of prevalence rate; (C) Proportion of YLD.


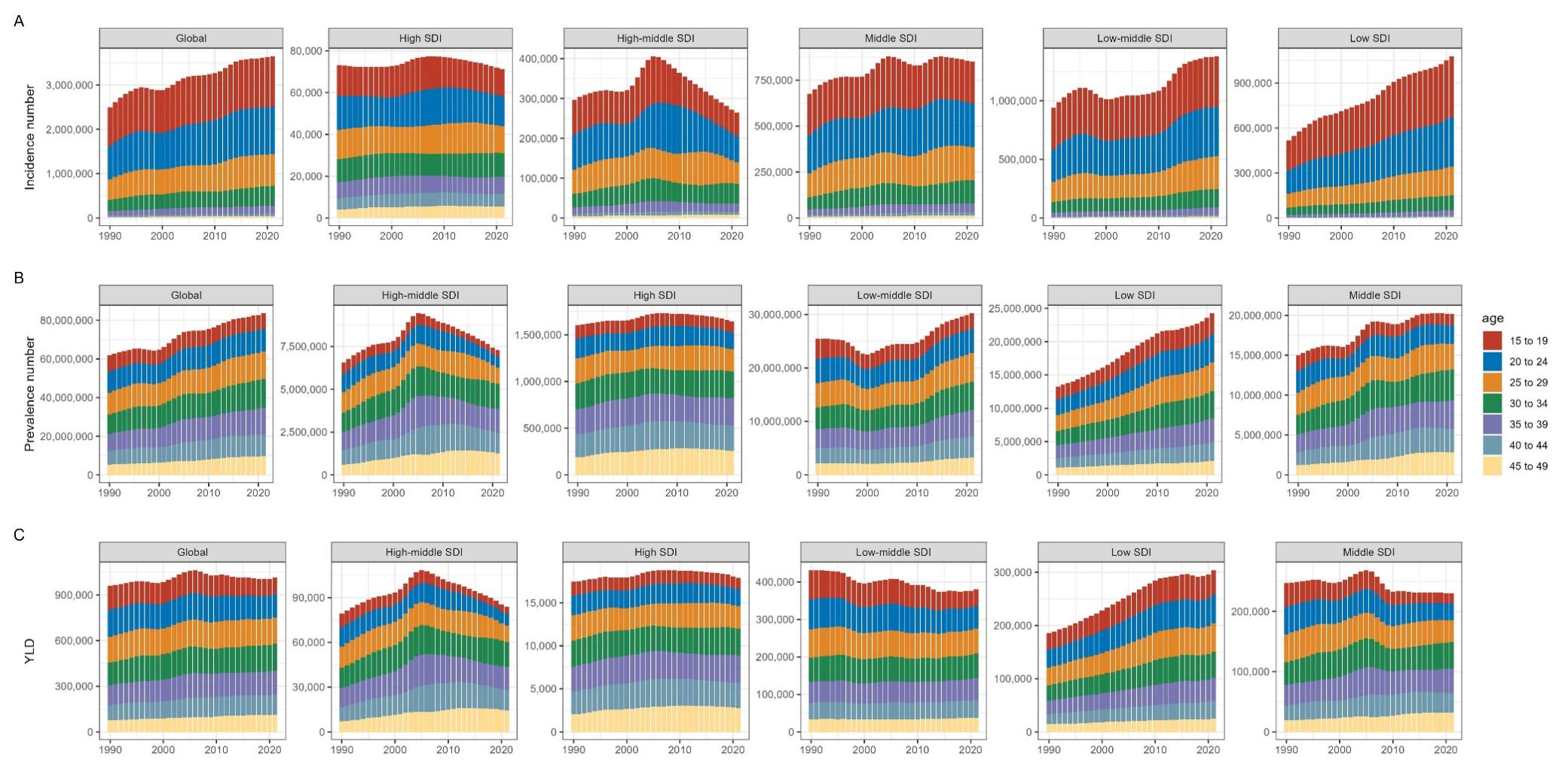


Supplementary Figure 2. The incidence number of iodine deficiency among WRA in different age groups globally and by SDI categories from 1990 to 2021. (A)Incidence number; (B)prevalence number; (C)YLD


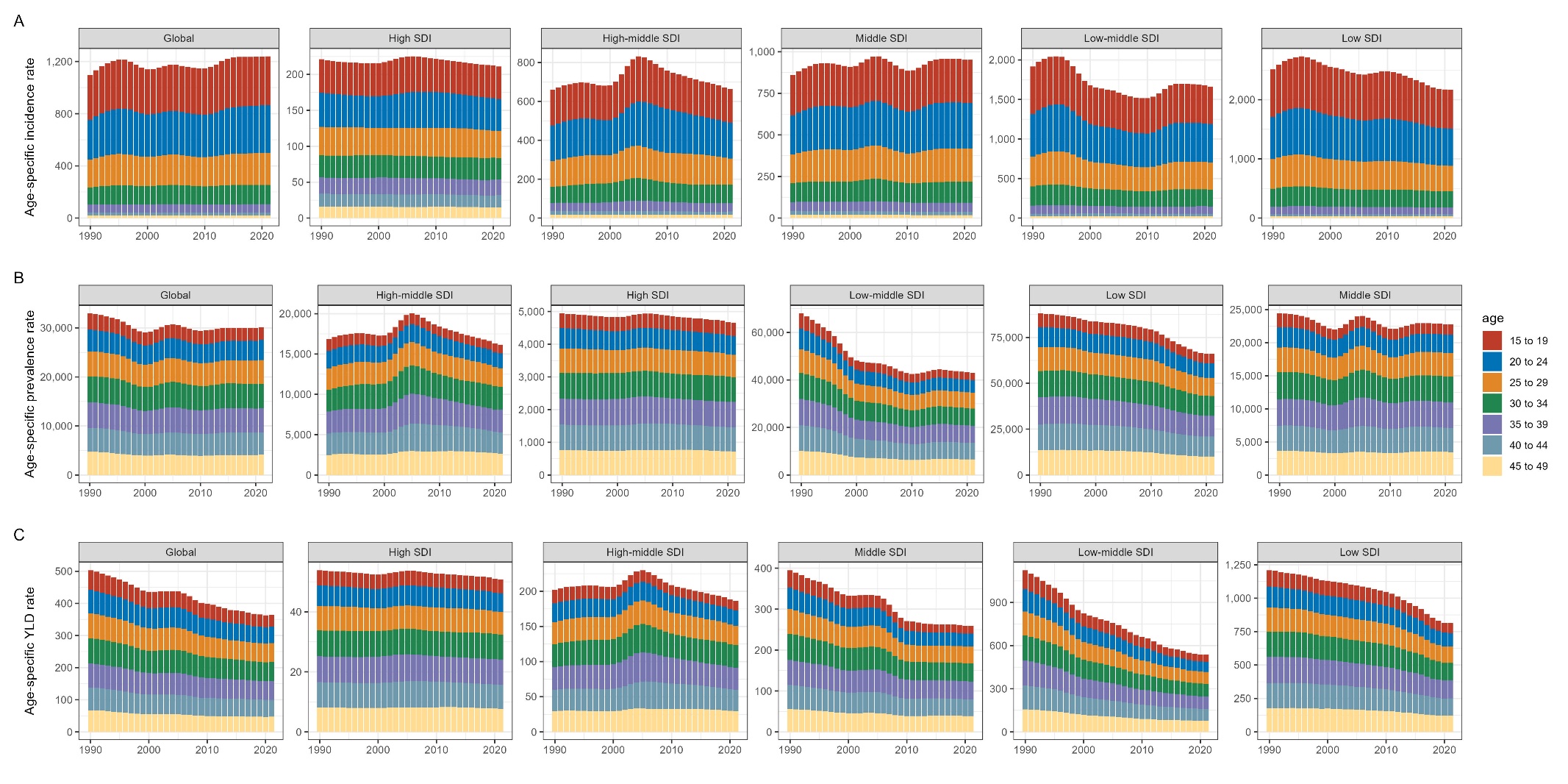


Supplementary Figure 3. The age specific rate of iodine deficiency burden among WRA globally and by SDI quintiles from 1990 to 2021. (A) Age-specific incidence rate; (B) Age-specific prevalence rate (C) Age-specific YLD rate.


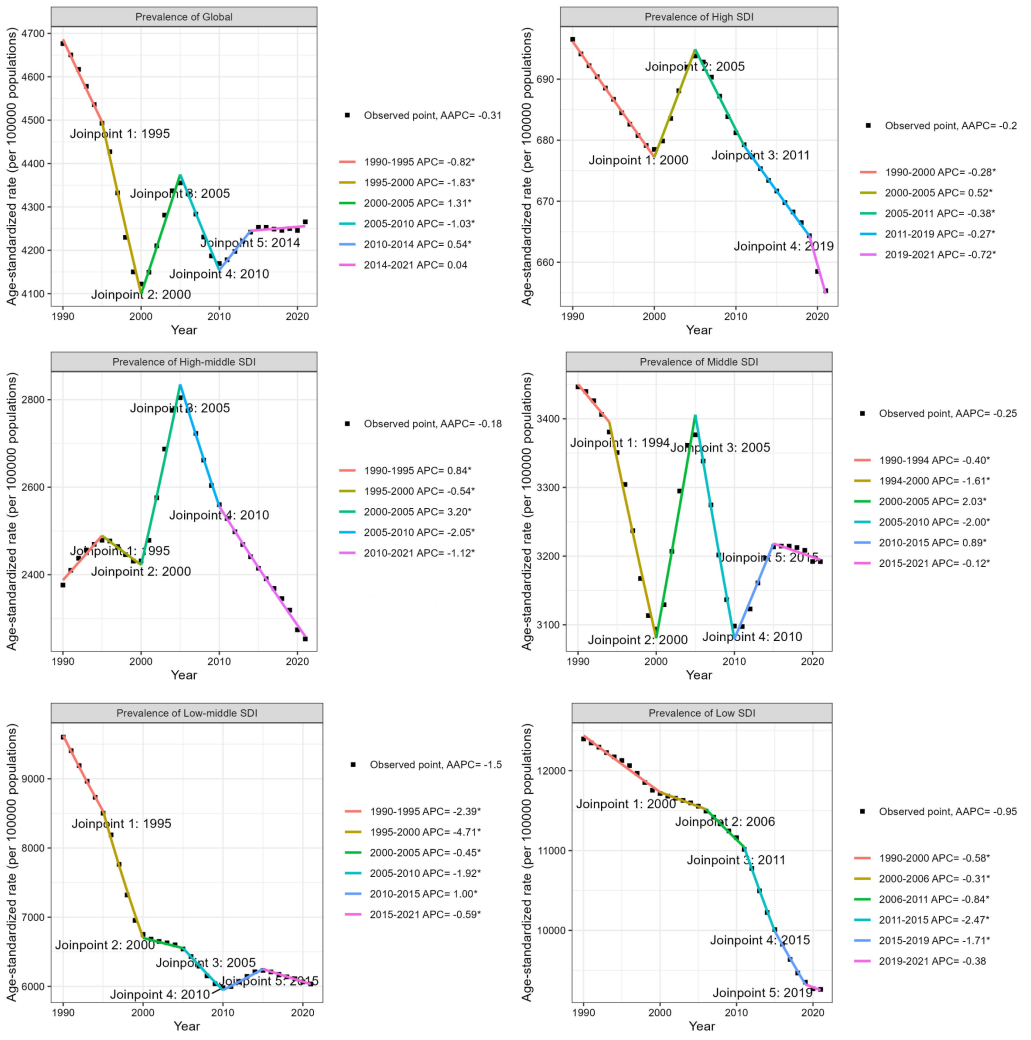


Supplementary Figure 4. Joinpoint regression analysis of temporal trends for age-standardized prevalence rate of iodine deficiency among WRA globally and by SDI quintiles from 1990 to 2021. Rates are expressed per 100,000 populations. APC: annual percent change; AAPC: average annual percent change; SDI: socio-demographic index.


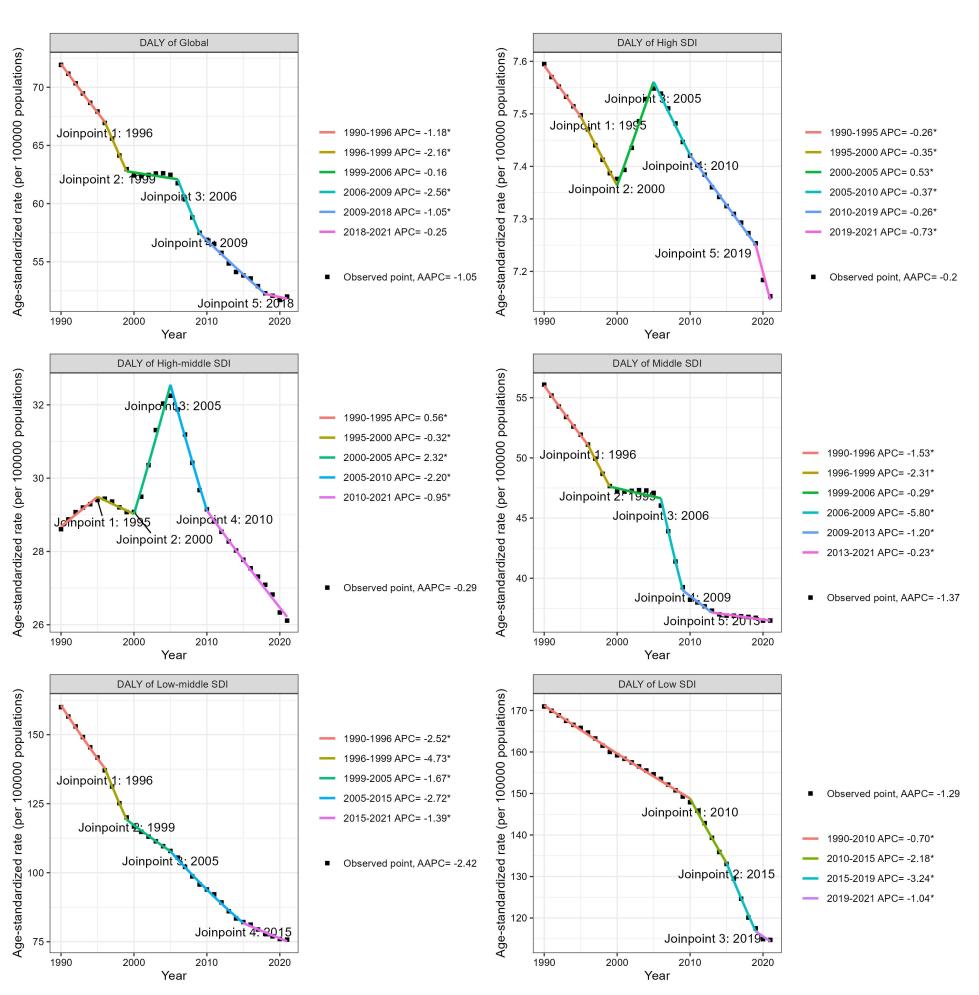


Supplementary Figure 5. Joinpoint regression analysis of temporal trends for age-standardized YLD rate of iodine deficiency among WRA globally and by SDI quintiles from 1990 to 2021. Rates are expressed per 100,000 populations. APC: annual percent change; AAPC: average annual percent change; SDI: socio-demographic index; YLD: years lived with disability

**
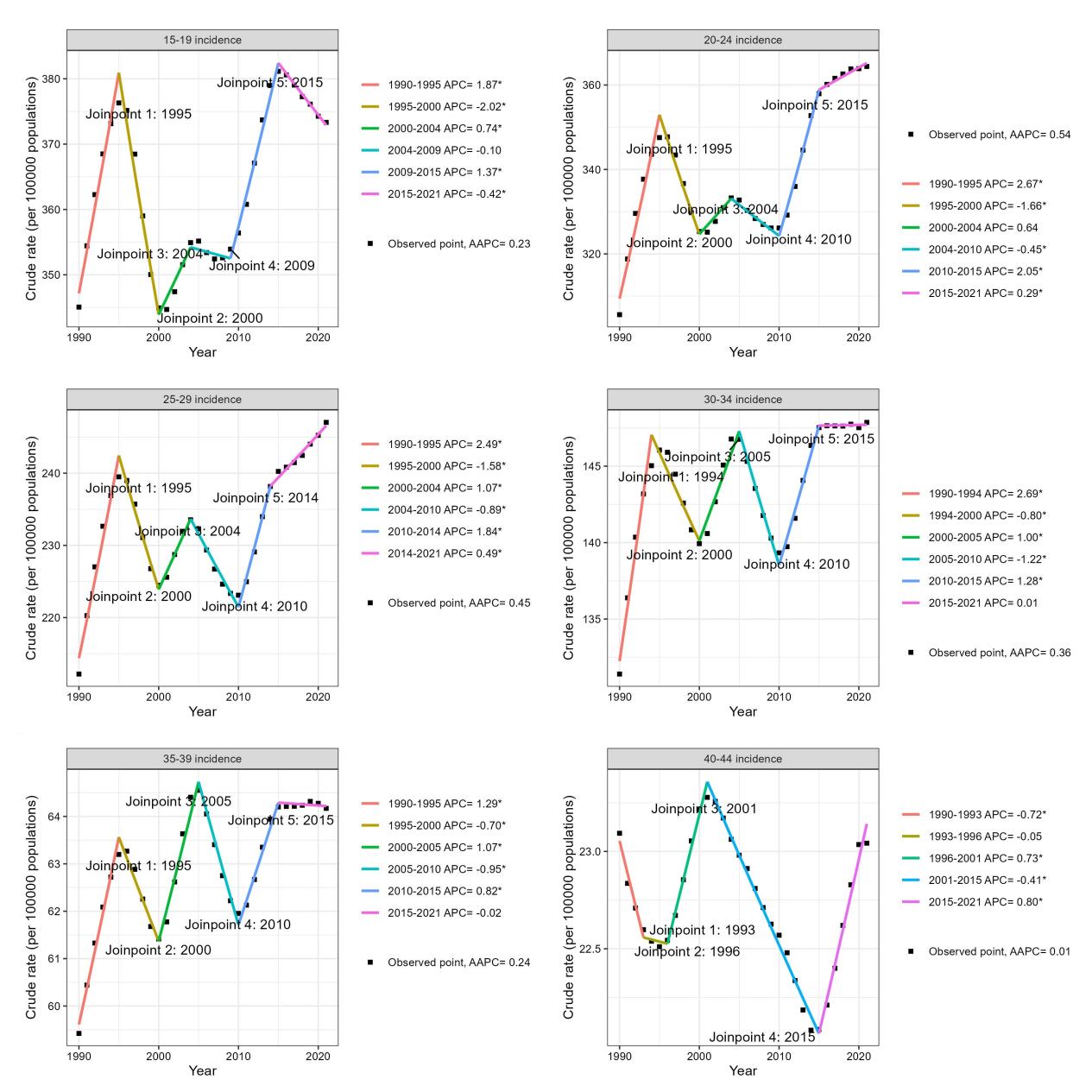
**

# Supplementary Figure 6. Joinpoint regression analysis of temporal trends of age-specific incidence rate for iodine deficiency globally by ages 1990 to 2021. APC: annual percent change; AAPC: average annual percent change.


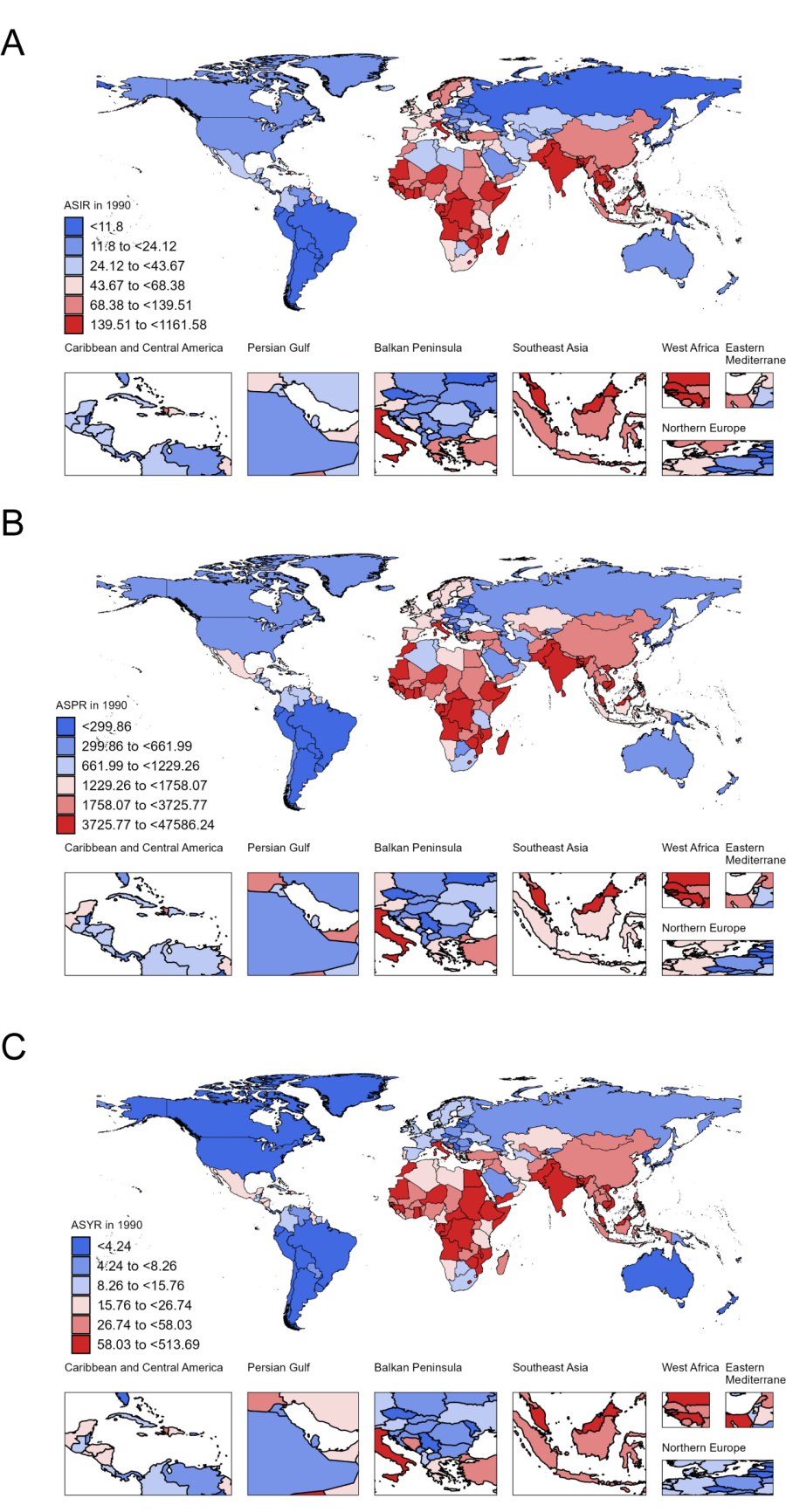


Supplementary Figure 7. Age-standardized disease burden of iodine deficiency for reproductive-age women across 204 countries and territories in 1990. (A) Age-standardized incidence rate; (B) Age-standardized prevalence rate; (C) Age-standardized YLD rate.


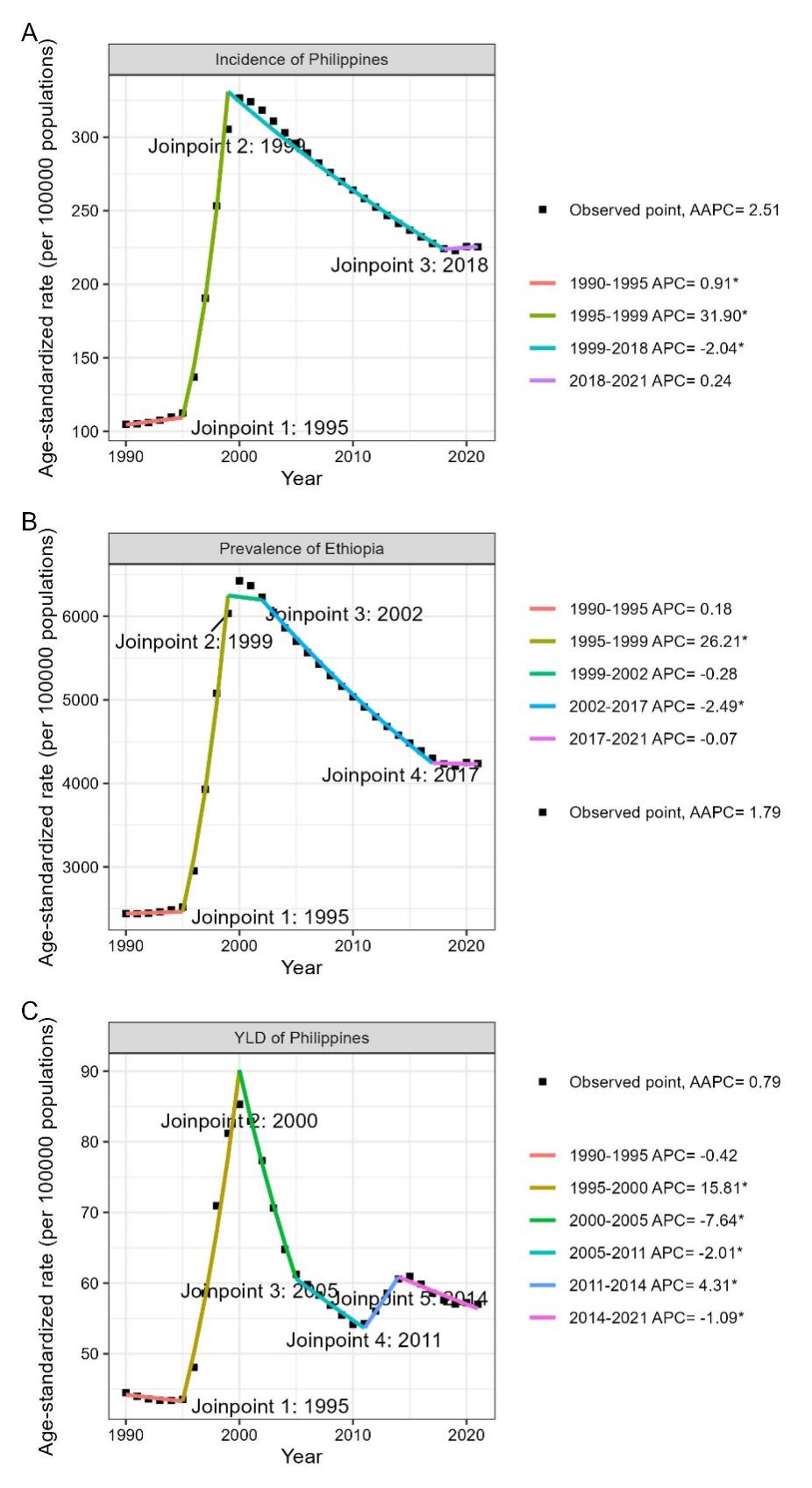


Supplementary Figure 8. Temporal trend of iodine deficiency burden in women of reproductive age in middle SDI country (Philippines) from 1990 to 2021. (A) ASIR; (B) ASPR; (C) ASYR. APC: annual percent change; AAPC: average annual percent change.


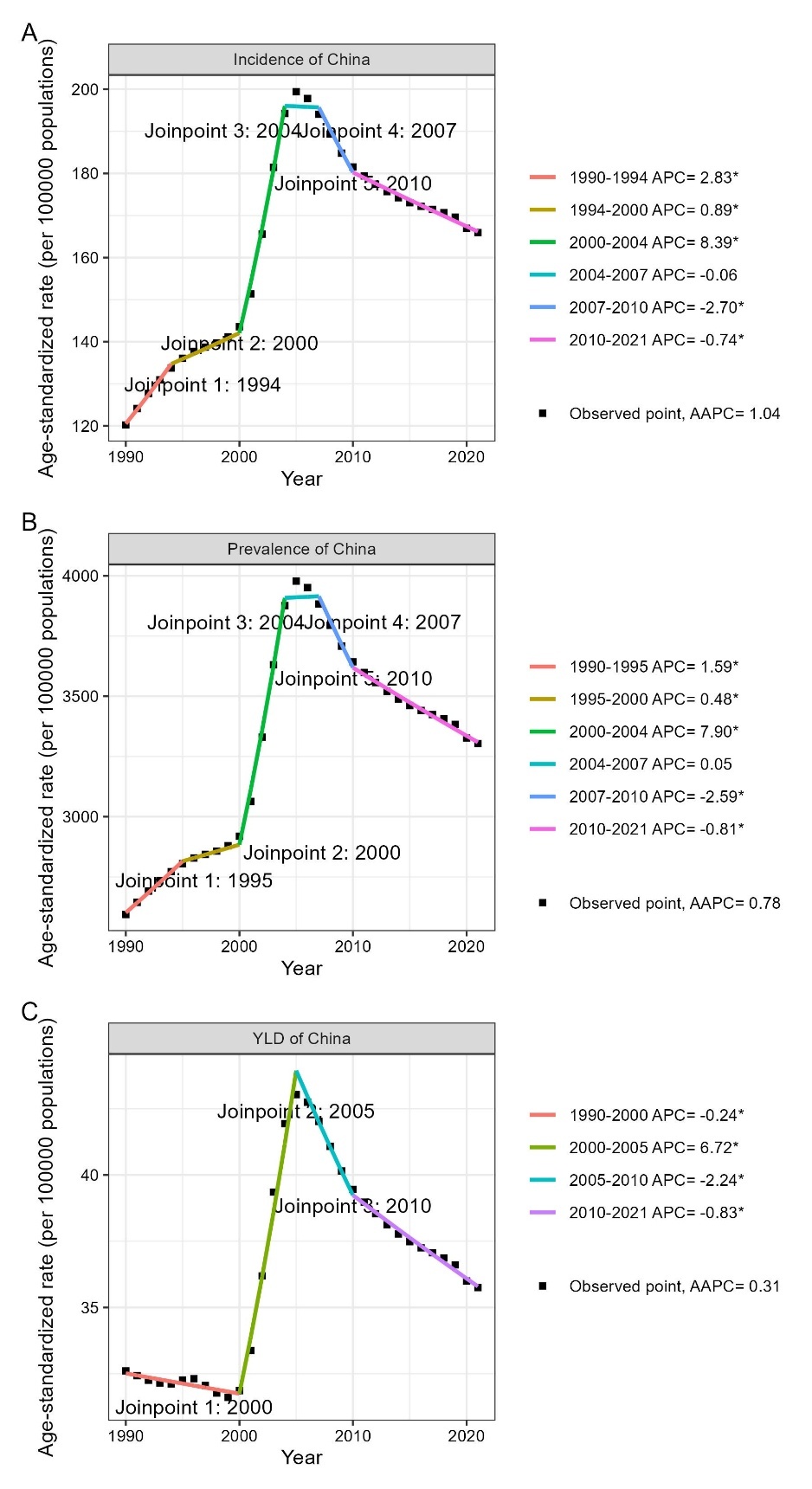


Supplementary Figure 9. Temporal trend of iodine deficiency burden in women of reproductive age in High-middle SDI country (China) from 1990 to 2021. (A) ASIR; (B) ASPR; (C) ASYR. APC: annual percent change; AAPC: average annual percent change.


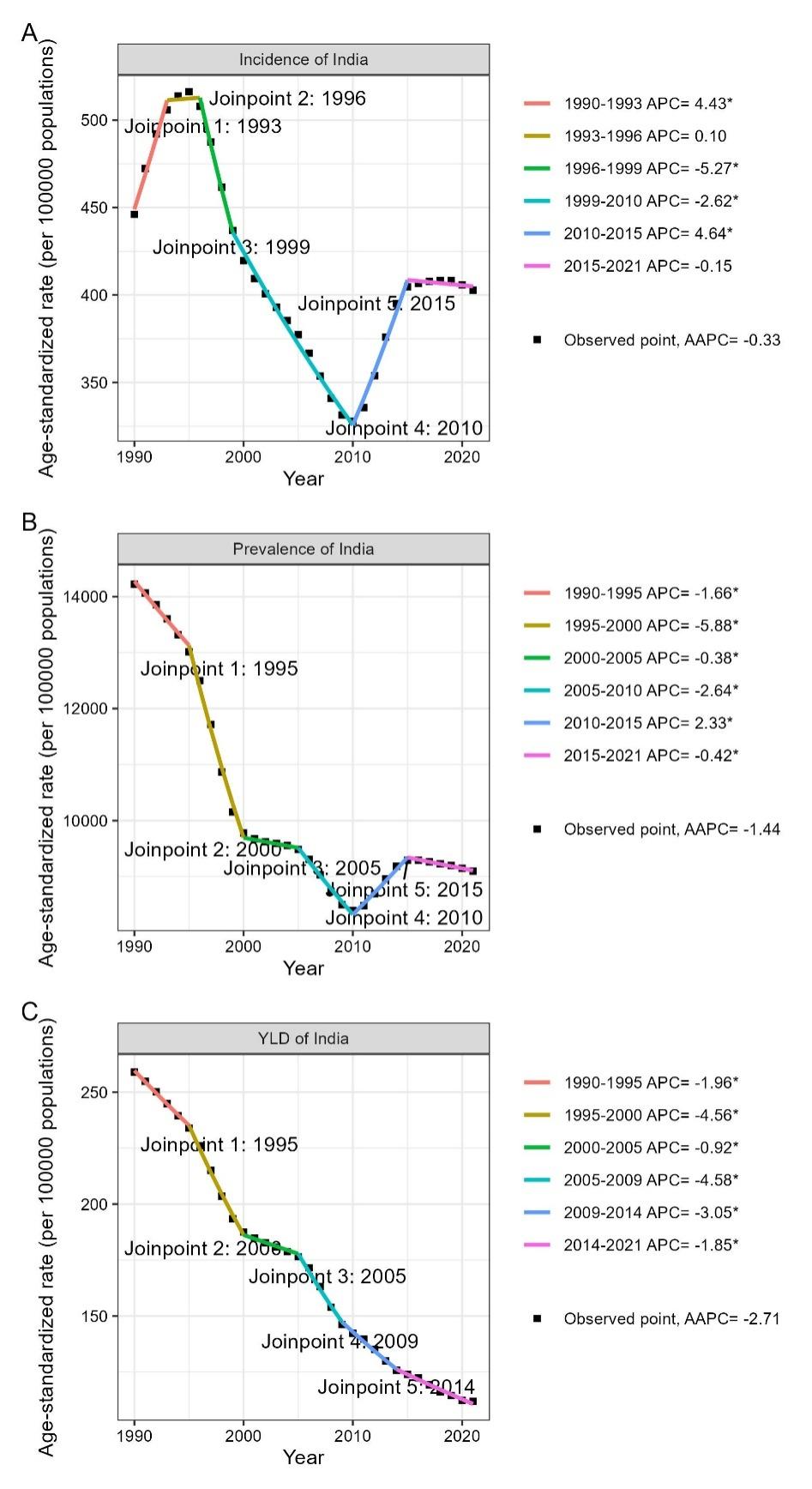


Supplementary Figure 10. Temporal trend of iodine deficiency burden in women of reproductive age in India of low-middle SDI country from 1990 to 2021. (A) ASIR; (B) ASPR; (C) ASYR.


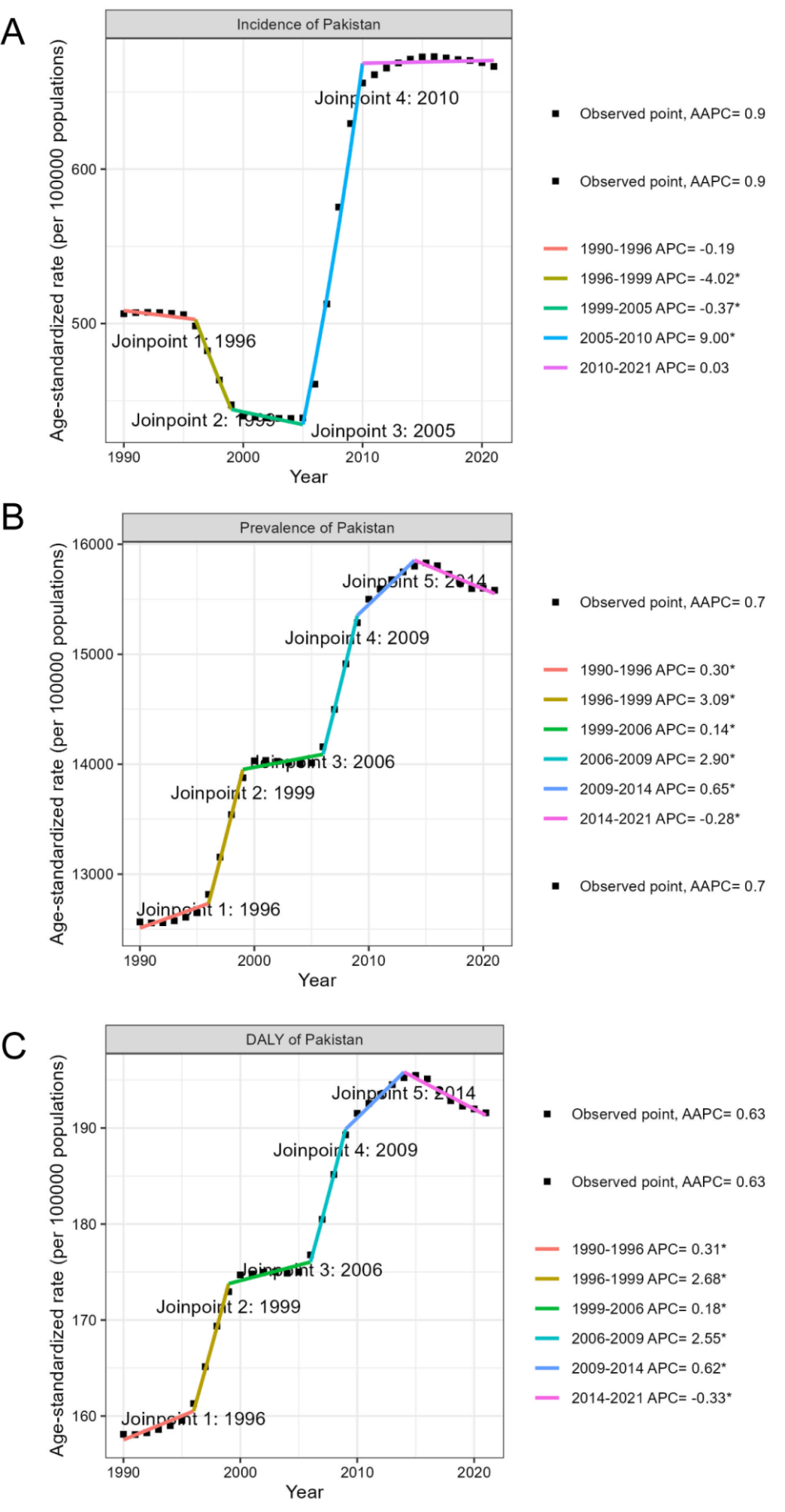


Supplementary Figure 11. Temporal trend of iodine deficiency burden in women of reproductive age in Pakistan of low-middle SDI country from 1990 to 2021. (A) ASIR; (B) ASPR; (C) ASYR


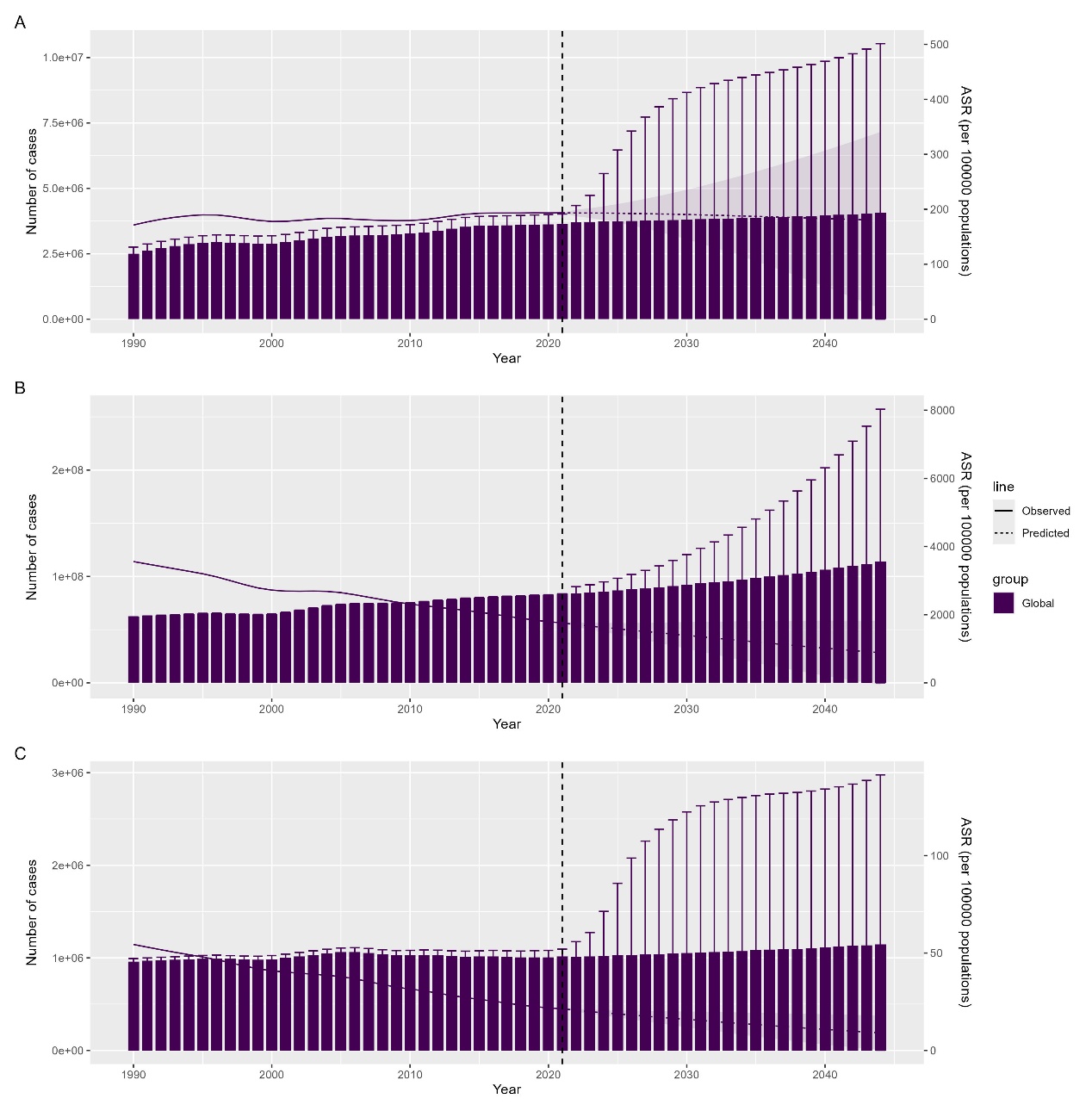


Supplementary Figure 12. The projections of global iodine deficiency burden among reproductive- age women from 2021 to 2044. (A) Incidence cases and age-standardized incidence rate; (B) Prevalence cases and age-standardized prevalence rate; (C) YLD and age-standardized YLD rate.
